# API5 Phosphorylation Promotes Antiviral Immunity by Inhibiting Degradation of Cytosolic RNA Sensor RLRs

**DOI:** 10.1002/advs.202505479

**Published:** 2025-07-11

**Authors:** Tingjuan Deng, Jianan Xu, Linglong Qin, Xingbo Wang, Chenhe Lu, Yanming Huang, Da Liu, Yan Yan, Weiren Dong, Pinglong Xu, Jiyong Zhou

**Affiliations:** ^1^ MOA Key Laboratory of Animal Virology Zhejiang University Center for Veterinary Sciences Hangzhou 310058 China; ^2^ State Key Laboratory for Diagnosis and Treatment of Severe Infectious Diseases First Affiliated Hospital Zhejiang University Hangzhou 310058 China; ^3^ Life Sciences Institute Zhejiang University Hangzhou 310058 China

**Keywords:** API5 phosphorylation, p62/SQSTM1, RLR antiviral signaling, ubiquitin‐mediated autophagy

## Abstract

Ubiquitin‐mediated selective autophagy is essential for innate immune responses against pathogens. However, the role of apoptosis inhibitor 5 (API5), in governing both ubiquitin‐mediated autophagy and antiviral immunity, are poorly defined. Here, it is found that the serine/arginine‐rich protein kinase 1 (SRPK1)‐dependent phosphorylation of API5 at S464 site is essential for priming antiviral immune responses during diverse RNA virus infection. Mechanistically, phosphorylated API5 forms complexes with autophagic receptor p62 and eliminates itself from ubiquitination at K141, thereby reducing p62 aggregations and inhibiting the autophagic degradation of cytosolic RNA sensors RIG‐I and MDA5 to mobilize RLR‐mediated antiviral responses. Taken together, it is unveiled that API5 phosphorylation by SRPK1 is required for the inhibition of ubiquitin‐mediated autophagic degradation of RNA sensors, revealing a coordinating nature of virus–host interactions that sustains host antiviral defenses.

## Graphical abstract

Ubiquitin‐mediated selective autophagy plays a critical role in antiviral innate immunity. The study shows that upon RNA virus infection, cytoplasmic API5 undergoes SRPK1‐dependent phosphorylation at S464, which in turn suppresses p62 K141 ubiquitination‐dependent autophagy. This inhibition prevents the degradation of cytoplasmic RNA sensors RIG‐I and MDA5, thereby potentiating antiviral innate immunity and host defense against viral infection.

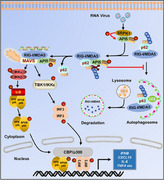

## 1 Introduction

The innate immune response stimulated by pathogen‐associated molecular patterns (PAMPs) instigates the expression of type I interferons (IFNs) and proinflammatory cytokines, thus constituting the initial defense line of the host.^[^ ^1^ ^]^ Viral nucleic acids are primary PAMPs that host cells detect upon viral invasion.^[^ ^2^ ^]^ Intracellular viral RNA is recognized by the retinoic acid‐inducible gene‐I (RIG‐I)‐like receptors (RLRs), including RIG‐I and melanoma differentiation‐associated protein‐5 (MDA5), which are typically expressed at low levels in most cell types and are upregulated following viral exposure.^[^ ^3^ ^]^ Studies have demonstrated that RIG‐I predominantly senses viral 5′‐ppp double‐strand (ds) RNA and short dsRNA, while MDA5 exhibits a higher affinity to long dsRNA.^[^ ^4^ ^]^ RIG‐I primarily recognizes RNA viruses including Sendai virus (SeV), vesicular stomatitis virus (VSV), Newcastle disease virus (NDV), and influenza virus (IAV), whereas MDA5 mainly detects picornaviruses like encephalomyocarditis virus (EMCV).^[^ ^5^, ^6^, ^7^ ^]^ Notably, certain viruses such as Dengue virus (DEN) and SeV can be detected by both RIG‐I and MDA5.^[^ ^8^, ^9^ ^]^ Upon binding to viral RNA, RIG‐I and MDA5 are recruited to mitochondrial antiviral signaling protein (MAVS). MAVS serves as a central hub for recruiting downstream signaling molecules, activating the kinases TANK‐binding kinase 1 (TBK1) and IkappaB kinases (IKKs).^[^ ^10^ ^]^ which further activate interferon regulatory factor 3 (IRF3) and nuclear factor‐kappaB (NF‐κB) and eventually induce downstream antiviral genes.^[^ ^11^, ^12^ ^]^

Autophagy stands as a fundamental cellular process involved in various aspects of cell physiology, including host defense against invading pathogens.^[^ ^13^ ^]^ Upon viral infection, the autophagic machinery is frequently mobilized and selectively degrades components of innate immune signaling pathways, including RIG‐I, MDA5, cyclic guanosine monophosphate‐adenosine monophosphate synthase (cGAS), MAVS, TNF receptor associated factor 6 (TRAF6), stimulator of interferon genes (STING), and IRF3, to regulate antiviral responses.^[^ ^14^, ^15^, ^16^, ^17^, ^18^, ^19^, ^20^, ^21^ ^]^ Intriguingly, accumulating evidence indicates that selective autophagic receptor p62 promotes the degradation of RIG‐I and MDA5.^[^ ^21^, ^22^, ^23^, ^24^, ^25^, ^26^, ^27^ ^]^ This finding raises a pivotal question: Does the host utilize regulatory mechanisms to suppress p62 activity, thereby safeguarding these frontline immune sentinels to sustain effective antiviral defense?

Numerous pivotal autophagy components are ubiquitinated,^[^ ^28^ ^]^ as exemplified by p62, which harbors multiple ubiquitination sites,^[^ ^29^, ^30^ ^]^ catalyzed by enzymes such as tripartite motif‐containing protein 21 (TRIM21),^[^ ^31^ ^]^ RING‐finger protein 166 (RNF166),^[^ ^32^ ^]^ neuronally expressed developmentally down‐regulated 4 (NEDD4),^[^ ^33^ ^]^ or kelch‐like ECH‐associated protein 1 (KEAP1)‐ cullin3 (CUL3),^[^ ^34^ ^]^ and counteracted by deubiquitinating enzymes (DUBs) like ubiquitin specific peptidase 8 (USP8) and ovarian tumor deubiquitinating enzyme 7b (OTUD7B).^[^ ^14^, ^35^ ^]^ OTUD7B is currently the only known deubiquitinase that negatively regulates antiviral immunity by inhibiting p62 ubiquitination at lysine (K) 7 to promote IRF3 degradation.^[^ ^14^ ^]^ Given that p62 activity is critically regulated by ubiquitination at specific sites, it remains unclear whether additional DUBs or functionally analogous proteins exist to deubiquitinate p62.

Apoptosis inhibitor 5 (API5), also known as anti‐apoptosis clone 11,^[^ ^36^ ^]^ sustains cell viability, particularly under serum and growth factor deprivation, potentially by hindering apoptosis induced by caspase‐3 or E2F transcription factor 1.^[^ ^37^, ^38^ ^]^ Acetylation of API5 at residue K251 regulates its anti‐apoptotic activity.^[^ ^39^ ^]^ Notably, API5 has been demonstrated to activate dendritic cells and enhance immune responses via Toll‐like receptor 4 (TLR‐4)‐NF‐κB signaling, while also targeting influenza A virus nucleoprotein (NP) to suppress viral replication.^[^ ^40^, ^41^ ^]^ Conversely, Heat Shock Protein 20 (Hsp20) interacts with API5 and downregulates its expression during white spot syndrome virus infection, thereby promoting viral replication.^[^ ^42^ ^]^ Our previous study also demonstrated that chicken API5 potentiates MDA5‐mediated activation of the IFN‐β promoter.^[^ ^43^ ^]^ These findings indicate that API5 may have broader roles in antiviral immunity. Intriguingly, recent work revealed that γδ intraepithelial lymphocyte‐derived API5 acts as an autophagy inhibitor to protect Paneth cell viability.^[^ ^44^ ^]^ Based on these observations, whether API5 may serve as a bridging molecule connecting autophagy and RLR‐mediated antiviral signaling is unknown.

This study aimed to investigate whether API5 modulates RLR antiviral signaling via ubiquitin‐dependent autophagy and to elucidate the underlying molecular mechanisms of API5 regulation. Here, we revealed that diverse RNA virus infection promotes API5 phosphorylation at serine (S) 464 by SRPK1. Phosphorylated API5 protein (pAPI5) subsequently forms complexes with p62, hindering its ubiquitination at residue K141. This unanticipated regulation reduces p62 aggregations and impedes the autophagic degradation of cytosolic RNA sensors RIG‐I and MDA5. Consequently, this triggers RLR‐mediated antiviral responses, safeguarding the host against virus infection.

## 2 Results

### 2.1 API5 Positively Governs RLR‐Mediated Innate Antiviral Immunity

**Figure 1:**
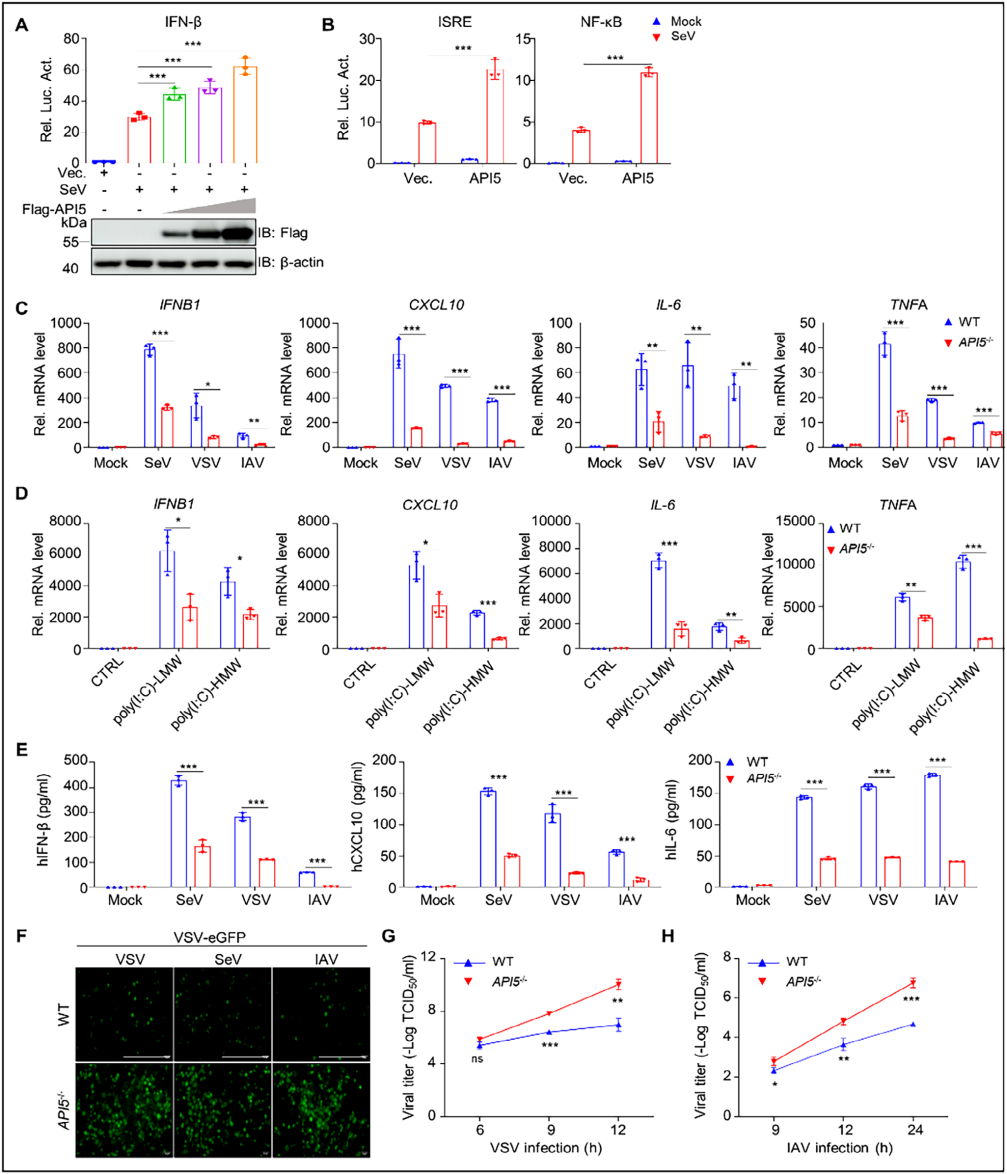
API5 is a novel positive regulator of innate antiviral immunity. A and B) HEK293T cells were co‐transfected with IFN‐β‐Luc (A), ISRE, or NF‐κB‐Luc reporter (B), pRL‐TK, and API5 plasmid for 24 h, followed by infection with SeV (25 HA units) for 12 h. Luciferase activity was detected by the dual‐luciferase assay. C and D) WT and *API5* ^−/−^ A549 cells were infected with SeV (25 HA units), VSV (MOI = 0.1), and IAV (MOI = 3) for 9 h (C), or were stimulated with poly(I:C)‐HMW (high molecular weight) or poly(I:C)‐LMW (low molecular weight) for 6 h (D). The levels of *IFNB*1, *CXCL*10, *IL*‐6, and *TNF*A mRNA were detected by qPCR. E) WT and *API5* ^−/−^ A549 cells were infected with SeV (25 HA units), VSV (MOI = 0.1), and IAV (MOI = 3) for 9 h. The levels of IFN‐β, CXCL10, and IL‐6 protein were analyzed by ELISA. F) WT and *API5* ^−/−^ A549 cells were infected with SeV (25 HA units), VSV (MOI = 0.1), and IAV (MOI = 3) for 9 h. The supernatant was harvested and treated by UV irradiation for 30 min, and then incubated with fresh A549 cells for 24 h. The resultant cells were infected with VSV‐eGFP (MOI = 0.1). At 12 h after infection, the cells were observed under fluorescence microscopy. Scale bar, 50 µm. G and H) WT and *API5* ^−/−^ A549 cells were infected with VSV (MOI = 0.1) (G) or IAV (MOI = 3) (H). At indicated time points, the supernatant was harvested to examine viral titer using TCID_50_ assay. For (F), data are one representative of three biological replicates. For (A–E,G,H), data were shown as mean ± SEM (n = 3 biological replicates). Statistical Significance was analyzed by unpaired two‐tailed Student's *t*‐test. (*p < 0.05; **p < 0.01; ***p < 0.001; ns, no significant).

Previous studies have demonstrated that API5 regulates TLR‐4‐NF‐κB signaling and viral replication,^[^ ^40^, ^41^, ^42^, ^43^ ^]^ implicating its potential involvement in antiviral immunity. To investigate the functions of API5 in innate antiviral responses, we first examined the effects of API5 overexpression on SeV‐driven activation of IFN‐β, ISRE, and NF‐κB promoters using reporter assays. As depicted in **Figure** **1**A,B, API5 overexpression significantly potentiated SeV‐triggered activation of these promoters. To further validate these findings, we constructed *API5* knockout (KO) (*API5* ^−/−^) A549 cell line (Figure S1A–C, Supporting Information) and quantified antiviral gene expression upon various stimulation. Quantitative PCR (qPCR) analysis demonstrated that that *API5* KO dramatically inhibited the transcription of *IFNB1*, *CXCL10*, *IL‐6*, and tumor necrosis factor‐alpha (*TNF*A) following stimulation with SeV, VSV, IAV, poly(I:C)‐LMW (low molecular weight), and poly(I:C)‐HMW (high molecular weight) (Figure 1C,D). Similar results were observed in *API5* knockdown (sh*API5*; 92.4% reduction) THP‐1 cells infected with SeV (Figure S1D–F, Supporting Information). Additionally, ELISA confirmed that *API5* KO markedly reduced SeV‐, VSV‐, or IAV‐induced secretion of IFN‐β, IL‐6, and CXCL10 (Figure 1E). Further analysis demonstrated that *API5* KO significantly inhibited IFN production induced by SeV, VSV, and IAV (Figure 1F) and enhanced VSV and IAV replication in A549 cells (Figure 1G,H; Figure S1G, Supporting Information). These results collectively indicate that API5 acts as a positive regulator of RLR‐mediated innate antiviral immune responses, enhancing host defense against diverse RNA viruses by promoting antiviral gene expression.

**Figure 2:**
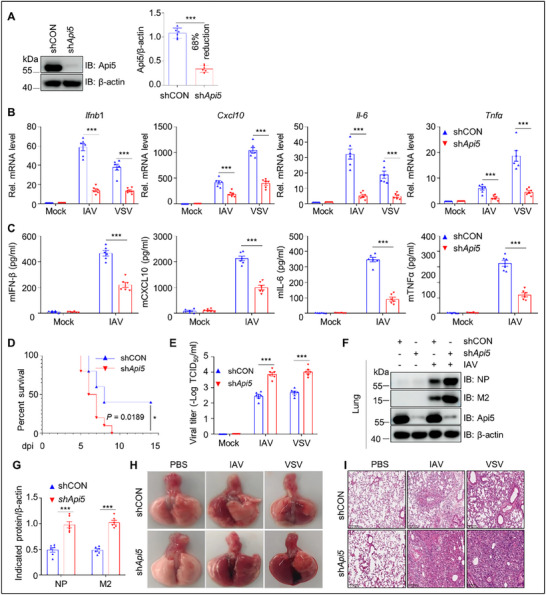
Api5 is crucial for facilitating innate antiviral response in mice. A) Left: Immunoblotting analysis of Api5 in mouse lungs (n = 6 mice/group) infected with AAV6‐shCON or AAV6‐sh*Api5* (10^11^ TCID_50_ per mouse; 60 µL) for four weeks. Right: data were quantified and shown as the ratio of Api5 to β‐actin. B) The shCON and sh*Api5* mice lungs (n = 6 mice/group) infected with IAV (5×10^5^ PFU mL^−1^; 50 µL per mouse) or VSV (10^8.0^ PFU mL^−1^; 60 µL per mouse) at 4 dpi were subjected to qPCR analysis for mRNAs of *Ifnb1*, *Cxcl10*, *Il*‐*6* and *Tnfa*. C) The BAL from IAV‐ or mock‐infected shCON and sh*Api5* mice (n = 6 mice/group) at 4 dpi were used for ELISA analysis of IFN‐β, CXCL10, IL‐6, and TNFα. D) The shCON and sh*Api5* mice (n = 10 mice/group) were intranasally inoculated with IAV and monitored for 14 days for survival. Kaplan‐Meier survival curves were compared using log‐rank (Mantel‐Cox) analysis. E) Virus titers of IAV‐ or VSV‐infected shCON and sh*Api5* mice lungs (n = 6 mice/group) were detected by TCID_50_ assays. F) Viral proteins of IAV‐infected shCON and sh*Api5* mice lungs (n = 6 mice/group) were immunoblotted with indicated antibodies. G) Data in (F) were quantified and shown as the ratio of NP to β‐actin and M2 to β‐actin. H) Gross images of lungs of IAV‐ or VSV‐infected shCON and sh*Api5* mice (n = 6 mice/group) at 4 dpi. The images are from representative one of six mice per group. I) Hematoxylin‐eosin (HE) staining of mice lung from IAV‐ or VSV‐infected shCON and sh*Api5* mice (n = 6 mice/group) at 4 dpi. Scale bar, 200 µm. The images are from representative one of six mice per group. Data are presented as the mean ± SEM (n = 6), and statistical significance was determined by unpaired two‐tailed Student's t test. (**p < 0.01; ***p < 0.001).

To substantiate the role of API5 under physiological conditions, we generated *Api5* ^−/−^ mice with a 2329 bp chromosomal deletion (Figure S2A,B, Supporting Information). However, very few *Api5* ^−/‐^ offspring mice were obtained from the *Api5* ^−/+^ breeder due to *Api5* knockout mice being prone to death, consistent with a previous report.^[^ ^44^ ^]^ To circumvent this limitation, we employed AAV6‐mediated shRNA delivery to achieve 68% *Api5* knockdown in mouse lungs (**Figure** **2**A). qPCR and ELISA analyses showed that *Api5* deficiency markedly diminished mRNA and protein levels of *Ifnb1*, *Cxcl*10, *Il*‐6, and *Tnfα* in IAV‐ or VSV‐infected mouse lung tissues and bronchoalveolar lavage fluid (BAL) (Figure 2B,C; Figure S2C,D, Supporting Information). These results indicate that *Api5* deficiency inhibits innate antiviral immunity in mice. Notably, *Api5*‐knockdown mice exhibited higher mortality following IAV infection compared to the control shRNA‐treated mice (Figure 2D), along with elevated IAV and VSV titers in lung tissues (Figure 2E–G; Figure S2E, Supporting Information) and more severe gross and histopathological lung lesions (Figure 2H,I; Figure S2F,G, Supporting Information). Collectively, our findings demonstrate that depletion of *Api5* in mice inhibits innate antiviral immune responses, thereby promoting viral replication and exacerbating virus‐induced lung pathology in vivo. These results suggest that API5 is a positive regulator of RNA virus‐triggered antiviral immunity.

### 2.2 API5 Potentiates RNA Virus‐Induced Antiviral Immunity at the Level of RIG‐I and MDA5

**Figure 3:**
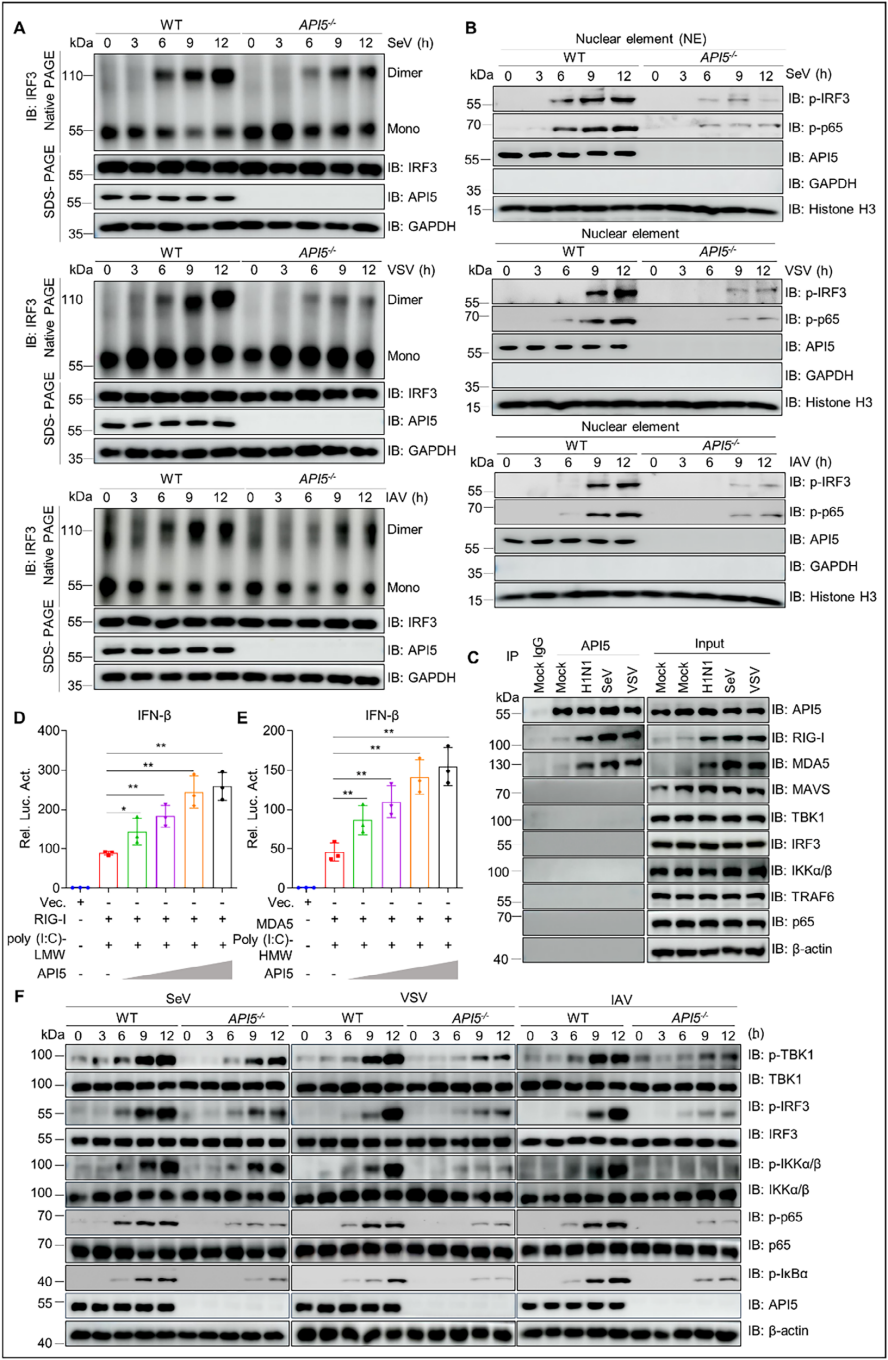
API5 positively regulates RLRs‐mediated innate antiviral response. A and B) WT and *API5* ^−/−^ A549 cells were infected with SeV (25 HA units), VSV (MOI = 0.1), or IAV (MOI = 3), as described in the method. The resultant cells were subjected to Native‐PAGE, subcellular fractionation, and immunoblotting with anti‐IRF3 rabbit pAb (A) and anti‐p‐IRF3 and p‐p65 rabbit pAbs (B). GAPDH and Histone H3 expression served as loading controls for cytoplasmic element (CE) and nuclear element (NE), respectively. C) A549 cells were infected with (25 HA units), VSV (MOI = 0.1), and IAV (MOI = 3) infection for 12 h. The lysates were subjected to co‐immunoprecipitation assays with mouse anti‐API5 monoclonal antibody or mouse IgG. Subsequently, the immunoprecipitation complexes were immunoblotted using indicated antibodies. D and E) IFN‐β reporter, pRL‐TK, Flag‐RIG‐I D) or MDA5 (E), and different dose API5 plasmids were co‐transfected into HEK293T cells for 24 h, and then the cells were separately treated with poly(I:C)‐LMW and poly(I:C)‐HMW for 12 h. The lysates were subjected to IFN‐β activity analysis. F) WT and *API5* ^−/−^A549 cells were infected with SeV, VSV, and IAV, as described in the method. The cellular lysates were subjected to the analysis of phosphorylation levels of TBK1, IRF3, IKKα/β, p65 and IκBα with indicated antibodies. For A‐C and F, data are one representative of three biological replicates. For D and E, data were shown as mean ± SEM (n = 3 biological replicates). Statistical Significance was analyzed by unpaired two‐tailed Student's t test. (*p < 0.05; **p < 0.01).

The RLR‐mediated antiviral immune pathway critically depends on IRF3 and NF‐κB signaling to induce IFNs and proinflammatory cytokines.^[^ ^1^ ^]^ Upon binding to the viral RNA, the RLR is recruited to the adaptor protein MAVS, triggering to the phosphorylation and dimerization of IRF3, which leads to IRF3 translocation to the nucleus to activate the IFN and a wide array of ISGs.^[^ ^45^ ^]^ Therefore, to elucidate the molecular mechanisms underlying API5‐mediated enhancement of antiviral immunity, we first examined IRF3 dimer formation in *API5* KO and wild‐type (WT) A549 cells, and observed a notable reduction of IRF3 dimers in SeV‐, VSV‐ or IAV‐infected *API5* ^−/−^ cells compared to WT cells (**Figure** **3**A; Figure S3A, Supporting Information). Subcellular fractionation further revealed that *API5* deficiency impaired nuclear translocation of IRF3, as evidenced by both decreased phosphorylation in the nucleus and concomitant accumulation in the cytoplasm following infection with SeV, VSV, or IAV (Figure 3B; Figure S3B–D, Supporting Information). Parallel analysis of NF‐κB signaling revealed that *API5* knockout also diminished phosphorylation of p65, a critical NF‐κB subunit required for nuclear translocation of the p65/p50 heterodimer and subsequent induction of IFNs and proinflammatory cytokines^[^ ^1^ ^]^ (Figure 3B; Figure S3B, Supporting Information). These results indicate that API5 enhances innate antiviral immunity by facilitating the activation of both IRF3 and NF‐κB signaling in the RLRs‐mediated immune pathway.

To determine which component of IRF3 and NF‐κB signaling pathway is targeted by API5, we performed co‐immunoprecipitation (Co‐IP) and confocal microscopy assays. Our results showed that API5 specifically interacted with RIG‐I and MDA5, but not with downstream signaling molecules (MAVS, TBK1, IRF3, TRAF6, IKKα, IKKβ, and p65) during SeV, VSV, or IAV infection (Figure 3C). Confocal microscopy confirmed the colocalization of cytoplasmic API5 with RIG‐I and MDA5 in IAV‐infected A549 cells (Figure S4A, Supporting Information). Domain mapping analysis identified residues 454–475 of API5 as critical for its binding to both RIG‐I and MDA5 (Figure S4B–E, Supporting Information). Functional analysis showed that API5 overexpression enhanced poly(I:C)‐triggered activation of IFN‐β, ISRE, and NF‐κB promoters when mediated by RIG‐I or MDA5 not by MAVS, TBK1, IRF3, TRAF6, IKKα/β, or p65 (Figure 3D,E; Figure S5A,B, Supporting Information). Consistent with this, *API5* deficiency significantly impaired the phosphorylation of TBK1, IRF3, IKKα/β, and IκBα following stimulation with SeV, VSV, IAV, or poly(I:C) (Figure 3F; Figure S5C–E, Supporting Information). Collectively, these findings suggest that API5 positively regulates RLR signaling at the level of RIG‐I and MDA5, and this effect is largely dependent on the cytoplasmic API5.

### 2.3 SRPK1 Phosphorylates API5 at S464 to Promote RNA Virus‐Triggered Immunity Response

API5 was initially identified as a nuclear protein.^[^ ^36^ ^]^ Our previous work established SUMOylation at K404 regulates its nuclear localization,^[^ ^43^ ^]^ suggesting that cytoplasmic API5 may represent an alternative posttranslational modification state. Phosphorylation has been widely reported as a key regulatory mechanism for the cytoplasmic/nuclear localization of substrate proteins.^[^ ^46^, ^47^, ^48^, ^49^, ^50^, ^51^ ^]^ Strikingly, a recent study demonstrated that phosphorylation controls the cytoplasmic retention of coactivator‐associated arginine methyltransferase 1 (CARM1), a predominantly nuclear protein.^[^ ^52^ ^]^ Additionally, phosphorylation of components in the antiviral immunity pathway is essential for activating antiviral signaling.^[^ ^53^, ^54^, ^55^ ^]^ Based on these findings, we investigated whether API5 is subject to phosphorylation. Mass spectrometry analysis of Flag‐API5 immuno‐precipitates identified S464 as a phosphorylation site (Figure S6A, Supporting Information). We developed a phospho‐specific rabbit polyclonal antibody (pAPI5) that selectively recognized the phosphorylated S464 peptide of API5 without cross‐reacting with its non‐phosphorylated counterpart (Figure S6B, Supporting Information).

**Figure 4:**
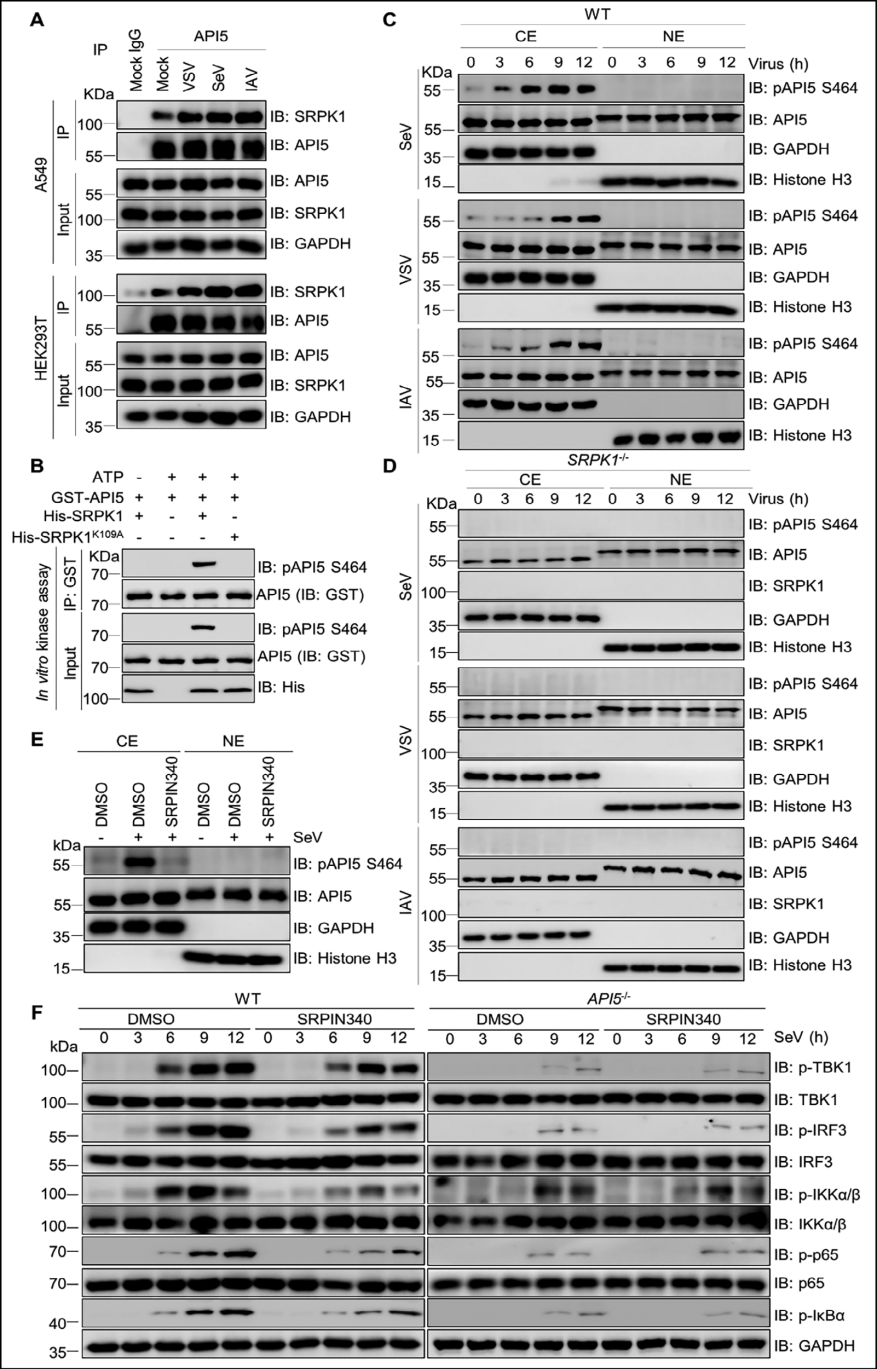
SRPK1 interacts with and phosphorylates API5 for promoting antiviral immune signaling. A) A549 cells and HEK293T cells were infected with SeV, VSV, and IAV infection for 12 h. The lysates were subjected to co‐immunoprecipitation using mouse anti‐API5 or mouse IgG, and the immunoprecipitation complex was immunoblotted with rabbit anti‐API5 and anti‐SRPK1 antibodies. B) GST‐API5(40 µg) and His‐SRPK1 or kinase‐dead mutant His‐SRPK1^K109A^ (40 µg) from *E. coli* BL21 were co‐incubated with 500 µL kinase buffer, and then in vitro kinase assays were performed as described in the method. The reaction products were analyzed using immunoblotting assays with the pAPI5 antibody. C and D) SeV, VSV, and IAV‐infected WT (C) and *SRPK1* KO (D)A549 cells were subjected to cellular fractionation and immunoblotting with indicated antibodies. GAPDH and Histone H3 were used as markers of the cytosolic elements (CE) and nuclear elements (NE), respectively. E) A549 cells were treated with SRPK1 kinase inhibitor SRPIN340 (10 µm) for 6 h, and then were infected by SeV for 12 h. The cellular lysates were used for cellular fractionation and immunoblotting with pAPI5. GAPDH and Histone H3 were markers of cytoplasmic elements (CE) and nuclear elements (NE), respectively. F) WT and *API5* ^−/−^ A549 cells were pretreated with DMSO or SRPIN340 (10 µm) for 6 h before SeV infection, and then the cellular lysates were subjected to immunoblotting of phosphorylated TBK1, IRF3, IKKα/β, p65vand IκBα. Data are one representative of three biological replicates.

To identify the kinase(s) that specifically phosphorylates S464 on API5, we screened candidate kinases predicted by GPS 6.0 (http://gps.biocuckoo.cn/online.php) via co‐IP assays. Among the interactors (SRPK1, PRKCI, MARK1, CDK8, CLK1, and CSNK2A1; Figure S7, Supporting Information), SRPK1 emerged as the most likely candidate due to its known preference for the “S^464^PPK” motif in API5.^[^ ^56^ ^]^ Subsequent mass spectrometry, co‐IP, and pull‐down assays confirmed SRPK1 as a direct binding partner of API5 (Figure S8A–C, Supporting Information). Notably, endogenous API5‐SRPK1 interaction was enhanced by SeV, VSV or IAV infection (**Figure** **4**A; Figure S8D, Supporting Information). Using the pAPI5 S464 antibody, we confirmed that SRPK1, but none of the other interacting kinases, specifically phosphorylated API5 at S464 during IAV infection (Figure S8E,F, Supporting Information). In vitro kinase assays further demonstrated that catalytically active SRPK1 (but not the kinase‐dead K109A mutant^[^ ^57^ ^]^) phosphorylated recombinant API5 (Figure 4B), solidifying SRPK1 as the direct kinase for phosphorylating API5 at S464.

To confirm if phosphorylation regulates API5 cytoplasmic localization and its role in innate antiviral immunity, we performed subcellular fractionation of SeV‐, VSV‐, and IAV‐infected A549 cells. The results showed that total API5 levels remained unchanged, and cytoplasmic pAPI5 progressively increased during virus infection (Figure 4C; Figure S8G, Supporting Information). Notably, this phosphorylation was completely abolished in *SRPK1* knockout cells, regardless of infection status (Figure 4D; Figure S8H, Supporting Information). Moreover, the SRPK1‐specific inhibitor SRPIN340 effectively blocked SeV‐induced API5 phosphorylation (Figure 4E; Figure S8I, Supporting Information), further confirming SRPK1 as the primary kinase responsible for cytoplasmic API5 phosphorylation. Consistent with this, both SRPIN340 treatment and *SRPK1* deficiency significantly attenuated SeV‐, VSV‐, and IAV‐induced expression of antiviral cytokines (*IFNB1*, *CXCL10*, *IL‐6*, and *TNFA*) (Figure S9A,B, Supporting Information). Critically, SRPIN340‐mediated SRPK1 inhibition reduced SeV‐driven phosphorylation of TBK1, IRF3, IKKα/β, p65, and IκBα molecules in WT cells but not *API5* ^−/‐^ cells (Figure 4F; Figure S10, Supporting Information). These results demonstrated that SRPK1‐mediated phosphorylation of cytoplasmic API5 at S464 is indispensable for the initiation of host antiviral defense mechanisms during RNA virus infection.

### 2.4 Phosphorylation of API5 Prerequisites for the Activation of RLRs‐Dependent Innate Antiviral Immunity

**Figure 5:**
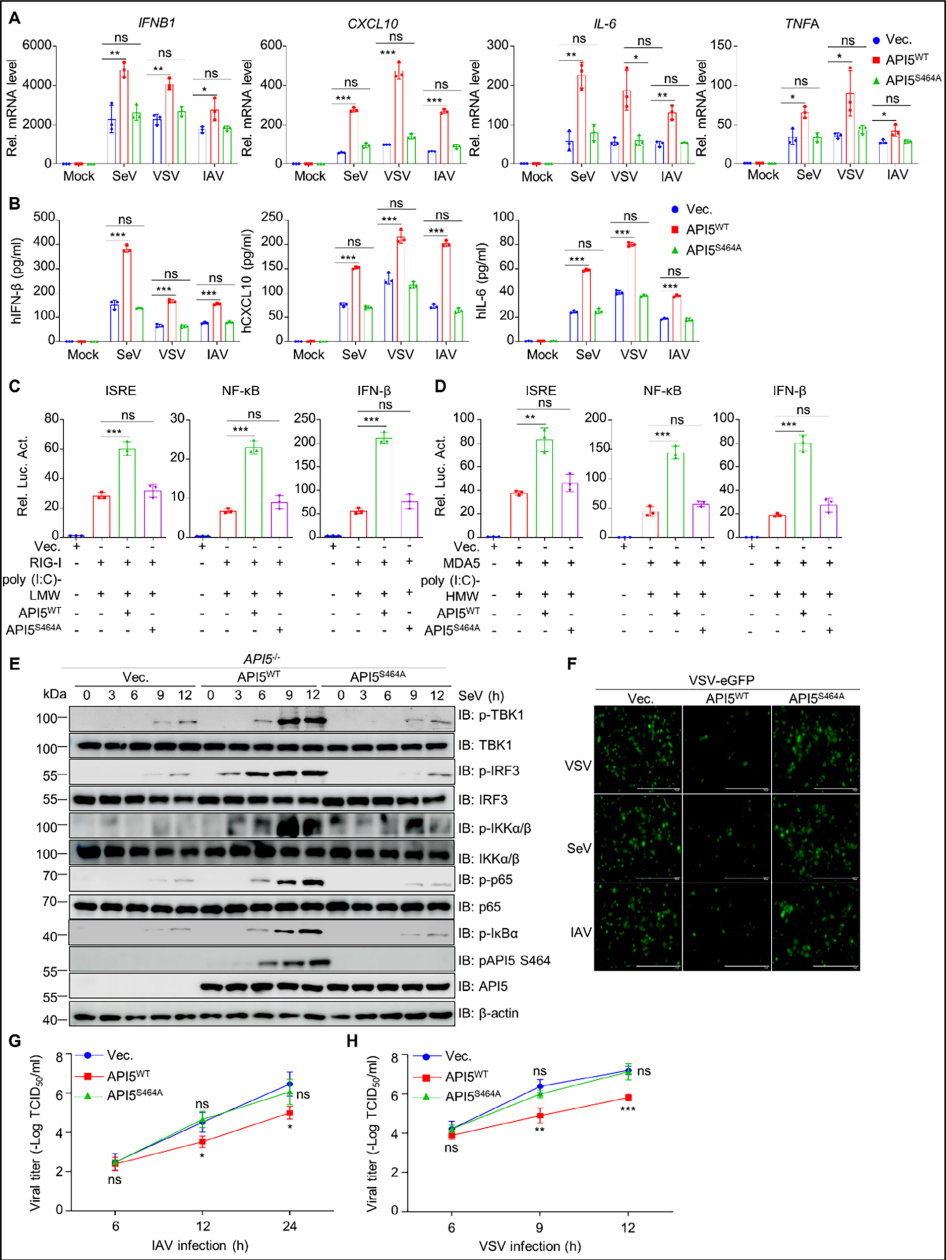
Phosphorylation of API5 is crucial for facilitating innate antiviral immunity in vitro. A,B) *API5* ^−/−^ A549 cells were transfected with Flag‐tagged API5^WT^ or API5^S464A^ for 36 h, followed by infection with SeV, VSV, or IAV for 9 h. The lysates and culture media underwent qPCR (A) and ELISA (B) to analyze the mRNA and protein levels of IFN‐β, CXCL10, IL‐6, and TNF‐α, respectively. C,D) HEK293T cells were co‐transfected with ISRE, NF‐κB, or IFN‐β reporter plasmids along with pRL‐TK, Flag‐RIG‐I (C), or Flag‐MDA5 (D), and API5^WT^, API5^S464A^ for 24 h, and then the cells were separately treated with poly(I:C)‐LMW and poly(I:C)‐HMW for 12 h. The lysates were subjected to luciferase reporter assays. E) *API5* ^−/−^ A549 cells were transfected with Flag‐tagged API5^WT^ or API5^S464A^ for 24 h, followed by infection with SeV, VSV, and IAV. The cellular lysates were subjected to immunoblotting to analyze the levels of phosphorylated and total TBK1, IRF3, IKKα/β, and IκBα. F) *API5* ^−/−^ A549 cells were transfected and infected as described in (E). At 9 h after infection, the supernatant was harvested and treated for 30 min by UV irradiation and then incubated with fresh A549 cells for 24 h. The resultant cells were infected with VSV‐eGFP. At 12 h after infection, VSV‐eGFP replication was detected by fluorescence microscopy. Scale bar, 50 µm. G and H) *API5* ^−/−^ A549 cells were transfected as described in (E) and then infected with IAV (G) or VSV (H). The supernatant was harvested to evaluate the viral titer of IAV and VSV by TCID_50_ assays. For E and F, data are one representative of three biological replicates. For (A–D,G,H), data were shown as mean ± SEM (n = 3 biological replicates). Statistical Significance was analyzed by unpaired two‐tailed Student's t test. (*p < 0.05; **p < 0.01; ***p < 0.001; ns, no significant).

To further elucidate if the regulation of API5 on antiviral immune signaling is relevant to its phosphorylation, we measured type I IFN and pro‐inflammatory cytokines in *API5* ^−/−^ A549 cells transfected with plasmid encoding either API5^WT^ or the phosphorylation‐deficient mutant API5^S464A^. qPCR and ELISA assays revealed that exogenous API5^WT^, but not API5^S464A^, significantly increased *IFNB1*, *CXCL10*, *IL‐6*, and *TNFα* mRNA and protein levels upon SeV, VSV, or IAV infection (**Figure** **5**A,B). Reporter assays further demonstrated that API5^WT^ overexpression, but not API5^S464A^, dramatically enhanced poly (I:C)‐driven RIG‐I‐ and MDA5‐mediated ISRE, NF‐κB, and IFN‐β promoter activities (Figure 5C,D). Additionally, SeV‐induced phosphorylation of TBK1, IRF3, IKKα/β, p65, and IκBα was elevated in *API5*‐deficient A549 cells reintroduced with API5^WT^ but not API5^S464A^ (Figure 5E; Figure S11, Supporting Information). These results collectively indicate that S464 phosphorylation is essential for API5's positive regulation of antiviral signaling. Consistently, IFN sensitivity assays revealed that API5^WT^, but not API5^S464A^, promoted the secretion of IFN‐I induced by SeV, VSV, and IAV (Figure 5F). Moreover, viral replication kinetics showed significantly reduced IAV and VSV replication in cells expressing API5^WT^, but not API5^S464A^, compared to the control cells (Figure 5G,H). In summary, these findings establish that pAPI5 is indispensable for priming antiviral innate immunity against RNA virus infection.

**Figure 6:**
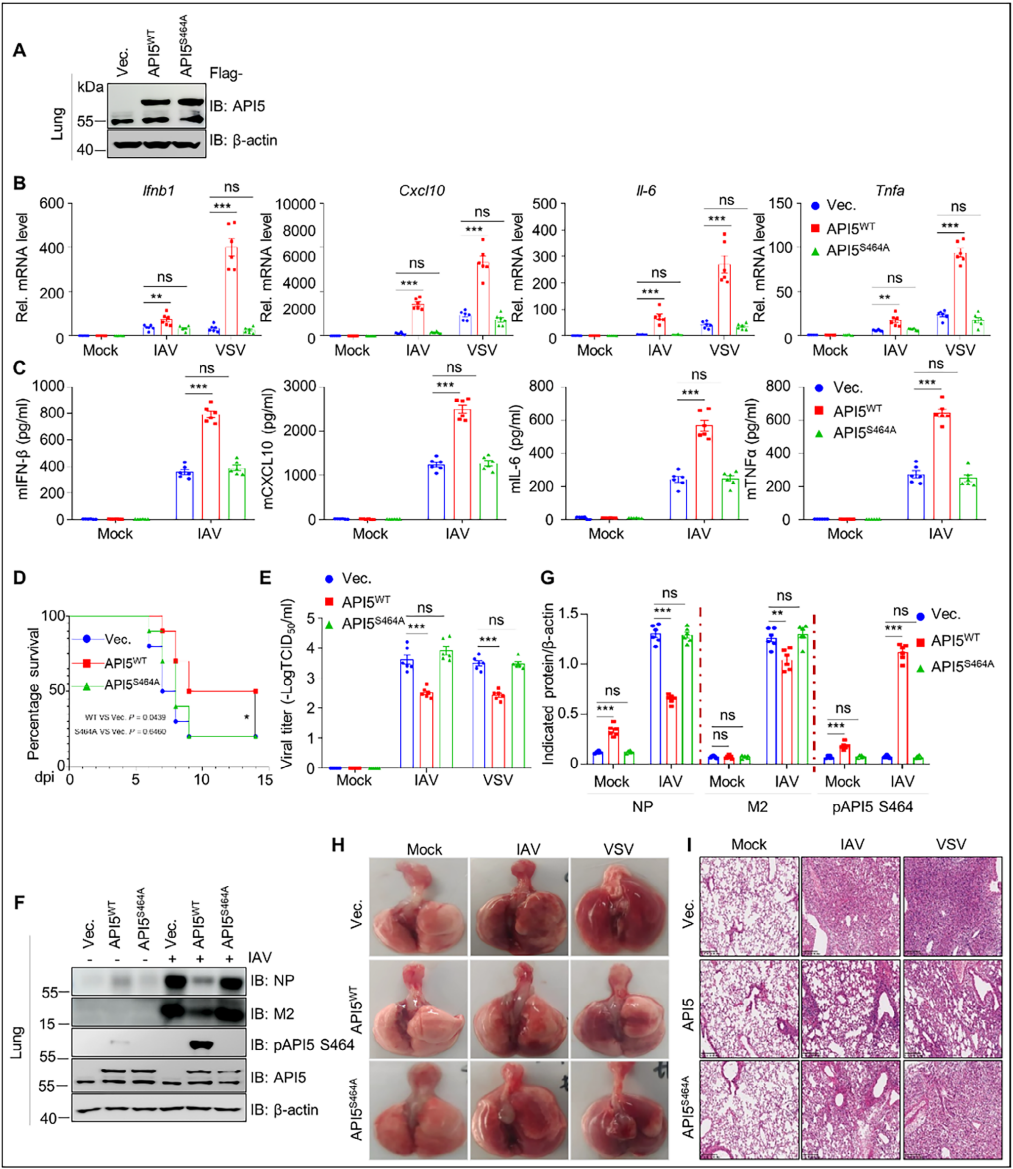
API5 drives innate immunity in mice through phosphorylation. A) Three‐week‐old C57BL/6 mice (n = 6 mice/group) were intranasally infected with AAV6 carrying Flag‐tagged API5^WT^ or API5^S464A^ (10^11^ TCID_50_ per mouse) for four weeks, and API5 expression in mouse lung was detected by immunoblotting with anti‐API5. B and C) API5^WT^‐ and API5^S464A^‐overexpressing mouse lungs (n = 6 mice/group) infected with IAV (5×10^5^ PFU mL^−1^; 50 µL per mouse) or VSV (10^8.0^ PFU mL^−1^; 60 µL per mouse) at 4 dpi were subjected to qPCR analysis for the mRNAs of *Ifnb1*, *Cxcl10*, *Il*‐*6*, and *Tnfa* (B). The BAL from IAV‐ or mock‐infected mice (n = 6 mice/group) with shCON or sh*Api5* at 4 dpi was used for ELISA assays of IFN‐β, CXCL10, IL‐6, and TNFα (C). D) API5^WT^‐ and API5^S464A^‐overexpressing mice (n = 10 mice/group) were intranasally inoculated with IAV (5×10^5^ PFU mL^−1^; 50 µL per mouse) and monitored for 14 d for survival. Kaplan‐Meier survival curves were compared using log‐rank (Mantel‐Cox) analysis. E) Virus titers of IAV‐ and VSV‐infected mouse lungs (n = 6 mice/group) with API5^WT^ and API5^S464A^ overexpression at 4 dpi were detected by TCID_50_ assays. The sample preparation is described in the method. F) Viral proteins of IAV‐infected mouse lungs (n = 6 mice/group) with API5^WT^ and API5^S464A^ overexpression at 4 dpi were immunoblotted with indicated antibodies. Data are one representative of six mice per group. G) Data in (F) were quantified and shown as the ratio of NP to β‐actin, M2 to β‐actin, and pAPI5 S464 to β‐actin. H) Gross images of IAV‐, and VSV‐infected mice lungs with API5^WT^ and API5^S464A^ overexpression at 4 dpi. The images are from representative one of six mice per group. I) HE staining of IAV‐ and VSV‐infected mouse lungs with API5^WT^ and API5^S464A^ overexpression at 4 dpi. The images are from representative one of six mice per group. Scale bar, 200 µm. Data are presented as the mean ± SEM (n = 6), and statistical significance was determined by unpaired two‐tailed Student's t‐test. (**p < 0.01; ***p < 0.001; ns, no significant).

To further ascertain the in vivo functions of pAPI5, we generated mice overexpressing API5^WT^ or phosphorylation‐deficient mutant API5^S464A^ using AAV6 transduction (**Figure** **6**A). Upon IAV or VSV infection, API5^WT^‐overexpressing mice exhibited significantly elevated mRNA and protein levels of *Ifnb1*, *Cxcl10*, *Il‐6*, and *Tnfa* in mouse lung tissues and BAL, whereas API5^S464A^ overexpression failed to induce these immune responses (Figure 6B,C). Notably, IAV‐infected mice with API5^S464A^ exhibited a significantly higher mortality rate than IAV‐infected mice with API5^WT^ (Figure 6D). Accordingly, API5^WT^‐overexpressing mouse lungs had significantly lower virus titers, reduced viral protein expression (Figure 6E–G), and milder pathology (Figure 6H,I) than controls or API5^S464A^ mice. These results confirm that pAPI5 is indispensable for antiviral innate immunity in vivo. Collectively, these data demonstrate that API5 phosphorylation at S464 is crucial for promoting antiviral immunity against RNA virus infection both in vitro and in vivo.

### 2.5 Phosphorylated API5 Blocks Autophagic Degradation of both RIG‐I and MDA5 to Prime Antiviral Immune Signaling

**Figure 7:**
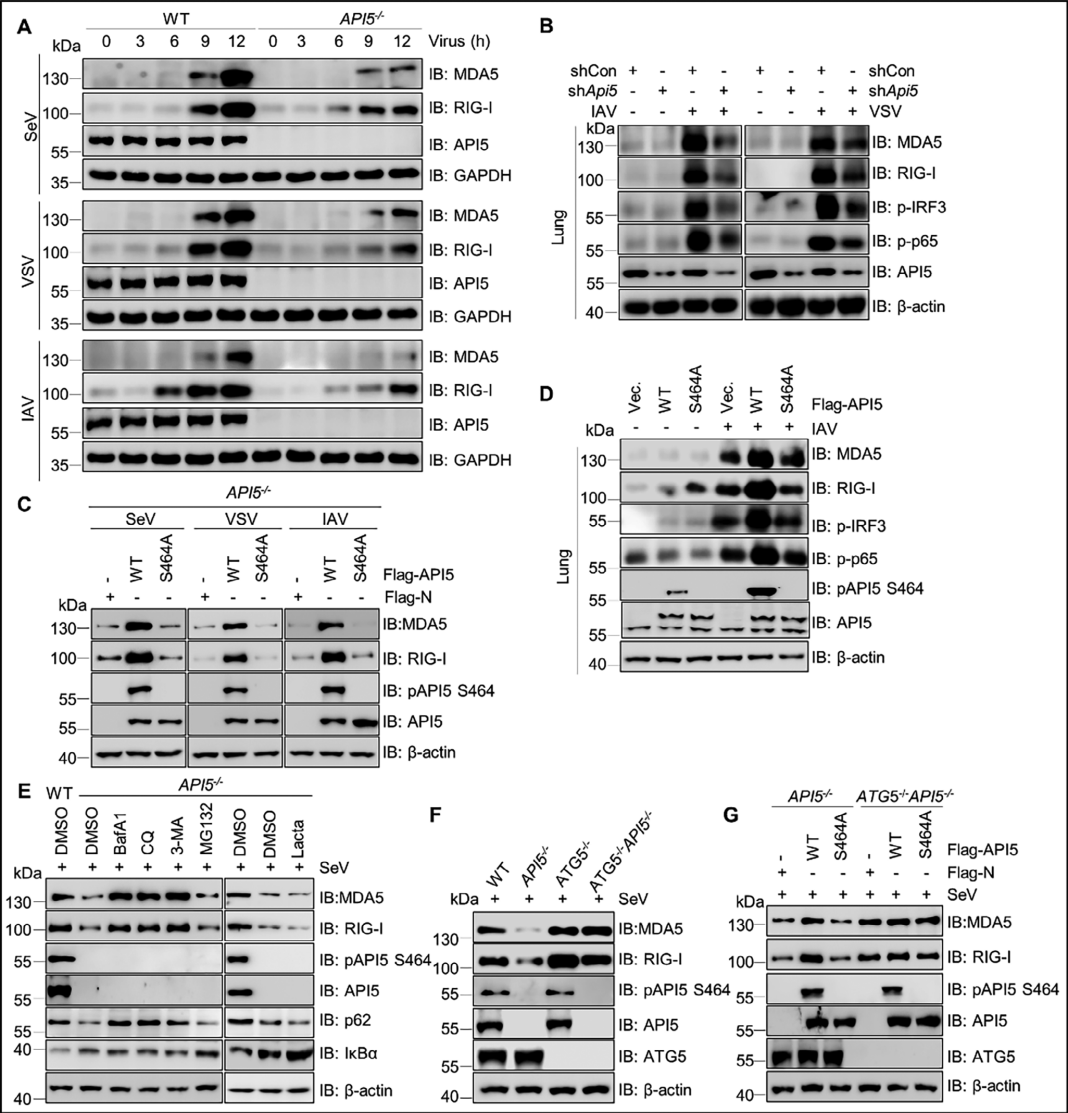
API5 inhibits autophagic degradation of RIG‐I and MDA5 by phosphorylation. A and B) Immunoblot analysis of RIG‐I and MDA5 in SeV‐, VSV‐, and IAV‐infected WT and *API5^−/−^* A549 cells (A), and lungs from shCON and sh*Api5* mice infected with IAV (5×10^5^ PFU mL^−1^; 50 µL per mouse) or VSV (10^8.0^ PFU mL^−1^; 60 µL per mouse) for 4 days (B). C and D) Immunoblot analysis of RIG‐I and MDA5 in SeV‐, VSV‐, and IAV‐infected API5^−/−^ A549 cells reintroduced with empty vector (Vec.), API5^WT^, or API5^S464A^ (C), and the lung tissues from Flag‐tagged API5^WT^‐, API5^S464A^‐ or empty vector (Vec.)‐overexpressing mice infected with IAV (5×10^5^ PFU mL^−1^; 50 µL per mouse) for 4 days (D). E) Immunoblot analysis of extracts of API5*^−/−^* A549 cells treated with bafilomycin A1 (BafA1; 200 nm), chloroquine (CQ; 50 µM), 3‐methyladenine (3‐MA; 10 mm), MG132 (10 µM) or Lactacystin (Lacta; 10 µm) for 6 h followed by infected with SeV for 12 h. (F) Immunoblot analysis of extracts of WT, *API5* ^−/−^, *ATG5* ^−/−^, *API5* ^−/−^ *ATG5* ^−/‐^ HEK293T cells infected with SeV for 12 h. G) Immunoblot analysis of extracts of WT and *ATG5* ^−/−^ HEK293T cells transfected with empty vector (Vec.) or plasmid expressing Flag‐API^5WT^, or Flag‐API5^S464A^ followed by SeV infection for 12 h. Data are one representative of three biological replicates.

The expression levels of RIG‐I and MDA5 are crucial and tightly regulated during RNA virus infection.^[^ ^21^, ^22^ ^]^ To identify if pAPI5 modulates RIG‐I and MDA5 expression to enhance innate antiviral immunity, we first detected the effects of *API5* deficit on their mRNA and protein levels in RNA virus‐infected A549 cells. Intriguingly, *API5* deficiency significantly reduced RIG‐I and MDA5 protein levels without affecting their mRNA expression (**Figure** **7**A; Figure S12A,B, Supporting Information). This phenomenon was further confirmed in IAV‐ and VSV‐infected mouse lungs with *Api5* knockdown (Figure 7B; Figure S12C, Supporting Information). These findings suggest that API5 regulates RIG‐I and MDA5 post‐transcriptionally during viral infection. Notably, the reconstitution with API5^WT^, but not the phosphorylation‐deficient mutant API5^S464A^, restored RIG‐I and MDA5 protein expression in SeV, VSV‐, and IAV‐infected *API5* ^−/‐^ A549 cells, as well as in IAV‐infected mouse lung tissues (Figure 7C,D; Figure S12D,E, Supporting Information). Taken together, these results demonstrate that pAPI5 specifically modulates the protein abundance of RIG‐I and MDA5 during viral infection.

To investigate how pAPI5 enhances RIG‐I and MDA5 protein levels, we used cycloheximide (CHX) to block mRNA translation. We found that overexpression of API5^WT^, but not API5^S464A^, effectively inhibited the degradation of RIG‐I and MDA5 (Figure S12F,G, Supporting Information). Furthermore, API5 overexpression significantly prolonged the half‐lives of both proteins, whereas the S464A mutant had no such effect (Figure S12H–K, Supporting Information). These data together suggest that API5 phosphorylation is crucial for maintaining the stability of RIG‐I and MDA5. Since autophagy and the ubiquitin‐proteasome system are two major intracellular degradative pathways.^[^ ^14^, ^17^, ^19^ ^]^ We detected which degradation system contributed to pAPI5‐prevented degradation of RIG‐I and MDA5. We observed that *API5* knockout‐mediated degradation of endogenous RIG‐I and MDA5 was blocked by autophagy inhibitor (bafilomycin A_1_ [BafA1], chloroquine [CQ], 3‐methyladenine [3‐MA]) not by proteasome inhibitor (MG132 and lactacystin [Lacta]) (Figure 7E; Figure S13A, Supporting Information), indicating that API5 inhibits the autophagic degradation of RIG‐I and MDA5.

Given that ATG5 is essential for autophagosome formation,^[^ ^58^ ^]^ we further assessed the function of API5 in *ATG*5 KO cells, in which the autophagy is impaired. In these cells, RIG‐I and MDA5 protein levels remained stable even in the absence of API5 (Figure 7F; Figure S13B, Supporting Information), confirming that autophagy is directly involved in API5‐mediated regulation of RIG‐I and MDA5 stability. Importantly, API5^WT^, but not API5^S464A^, significantly enhanced RIG‐I and MDA5 stabilization in SeV‐infected WT cells but not in *ATG5* KO cells (Figure 7G; Figure S13C, Supporting Information). Collectively, these findings suggest that API5 blocks autophagy‐mediated degradation of RIG‐I and MDA5 in a phosphorylation‐dependent manner.

**Figure 8:**
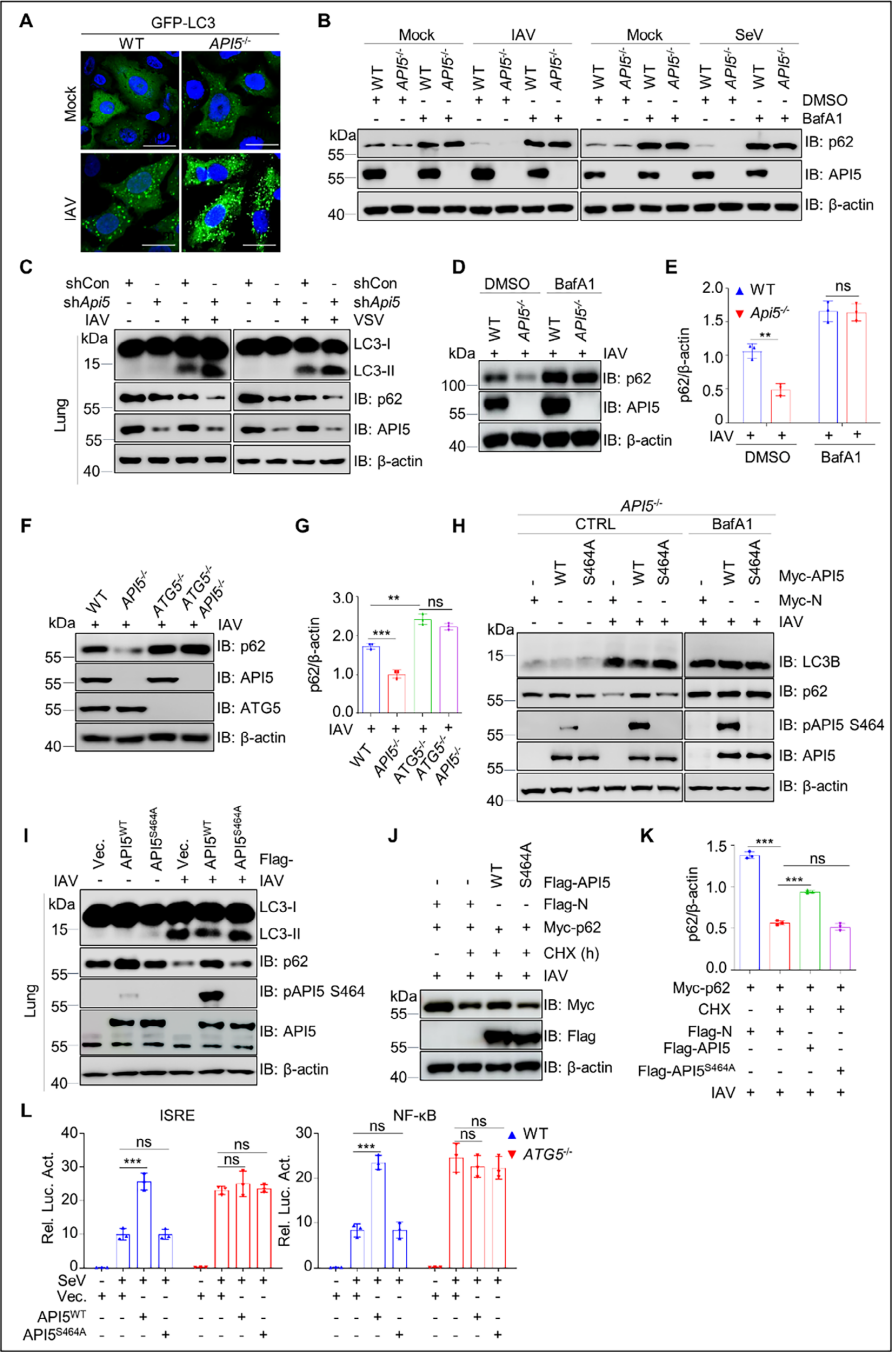
API5 inhibits autophagy to prime antiviral signaling by phosphorylation. A) Confocal analysis of GFP‐LC3 puncta in mock‐ and IAV‐infected WT and *API5* ^−/‐^ A549 cells. Scale bar, 5 µm. B) Immunoblot analysis of p62 in extracts of WT and *API5* ^−/−^ A549 cells treated with DMSO, or bafilomycin A1(BafA1; 200 nm) for 6 h followed by SeV infection for 12 h. C) Immunoblot analysis of LC3‐II and p62 in lungs (n = 6 mice/group) from shCON and sh*Api5* mice infected with IAV(5×10^5^ PFU mL^−1^; 50 µL per mouse) or VSV (10^8.0^ PFU mL^−1^; 60 µL per mouse) for 4 days. Data are one representative of six mice per group. D) WT and *API5* ^−/−^ A549 cells were infected with IAV for 12 h, followed by treatment with BafA1 (200 nm) for 6 h. Lysates were analyzed by immunoblotting. E) Data in (C) were quantified and shown as the ratio of p62 to β‐actin. F) Lysates from WT, *API5* ^−/−^, *ATG5* ^−/−^, and *ATG5* ^−/−^ *API5* ^−/−^ HEK293T cells with IAV infection for 12 h were subjected to immunoblotting with indicated antibodies. G) Data in (F) were quantified and shown as the ratio of p62 to β‐actin. H) *API5* ^−/−^ A549 cells were transfected with Myc‐tagged API5^WT^or API5^S464A^ for 24 h, followed by infection with IAV (MOI = 3) for 12 h and immunoblotting with anti‐LC3B and anti‐p62 rabbit pAbs (left). At 24 h after transfection, the cells were treated with BafA1(200 nm) for 6 h followed by IAV infection for 12 h, and then were used for the analysis of the expression levels of LC3B and p62 (right). I) Immunoblotting of LC3B and p62 proteins was conducted in lung tissues of mice (n = 6 mice/group) transfected with Flag‐tagged API5^WT^, mutant API5^S464A^, and infected with IAV (10^5.0^ PFU mL^−1^) for 4 days. Data are one representative of six mice per group. J) HEK293T cells were co‐transfected with Myc‐p62 and Flag‐tagged API5^WT^ or API5^S464A^ for 18 h, followed by IAV infection for 12 h. The resultant cells were treated with DMSO or CHX (100 µg mL^−1^) for 6 h, and immunoblotting analyzed lysates. K) Data in (J) were quantified and shown as the ratio of p62 to β‐actin. L) Luciferase activity in WT and *ATG5* ^−/−^ HEK293T cells transfected with an ISRE (left) or NF‐κB (right) luciferase reporter, Renilla luciferase plasmid, and an empty vector or plasmid encoding API5 ^WT^ or API5^S464A^ for 24 h followed by infection of SeV (25 HA units) for 12 h. For (A,B,D,F,H,J), data are one representative of three biological replicates. Data are shown as mean ± SEM (n =  3 biological replicates). Statistical significance was analyzed by unpaired two‐tailed Student's t test. (*p < 0.05; **p < 0.01; ***p < 0.001; ns, no significant).

Given the above findings, we speculated that pAPI5 is involved in the regulation of RNA virus‐induced autophagy to modulate antiviral immunity. Microtubule‐associated protein 1 light chain 3 (LC3) and p62 are key markers for monitoring autophagy.^[^ ^59^ ^]^ The conversion of LC3‐I to its lipidated form, LC3‐II (LC3B), the redistribution of LC3 from a diffuse cytoplasmic pattern to punctate structures (LC3 puncta), and p62 degradation are hallmarks of autophagy.^[^ ^60^ ^]^ To test our hypothesis, we first assessed the impact of *API5* KO on the formation of LC3 puncta. As shown in **Figure** **8**A, the number of enhanced green fluorescent protein (GFP)‐LC3 puncta was higher in IAV‐infected *API5* ^−/‐^ cells compared to WT cells. Moreover, *API5* KO reduced p62 levels in IAV‐ and SeV‐infected A549 cells, an effect reversed by BafA1 treatment (Figure 8B; Figure S14A, Supporting Information). Consistently, *API5* knockdown increased LC3B and decreased p62 levels in IAV‐ and VSV‐infected mouse lungs (Figure 8C; Figure S14B, Supporting Information). Additionally, the p62 degradation triggered by *API5* deficiency was also prevented by BafA1 treatment and *ATG5* knockout (Figure 8D–G), further supporting the role of API5 in suppressing autophagy. Interestingly, the reintroduction of API5^WT^, but not API5^S464A^, inhibited IAV‐driven autophagy in both *API5* ^−/−^ A549 cells and mouse lungs, as evidenced by decreased LC3B and increased p62 levels (Figure 8H,I; Figure S14C,D, Supporting Information). These effects were abolished upon BafA1 treatment (Figure 8H, Figure S14C, Supporting Information). Moreover, API5^WT^, but not API5^S464A^, significantly impeded p62 degradation (Figure 8J,K). Altogether, these results indicate that pAPI5 negatively regulates autophagy induced by IAV, VSV, or SeV.

Based on these findings, we hypothesized that pAPI5 enhances antiviral immunity by suppressing autophagy during RNA virus infection. First, we confirmed the involvement of the autophagy pathway in antiviral immune responses, as BafA1 treatment or *Atg5* KO increased type I interferon and pro‐inflammatory cytokine production upon SeV, VSV, or IAV infection (Figure S15A, B, Supporting Information). Notably, the enhancement of antiviral signaling by API5^WT^, but not API5^S464A^, was completely abolished in *ATG5* KO cells (Figure 8L), further supporting that pAPI5 enhances antiviral immune response through suppressing autophagy.

### 2.6 Phosphorylated API5 Eliminates p62 Ubiquitination at K141 to Impede p62‐Mediated Autophagy

**Figure 9:**
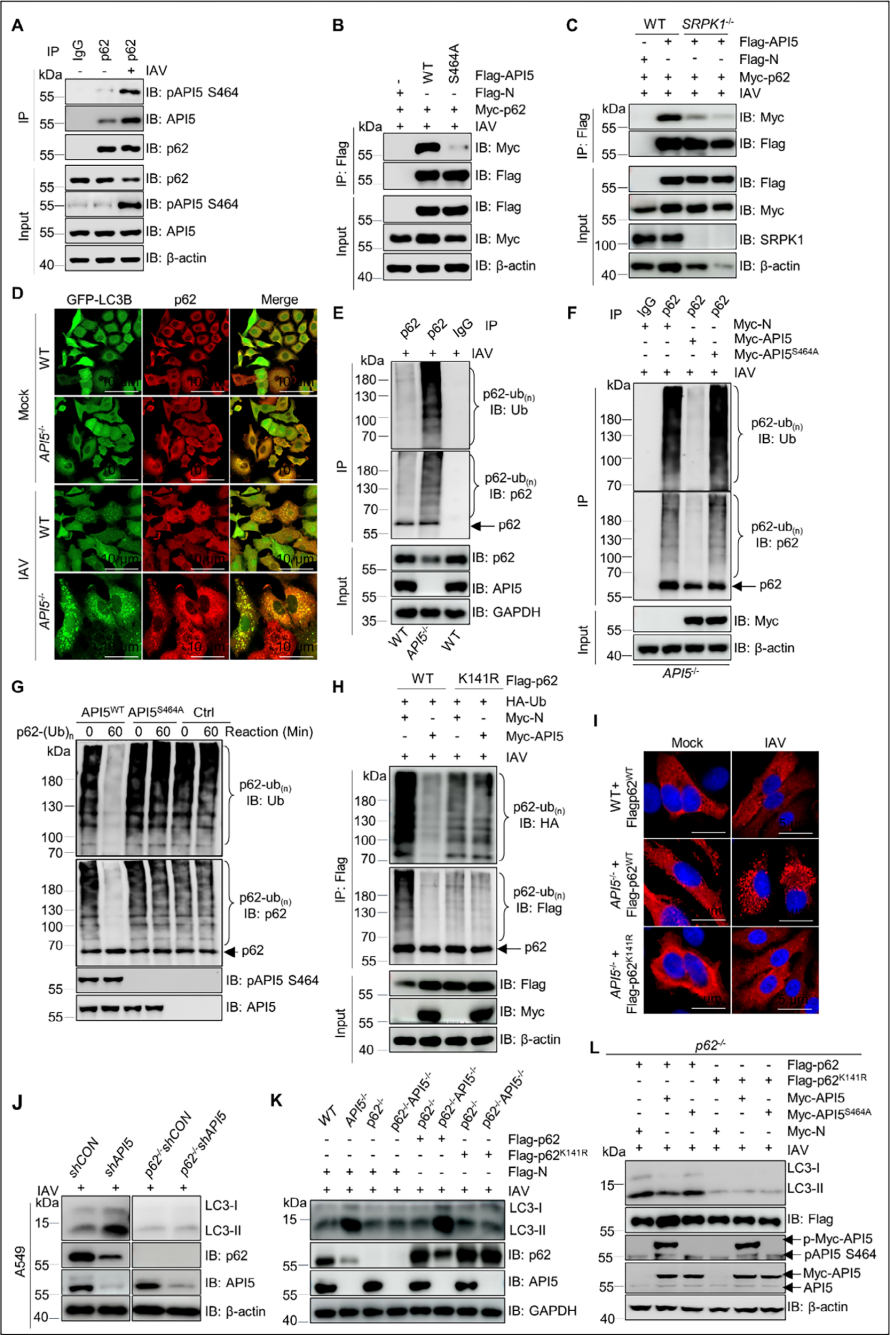
API5 deubiquitinates p62 at K141 to inhibit p62‐mediated autophagy through phosphorylation. A) A549 cells were infected with IAV or not. At 12 h after infection, the lysates were co‐immunoprecipitated with mouse p62 antibody or mouse IgG. Immuno‐precipitates were then analyzed by immunoblotting using indicated antibodies. B) Co‐ip analysis of the interaction of exogenous API5^WT^ or API5^S464A^ and p62 in IAV‐infected HEK293T cells for 12 h. C) Co‐ip analysis of the interaction of API5 with p62 in IAV‐infected WT and *SRPK1* ^−/‐^ HEK293T cells transfected with API5 and p62 plasmids followed by IAV infection for 12 h. D) Confocal analysis of the colocalization between p62 aggregations and GFP‐LC3B puncta in mock‐ and IAV‐infected WT and *API5* ^−/‐^ A549 cells. E) WT and *API5* ^−/‐^ A549 cells were infected with IAV (MOI = 3) for 12 h. p62 was immunoprecipitated from cell lysates. Immuno‐precipitates were then analyzed by immunoblotting. F) *API5* ^−/−^ A549 cells were transfected with Myc‐tagged API5^WT^ or API5^S464A^ for 24 h, followed by infection with IAV for 12 h. p62 was immunoprecipitated from cell lysates, and immune‐precipitates were analyzed by immunoblotting. G) Recombinant His‐Flag‐p62 was incubated with in vitro ubiquitination assay system containing E1(UBA1), E2 (UBE2D2/UBE2D3), TRIM21, and Ub, followed by purification using anti‐FLAG M2 Agarose Affinity. Ubiquitinated His‐Flag‐p62 was incubated with recombinant Flag‐tagged API5^WT^ or mutant API5^S464A^ from HEK293T cells for 60 min, followed by immunoblotting using anti‐Ubiquitin mAb and anti‐p62 rabbit pAb. H) HEK293T cells were co‐transfected with Flag‐tagged p62^WT^ or p62^K141R^, HA‐Ub, and Myc‐API5 for 24 h, followed by IAV infection for 12 h. Lysates were then subjected to immunoprecipitation and immunoblotting. I) *p62* ^−/‐^ and *p62* ^−/−^ *API5* ^−/‐^ HEK293T cells were transfected with Flag‐tagged p62^WT^ or p62^K141R^ for 30 h, followed by infection with IAV for 12 h. The resultant cells were analyzed with anti‐Flag antibody (Red), cellular nuclei were stained with DAPI (Blue). J) Lysates from shCON, sh*API5*, *p62* ^−/‐^ shCON and *p62* ^−/−^sh*API5* A549 cells with IAV infection for 12 h were analyzed by immunoblotting. K) *p62* ^−/‐^ and *p62* ^−/−^ *API5* ^−/‐^ HEK293T cells were transfected with Flag‐tagged p62^WT^ or p62^K141R^ for 30 h, followed by infection with IAV for 12 h. Lysates were then immunoblotted using indicated antibodies. L) *p62* ^−/−^ HEK293T cells were co‐transfected with Flag‐p62 or Flag‐p62 ^K141R^ and Myc‐API5 or Myc‐API5 ^S464A^ for 30 h, followed by stimulation with IAV for 12 h. Lysates were then analyzed by immunoblotting using indicated antibodies. Data are one representative of three biological replicates.

In addition to serving as an indicator of autophagic flux, p62 plays a pivotal role as a key regulator of selective autophagy and negatively regulates the antiviral signaling pathway.^[^ ^14^, ^17^, ^21^, ^61^, ^62^ ^]^ To investigate if pAPI5 regulates p62‐mediated autophagy to promote antiviral signaling, we first analyzed their interaction. Co‐IP assays demonstrated that pAPI5 strongly binds p62 specifically during IAV infection (**Figures** **9**A, S16A, Supporting Information). In contrast, the API5^S464A^ mutant showed weaker binding compared to API5^WT^ under identical conditions (Figure 9B, Figure S16B, Supporting Information). Importantly, this interaction was notably decreased in *SRPK1* KO cells even after IAV infection (Figure 9C, Figure S16C, Supporting Information). suggesting that phosphorylation of API5 at S464 contributes to the association with p62.

Given that p62 aggregation, a process dependent on ubiquitination, is crucial for autophagy induction,^[^ ^35^, ^61^ ^]^ we investigated pAPI5's role in this process. Confocal imaging showed that *API5* KO significantly promoted the formation of p62 aggregates and the colocalization with the autophagy marker LC3 in IAV‐infected A549 cells (Figure 9D), implicating API5 in p62‐autophagy regulation. Strikingly, the restoration of API5^WT^, but not the phosphorylation‐deficient mutant API5^S464A^, significantly reduced the pronounced p62 aggregates in IAV‐infected *API5* ^−/−^ A549 cells (Figure S17A, Supporting Information), strongly implicating API5 phosphorylation in this regulatory mechanism. Consistent with this, p62 immunoprecipitates from IAV‐infected *API5* ^−/−^ cells exhibited elevated ubiquitination, which was reduced by API5^WT^ but not the S464A mutant (Figure 9E,F). Collectively, these findings demonstrate that pAPI5 at S464 inhibits p62 ubiquitination, thereby preventing p62 aggregate formation and subsequent autophagy activation.

Considering the facts that ubiquitin association (UBA) domain of p62 is prone to bind with ubiquitinated proteins and its N‐terminal Phox1 and Bem1p (PB1) domain mediates self‐oligomerization, p62‐immunoprecipitiated complex may contain a bulk of ubiquitinated proteins.^[^ ^35^ ^]^ To exclude the possibility that API5 reduces ubiquitinated species by deubiquitinating these associated proteins, we generated a truncation mutant p62△UBA and ubiquitinated p62 in vitro as described previously.^[^ ^35^ ^]^ Results showed that API5 overexpression reduced the levels of ubiquitinated species of transfected p62 and p62△UBA in *p62* ^−/‐^ HEK293T cells (Figure S17B, C, Supporting Information). Moreover, purified API5^WT^ from transfected HEK293T cells, but not mutant API5^S464A^, decreased ubiquitination of ubiquitinated p62 generated in vitro (Figure 9G; Figure S17D, Supporting Information). Collectively, these findings indicate that pAPI5 inhibits p62 ubiquitination.

Since p62 functionality depends on its ubiquitination sites,^[^ ^31^, ^34^ ^]^ we systematically substituted every K residue with arginine (R) within p62 (Figure S18A, Supporting Information). Notably, API5 overexpression failed to reduce ubiquitination of the p62^K141R^ mutant (Figure 9H; Figure S18B, Supporting Information). Intriguingly, *API5* KO markedly facilitated the aggregate formation of the p62^WT^ but not p62^K141R^ following IAV infection (Figure 9I), highlighting K141 as the critical ubiquitination site regulated by API5. To further investigate the dependence of pAPI5‐regulated autophagy on p62, we generated *p62* ^−/−^sh*API5* and p*62* ^−/−^shCON (scramble control) A549 cells, as well as *API5* ^−/‐^ and *p62* ^−/−^ *API5* ^−/−^ HEK293T cells (Figure S18C‐G, Supporting Information). Results showed that *API5* deficiency failed to enhance IAV‐induced LC3B expression in *p62* ^−/−^ cells (Figure 9J; Figure S19A, Supporting Information), reinforcing the role of API5 in p62‐mediated autophagy. Remarkably, transfection of p62^WT^, but not p62^K141R^, into p62^−/−^ *API5* ^−/‐^ cells increased LC3B and decreased p62 levels (Figure 9K; Figure S19B, Supporting Information), suggesting that API5 suppresses autophagy by inhibiting K141 ubiquitination. Finally, co‐transfection of API5^WT^ and p62^WT^, but not API5^S464A^ and p62^K141R^, decreased LC3B and increased p62 in *p62* ^−/‐^ cells (Figure 9L; Figure S19C, Supporting Information). Collectively, these results indicate that pAPI5 inhibits p62‐mediated autophagy by specifically blocking ubiquitination at K141.

### 2.7 The SRPK1‐API5‐p62 Axis Is Essential for Innate Antiviral Immunity

**Figure 10:**
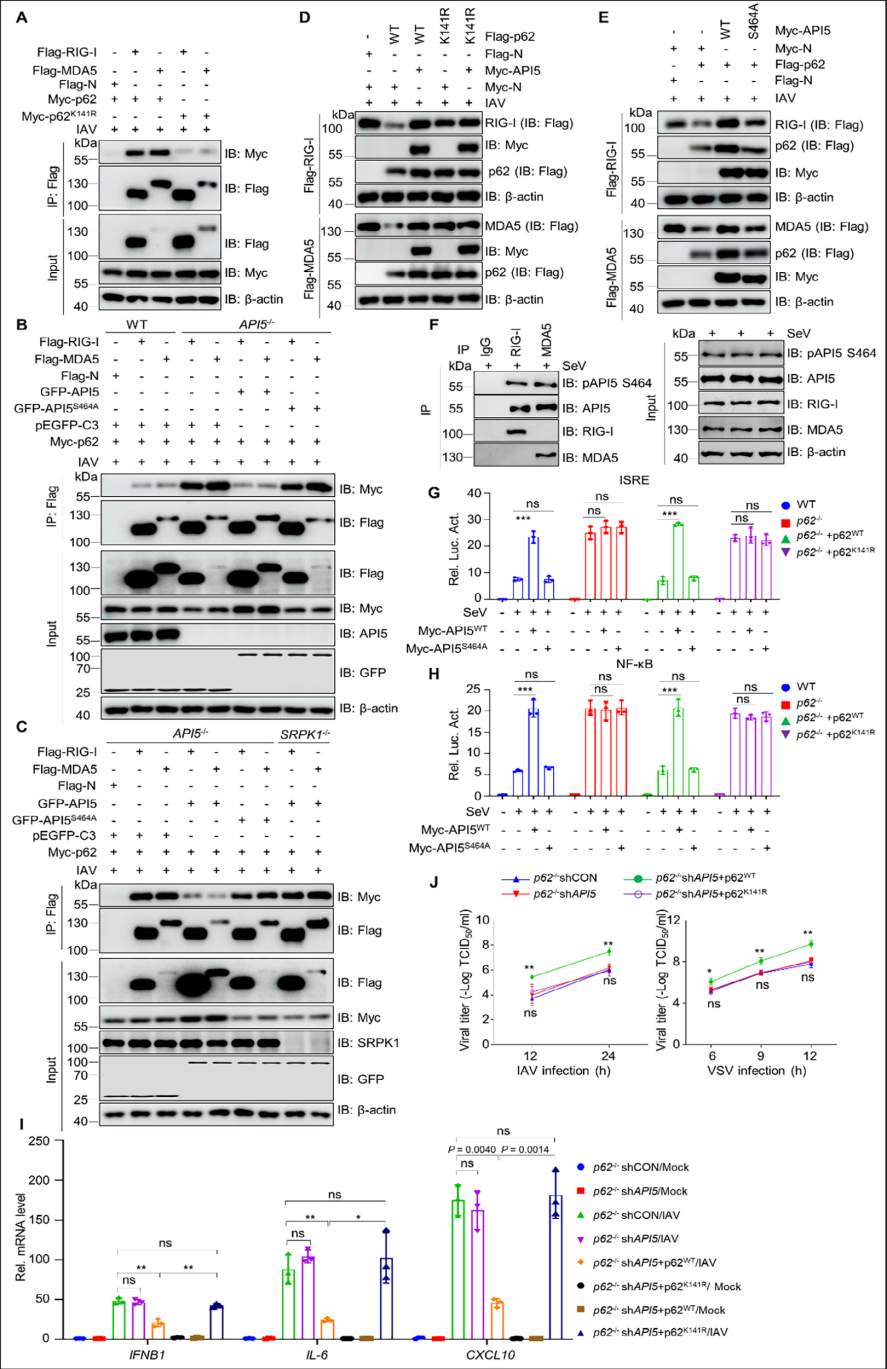
Phosphorylation of API5 is essential for priming antiviral responses through inhibiting ubiquitin‐p62‐mediated autophagic degradation of RIG‐I/MDA5. A) Flag‐RIG‐I or Flag‐MDA5 and Myc‐p62 or Myc‐p62 ^K141R^ were co‐transfected into HEK293T cells for 24 h, followed by IAV (MOI = 3) for 12 h. The lysates were subjected to immunoprecipitation and immunoblotting. B) Flag‐RIG‐I, Flag‐MDA5, and Myc‐p62, along with GFP‐API5 or GFP‐API5^S464A^, were separately co‐transfected into WT and *API5* ^−/‐^ HEK293T cells for 24 h, followed by IAV infection for 12 h. The lysates were used for immunoprecipitation and immunoblotting. C) Flag‐RIG‐I, Flag‐MDA5 and Myc‐p62, along with GFP‐API5 or GFP‐API5^S464A^, were separately co‐transfected into *API5* ^−/−^ and *SRPK1* ^−/‐^ HEK293T cells for 24 h, followed by IAV infection for 12 h. The lysates were used for immunoprecipitation and immunoblotting. D) Flag‐RIG‐I or Flag‐MDA5, Flag‐p62 or Flag‐p62^K141R,^ and Myc‐API5 were co‐transfected into HEK293T cells for 24 h, followed by IAV (MOI = 3) infection for 12 h. The lysates were analyzed by immunoblotting. E) Flag‐RIG‐I or Flag‐MDA5, Flag‐p62, and Myc‐API5 or Myc‐API5^S464A^ were separately co‐transfected into cells for 24 h, followed by IAV infection for 12 h. The lysates were analyzed by immunoblotting. F) A549 cells were infected with SeV (25 HA units) or not. At 12 h after infection, the lysates were co‐immunoprecipitated with rabbit RIG‐I, MDA5 antibody, or rabbit IgG. Immuno‐precipitates were then analyzed by immunoblotting using indicated antibodies. G and H) Luciferase activity in WT and *p62* ^−/−^ HEK293T cells transfected with an ISRE (G) or NF‐κB (H) luciferase reporter, Renilla luciferase plasmid, p62^WT^ or p62^K141R^ plasmid, and an empty vector or plasmid encoding API5 ^WT^ or API5^S464A^ for 24 h followed by infection of SeV (25 HA units) for 12 h. I) *p62* ^−/−^shCON and *p62* ^−/−^sh*API5* A549 cells were transfected with Flag‐tagged p62^WT^ or p62^K141R^ for 36 h, followed by infection with IAV for 12 h. The cells were harvested for quantitative PCR assays. J) *p62* ^−/−^shCON and *p62* ^−/−^sh*API5* A549 cells were transfected with Flag‐tagged p62^WT^ or p62^K141R^ for 24 h, followed by infection with IAV (MOI = 3) (left), and VSV (MOI = 0.1) (right). At the indicated time, the supernatants were collected for virus titer detection. For A‐F, data are one representative of three biological replicates. Data were shown as mean ± SEM (n = 3 biological replicates), and statistical significance was analyzed by unpaired two‐tailed Student's t test. (*p < 0.05; **p < 0.01; ***p < 0.001; ns, no significant).

Given that p62 binds to both RIG‐I and MDA5 to facilitates their autophagic degradation,^[^ ^21^, ^22^ ^]^ and considering our findings that ubiquitination of p62 at K141 regulated by pAPI5 is crucial for its autophagic induction (Figure 9H–L), we speculated that pAPI5 suppresses p62 ubiquitination at K141 to block the autophagic degradation of RIG‐I and MDA5, thereby enhancing innate antiviral immunity. To test this, we first assessed the role of K141 in the interaction between p62 and RIG‐I/MDA5 during IAV infection. Co‐IP assays revealed that p62 interacted with RIG‐I and MDA5, with K141 playing a crucial role in mediating this association (**Figure** **10**A; Figure S20A, Supporting Information). Furthermore, since API5 reduces p62 ubiquitination at K141 through SRPK1‐mediated phosphorylation at S464 (Figure 9F–H), we investigated the impacts of *API5* KO, *SRPK1* KO, and reconstitution of WT or S464A mutant API5 on p62 interaction with RIG‐I and MDA5. Results showed that *API5* KO or *SRPK1* KO increased p62's interaction with RIG‐I and MDA5, while restoration of API5^WT^, but not mutant API5^S464A^, reversed this effect (Figure 10B,C; Figure S20B,C, Supporting Information). These results collectively demonstrate that pAPI5 disrupts p62's association with RIG‐I and MDA5 by reducing K141 ubiquitination.

Next, to determine whether pAPI5‐mediated inhibition of p62 K141 ubiquitination is crucial for preventing the autophagic degradation of RIG‐I and MDA5, we compared the effects of API5^WT^ and API5^S464A^ on p62^WT^‐, and p62^K141R^‐mediated degradation of RIG‐I and MDA5. The results showed that API5 only blocked the autophagic degradation of RIG‐I and MDA5 mediated by p62^WT^, not by the ubiquitination‐deficient p62^K141R^ mutant. Moreover, API5^WT^, but not API5^S464A^, inhibited p62‐dependent degradation of RIG‐I and MDA5 (Figure 10D,E; Figure S20D,E, Supporting Information). These findings confirm that pAPI5 inhibits RIG‐I/MDA5 autophagic degradation by suppressing p62 ubiquitination at K141. Given that API5 binds RIG‐I and MDA5 (Figure 3C), we speculated that this interaction involves the phosphorylated form of API5. Indeed, co‐IP assays revealed that pAPI5 interacted with RIG‐I and MDA5 in SeV‐infected cells (Figure 10F). Furthermore, in IAV‐infected wild‐type cells, API5^WT^, but not the mutant API5^S464A^, bound RIG‐I and MDA5, whereas this interaction was lost in *SRPK1* KO cells (Figure S21A,B, Supporting Information), indicating that S464 phosphorylation is required for API5's association with RIG‐I and MDA5. Functional studies revealed that API5^WT^’s ability to enhance ISRE and NF‐κB activity, as well as antiviral signaling, was nearly completely abolished in *p62* KO cells. Reintroduction of p62^WT^, but not p62^K141R^ restored this effect (Figure 10G–I), underscoring the importance of pAPI5‐mediated inhibition of p62 K141 ubiquitination in facilitating antiviral innate immunity. Consistent with this, *API5* deficiency enhanced IAV and VSV replication in *p62* ^−/‐^ cells reintroduced with p62^WT^, but not with p62^K141R^ (Figure 10J). Collectively, our findings identify that SRPK1‐mediated phosphorylation of API5 at S464 is essential for blocking p62 K141ubiquitination, thereby preventing autophagic degradation of RIG‐I and MDA5, promoting innate antiviral signaling, and defending RNA virus infection.

## 3 Discussion

RIG‐I‐like receptors RIG‐I and MDA5 serve as crucial viral RNA sensors that initiate innate antiviral immune responses.^[^ ^5^ ^]^ Accumulating evidence has established the selective autophagy receptor p62 as a key mediator in the degradation of RLRs.^[^ ^16^, ^21^, ^23^, ^63^ ^]^ Notably, ubiquitination has emerged as a critical regulator of p62 activity.^[^ ^35^ ^]^ Ubiquitination not only serves as a recognition signal for p62‐mediated selective autophagy but also directly modulates p62's oligomerization state and affinity for its substrates.^[^ ^14^, ^21^, ^31^ ^]^ Although LRRC59 has been identified as a negative regulator of p62‐mediated RIG‐I autophagic degradation that promotes type I IFN signaling,^[^ ^22^ ^]^ the broader regulatory landscape, particularly through ubiquitin‐dependent mechanisms, remains unexplored. Our study reveals a novel regulatory axis in which pAPI5 suppresses p62 activity to stabilize RIG‐I and MDA5 by inhibiting p62 ubiquitination at K141, thereby enhancing antiviral immunity. Intriguingly, this finding contrasts with a recent report demonstrating that OTUD7B‐mediated deubiquitination of p62 at K7 enhances its function, leading to IRF3 degradation and subsequent attenuation of antiviral immunity.^[^ ^14^ ^]^ These opposing effects further provide compelling evidence that ubiquitin‐dependent regulation of p62 activity is strictly site‐specific, with distinct modifications dictating divergent functional outcomes.^[^ ^35^ ^]^ Our findings provide critical insights into the intricate post‐translational regulation of RIG‐I and MDA5 stability, revealing how site‐specific ubiquitination of p62 fine‐tunes antiviral immunity. By identifying pAPI5 as a key stabilizer of RLRs, we not only expand the understanding of host defense mechanisms but also highlight potential therapeutic targets for modulating immune responses against RNA viruses.

API5 was initially identified as a nuclear protein.^[^ ^36^ ^]^ Recently, our research revealed that *Avibirnavirus* infection induces de‐SUMOylation of chicken API5 in the nucleus.^[^ ^43^, ^64^ ^]^ However, thus far, the function of cytoplasmic API5 remains unexplored. In this study, we elucidated that the residue S464 of API5 serves as the phosphorylation site, and pAPI5 is located in the cytoplasm. Although previous studies have reported that API5 is involved in antiviral responses,^[^ ^41^, ^42^, ^43^ ^]^ its mechanistic role in antiviral innate immunity remains unexplored. Our study uncovers a previously unrecognized mechanism of antiviral defense by demonstrating that various RNA virus infection (e.g., IAV, SeV, or VSV) enhances SRPK1‐dependent phosphorylation of API5 at S464. This post‐translational modification is essential for API5 to suppress p62‐mediated autophagic degradation of RIG‐I and MDA5, thereby amplifying antiviral innate immune response. Our findings identify API5 phosphorylation as a critical regulatory node that links autophagy inhibition to enhance antiviral immunity, providing novel therapeutic strategies to bolster host defense against RNA viruses and a potential immunomodulator.

SRPK1, identified primarily in the cytoplasm, is the first serine‐arginine (SR) protein kinase to be cloned^[^ ^65^ ^]^ and an auto‐phosphorylated kinase by forming a complex with AKT2 and then trans‐locates into the nucleus to trigger hyperphosphorylation of SR‐rich proteins.^[^ ^66^ ^]^ Hyperphosphorylated SR proteins are then recruited to the transcription and splicing machinery to regulate gene expression and pre‐mRNA splicing.^[^ ^67^ ^]^ Interestingly, existing studies demonstrate that DNA viruses such as herpes simplex virus 1 (HSV‐1) and human papillomavirus type 16 (HPV16) differentially regulate SRPK1 activity.^[^ ^68^, ^69^ ^]^ HSV‐1 utilizes its viral protein ICP27 to suppress SRPK1 function,^[^ ^69^ ^]^ whereas HPV16 conversely enhances SRPK1 activity through viral protein E2‐mediated upregulation.^[^ ^68^ ^]^ However, whether RNA viruses modulate SRPK1 remains unexplored. In the present study, we identified API5 as a novel SRPK1 substrate and showed that diverse RNA virus infection robustly increases cytoplasmic API5 phosphorylation. Given SRPK1's well‐established role in RNA splicing,^[^ ^67^, ^70^ ^]^ and our observation that multiple RNA viruses enhance SRPK1‐mediated API5 phosphorylation (Figure 4B–E), we speculate that SRPK1 activation may result from direct recognition of viral RNA. However, this hypothesis warrants further validation.

Although SRPK1 implicated in RIG‐I‐mediated type I interferon gene expression,^[^ ^71^ ^]^ the underlying mechanism remains elusive. In this study, we revealed that SRPK1 phosphorylates API5 at Ser464, which potentiates RLR antiviral signaling by suppressing p62‐dependent autophagic degradation of RIG‐I and MDA5. Given the established roles of SRPK1 in RNA splicing and transcription^[^ ^67^, ^70^, ^72^, ^73^ ^]^ and the RNA binding function of API5,^[^ ^74^ ^]^ we propose the following scientific hypothesis: SRPK1‐mediated API5 phosphorylation may also modulate host or viral RNA splicing and transcription in addition to regulating antiviral immunity, thereby combating viral infection. These intriguing hypothetical possibilities warrant further investigation in future studies.

Ubiquitination constitutes a critical biochemical process governing p62‐mediated autophagy.^[^ ^14^ ^]^ Ubiquitination sites determines p62 activity. For example, ubiquitination at K7 inhibits autophagy by enhancing p62 dimerization,^[^ ^31^ ^]^ whereas ubiquitination at K420 accelerates autophagy by impeding p62 dimerization.^[^ ^34^ ^]^ Additionally, ubiquitination of p62 at residues K91 and K189 has been shown to promote autophagy.^[^ ^32^ ^]^ Here, we identified a previously undiscovered ubiquitination site at K141 (Figure 9H and Figure S18B, Supporting Information) and demonstrate that K141 ubiquitination promotes IAV‐induced p62‐dependent autophagy (Figure 9K,L). Mechanistic studies further revealed that K141 ubiquitination facilitated p62 aggregate formation (Figure 9I), which is consistent with p62 aggregates‐mediated autophagy induction.^[^ ^75^, ^76^, ^77^ ^]^

Notably, *API5* knockout significantly enhanced IAV‐induced p62 aggregate formation and autophagy (Figure 9D–K), while reconstitution with wild‐type API5, but not the phosphorylation‐deficient mutant S464A, markedly suppressed this process (Figure 9L and Figure S17A, Supporting Information). Mechanistically, pAPI5 inhibits p62 ubiquitination at K141 during IAV infection, thereby reducing aggregate formation and autophagy induction. Intriguingly, pAPI5 functions similarly to the p62 deubiquitinase USP8. However, unlike USP8, which specifically targets p62 ubiquitination at K420,^[^ ^35^ ^]^ phosphorylated API5 selectively inhibits K141 ubiquitination. To date, there have been no reports demonstrating that API5 possesses deubiquitinase (DUB) activity. However, our unpublished mass spectrometry data of API5 immunoprecipitation complexes revealed potential interactions between API5 and multiple USP family proteins. The mechanism by which pAPI5 inhibits p62 ubiquitination, whether it occurs through recruitment of USPs or by functioning as a novel ubiquitin‐specific peptidase itself, remains to be elucidated in future studies.

In conclusion, our study identifies pAPI5 as a novel regulator of RLR‐mediated innate antiviral signaling and p62 ubiquitination‐dependent autophagy. Importantly, we elucidate how SRPK1‐API5 phosphorylation axis disarms ubiquitin‐p62 autophagic checkpoint to license RLR antiviral surveillance. Mechanistic interrogation reveals that diverse RNA viruses enhance SRPK1‐mediated API5 phosphorylation at S464. The p API5 targets and deubiquitinates p62 at K141, thereby attenuating p62‐mediated autophagy. This process effectively hinders the autophagic degradation of RIG‐I and MDA5, amplifying innate antiviral immunity and fortifying the host defense against RNA viral infection (Figure S22, Supporting Information).

## 4 Experimental Section

### Cell Lines

Human differentiated macrophage THP‐1(SCSP‐567), human lung epithelial A549 (CRM‐CCL‐185), and human embryonic kidneys (HEK) 293T cells (CRL‐11268) were obtained from the American Type Culture Collection (ATCC). Wild‐type (WT) and *Atg5* ^−/‐^ mouse embryonic fibroblasts (MEF) cells were the kind gift of Professor Wei Liu from Zhejiang University. *p62* ^−/−^ and *ATG5* ^−/−^ HEK293T cells were stored in the laboratory and had been previously described.^[^ ^17^ ^]^ All cell lines, except THP‐1, were cultured in Dulbecco's modified Eagle's medium (DMEM; 12100‐038, Gibco) supplemented with 10% (v/v) fetal bovine serum (FBS; 1616756, Biological Industries, Israel) and 1‰ (v/v) penicillin‐streptomycin (P0781, Sigma‐Aldrich) at 37 °C in 5% CO2 (v/v). THP‐1 cells were maintained in RPMI 1640 medium (31800105, Gibco) under the same incubation conditions.

### Viruses

eGFP‐tagged Vesicular Stomatitis Virus (VSV) was generously provided by Prof. Pinglong Xu (Zhejiang University, Hangzhou), and Sendai Virus (SeV) was gifted from Prof. Jin Jin (Zhejiang University, Hangzhou). A/Puerto Rico/8/34 (PR8, H1N1) was stored in the laboratory. The following adeno‐associated virus 6 (AAV6) were custom‐produced by WZ Biosciences Inc. (Shandong, China): AAV6‐sh*Api5* (*Api5*‐targeting shRNA, 3.27 × 10¹^3^ TCID₅₀ [50% tissue culture infective dose] mL^−1^), AAV6‐shCON (scrambled shRNA control, 1.67 × 10¹^3^ TCID₅₀ mL^−1^), AAV6‐Flag‐API5 (human Flag‐tagged API5, 2.27 × 10¹^3^ TCID₅₀ mL^−1^), AAV6‐Flag‐API5^S464A^ (human Flag‐tagged mutant API5, 2.42 × 10¹^3^ TCID₅₀ mL^−1^), and AAV6‐empty vector (1.09 × 10¹^3^ TCID₅₀ mL^−1^). SeV and PR8 viruses were produced by inoculating the virus into the allantoic cavity of 9 to 11‐day‐old embryonated specific‐pathogen‐free chicken eggs. eGFP‐tagged VSV was produced in baby hamster syrian kidney (BHK)‐21 cells. All the viruses were stocked at −80 °C.

### Antibodies and Reagents

The following antibodies were used in this study: Rabbit anti‐Flag Tag (0912), Rabbit anti‐His Tag (0812), Mouse anti‐GST Tag (EM80701), Mouse anti‐β‐Actin (M1210), Mouse anti‐Histone H3 (M1306), Rabbit anti‐IKKα/β (ET1611‐23), Rabbit anti‐p65 (ET1603‐12), and Rabbit anti‐ATG5 (ET1611‐38) were purchased by HuaAn Biotechnology; Mouse anti‐Flag Tag (F1804) was from Sigma–Aldrich; Rabbit anti‐GAPDH (AB‐P‐R001) was from GoodHere Technology; Mouse anti‐influenza viral proteins were prepared and stored in the laboratory; Rabbit anti‐API5 (ab65836) and Rabbit anti‐p62 (ab109012) were from Abcam; Mouse anti‐API5 (sc‐393341) and Mouse anti‐RIG‐I (sc‐376845) were from Santa Cruz; Rabbit anti‐SRPK1(A5854) and Rabbit anti‐MDA5 (A13645) were from ABclonal; Rabbit anti‐ RIG‐I (67556‐1‐Ig) and Rabbit anti‐IRF3 (11312‐1‐AP) were from Proteintech; Rabbit anti‐phospho‐IRF3 (13 786) was from Signalway Antibody; Rabbit anti‐MDA5 (5321), Mouse anti‐Ubiquitin (3936), Rabbit anti‐TBK1(3504), Rabbit anti‐phospho‐TBK1 (5483), Rabbit anti‐phospho‐IKKα/β (2697), Rabbit anti‐phospho‐p65 (3033), Rabbit anti‐phospho‐IκBα (2859) and Rabbit anti‐LC3B (2775) were purchased by Cell Signaling Technology; HRP‐conjugated goat‐anti mouse IgG (074‐1806) and HRP‐conjugated goat‐anti rabbit IgG (074‐1506) were from Kirkegaard & Perry Laboratories, Inc. Bafilomycin A1(BafA1; HY‐100558), chloroquine (CQ; HY‐17589A), 3‐methyladenine (3‐MA; HY‐19312), MG132 (HY‐13259), Lactacystin (Lacta; HY‐16594), SRPIN340 (HY‐13949,) and cycloheximide (CHX; HY‐12320) were purchased from MedChemExpress. MG132 was used as previously described.^[^ ^14^, ^22^ ^]^ Poly (I:C)‐LMW (tlrl‐picw), poly (I:C)‐HMW (tlrl‐pic) and puromycin (58‐58‐2) were provided by Invivogen. N‐ethylmaleimide (NEM; E3876), β‐glycerophosphate disodium salt hydrate (G9422), and Anti‐FLAG M2 Affinity Gel (A2220) were offered by Sigma–Aldrich. 1,4‐dithio‐DL‐threitol (DTT; 3483‐12‐3) was from Solarbio. Adenosine 5′‐triphosphate (ATP; D7378‐1 mL), 3× Flag Peptide (P9801), Dual‐luciferase reporter assay kit (RG027), Mouse control IgG (A7028), and Rabbit control IgG (A7016) were from Beyotime Biotechnology. Protein A/G PLUS‐Agarose (sc‐2003) was from Santa Cruz Biotechnology. GST resin (16 100) was from Thermo Fisher. Ni‐NTA agarose (30 210) was from QIAGEN. Reduced glutathione (A100399‐0005) was from Sangon Biotech. Mouse IFN‐β ELISA Kit (RK00420), Mouse CXCL10/IP10 ELISA Kit (RK00056), Mouse IL‐6 ELISA Kit (RK00008), Mouse TNF‐α ELISA Kit (RK00027), Human IFN‐β ELISA Kit (RK00030), Human CXCL10/IP10 ELISA Kit (RK00054) and Human IL‐6 ELISA Kit (RK00004) were offered by ABclonal. All detailed information is summarized in Table S3 (Supporting Information).

### Generation of API5 pS464 Antibody

Rabbit polyclonal antibody recognizing phosphorylated API5 pS464 was customized from ABclonal (Wuhan, China). Peptides containing API5 pS464 were injected into rabbits for four rounds. The rabbit serum was collected and purified using an affinity column conjugated with nonmodified peptide to exclude antibodies recognizing non‐modified API5, followed by an affinity column conjugated with API5 pS464 peptide to bind to and purify the antibodies. Specific antibodies were then eluted and concentrated.

### Construction of Plasmids and Transfection

DNA fragments encoding full‐length gene were amplified from HEK293T cells and individually subcloned into the following expression vectors: pCMV‐Myc‐N (635 689, Clontech), pCMV‐Flag‐N (635 689, Clontech), pEGFP‐C3 (6082‐1, Clontech), mCherry‐C1 (632 524, Clontech), pET‐28a‐C (69864‐3, Novagen), and pGEX‐4T‐1 (27‐4580‐01, GE Healthcare Biosciences). Mutations in Flag‐, Myc‐, mCherry‐, and eGFP‐tagged API5, Flag‐p62, and pET‐28a‐SRPK1 were generated using a site‐directed mutagenesis method, with the wild‐type plasmid as the template. Primer sequences used for cloning are provided upon request. All constructs used in this study are listed in Table S1 (Supporting Information) and available upon request. All constructs were validated by sequencing and were transfected into cells using Transfection Reagent. HEK293T cells were transfected with Biobest (BB0002, BioBEST Biotech), and A549 cells were transfected with JetPRIME (PT‐114‐15, Polyplus), following the manufacturers’ protocols.

### Virus Infection

Influenza A virus (IAV) at a multiplicity of infection (MOI) of 3, SeV at 25 hemagglutination (HA) units, or eGFP‐tagged VSV at a MOI of 0.1 were added to fresh serum‐free medium, and the cells were incubated at 37 °C in 5% CO_2_ (v/v) for 1 h. Subsequently, the cells were washed with phosphate‐buffered saline (PBS; 137 mm NaCl (Sinopharm Chemical Reagent Co., Ltd, 10019318), 10 mm Na_2_HPO_4_ (Sinopharm Chemical Reagent Co., Ltd, 10020318), 1.8 mm KH_2_PO_4_ (Sinopharm Chemical Reagent Co., Ltd, 10017618), pH 7.4), 2.7 mm KCl (Sinopharm Chemical Reagent Co., Ltd, 10016318) and cultured in fresh medium supplemented with 2% fetal bovine serum (FBS). Cells were collected and lysed at the indicated time points in Radioimmunoprecipitation assay (RIPA) lysis buffer (P0013C, Beyotime) for Western blot analysis.

### Mouse Husbandry

Three‐week‐old C57BL/6N background mice used for generating lung‐specific *Api5* knockdown and overexpression were purchased from Shanghai SLAC Laboratory. Eight‐week‐old wild‐type and *Api5* ^−/−^ mice were purchased from Cyagen Biosciences Inc. (Suzhou, China). All mice were raised in individually ventilated cages in the animal facilities of Zhejiang University Center for Veterinary Sciences Good Clinical Practice Animal Laboratory (Approval No. SYXK‐2022‐0033). The mice were maintained under controlled conditions (20–26 °C, 40–60% humidity) with a 12/12‐hour light/dark cycle and ad libitum access to food and water throughout the study period.

### Animal Experiments

The animal experiments were conducted in accordance with procedures approved by the Animal Ethical and Welfare Committee for Institutional Animal Care and Use Committee (IACUC) of Zhejiang University (Approval No. ZJU202210029). Three‐week‐old C57BL/6N mice were intranasally infected with 10^11^ TCID_50_ per mouse of AAV6 carrying either: sh*Api5* (*Api5*‐targeting shRNA), shCON (scrambled shRNA control), human Flag‐API5, Flag‐API5^S464A^ recombinant protein, or empty vector. Four weeks post‐inoculation, mice were intranasally challenged with influenza virus (IAV) H1N1 (5×10^5^ PFU (plaque‐forming unit) mL^−1^; 50 µL per mouse) or VSV (10^8.0^ PFU mL^−1^; 60 µL per mouse). IAV‐infected mice (n = 10 mice/group) were monitored daily for survival over 14 days. Four days post‐infection (dpi), IAV‐ or VSV‐infected mice (n = 6 mice/group) were euthanized for lung lesion assessment and viral titer. Simultaneously, lung tissue from IAV‐, VSV‐, and mock‐infected mice (n = 6 mice/group) were fixed in 4% paraformaldehyde, processed into sections with hematoxylin‐eosin (HE) staining by Hawk Bioscience. The images shown are representative of six mice per group. For each IAV‐ or VSV‐infected mouse (n = 6 mice/group), lung tissue per mouse was homogenized. A 20 mg aliquot was lysed in TRIzol for mRNA‐level analysis of *Ifnb1*, *Cxcl‐10*, *Il‐6*, and *Tnfa* at 4 dpi, while a 100 mg aliquot was used for viral titer determination. Additionally, BAL was collected from mouse lungs (n = 6 mice/group) infected with IAV per group to measure IFN‐β, CXCL10, IL‐6, and TNFα proteins at 4 dpi.

In a separate experiment, WT and *Api5* ^−/−^ mice were intranasally infected with IAV (5×10^5^ PFU mL^−1^; 50 µL per mouse). At 4 dpi, IAV‐infected mice lungs (n = 3 mice/group) were collected for gross lesion evaluation, histopathological analysis, and quantification of IFN‐β, CXCL10, IL‐6, and TNFα at both mRNA and protein levels, using the same methods described above.

### Luciferase Reporter Assay

HEK293T cells were seeded onto 24‐well plates and transfected the following day using the Biobest transfection reagent. Co‐transfection was carried out with reporter plasmids (100 ng), pRL‐TK (5 ng), and the specified plasmids (200 ng). At 24 h after transfection, cells were exposed to various stimulators for the indicated time points. Luciferase activity was assessed using the dual luciferase assay kit according to the manufacturer's instructions. Data were shown as means ± SEM (n = 3 biological replicates).

### Viral Titer Detection

Cells were infected with IAV (MOI = 3), SeV (25 HA units), or VSV (MOI = 0.1). At different time points after infection, the culture media were collected and then centrifuged at 10 000 × *g* for 10 min at 4 °C. AAV6‐shCON, AAV6‐sh*Api5*, AAV6‐Flag‐API5, AAV6‐Flag‐API5^S464A^ and AAV6‐empty vector C57BL/6N mice were intranasally infected with IAV virus (5×10^5^ PFU mL^−1^; 50 µL per mouse) for 4 days. The lung samples were homogenized in sterile phosphate‐buffered saline (1:3, w/v), and the mixture was then frozen and thawed three times and finally centrifuged at 10 000 × *g* for 10 min at 4 °C. The supernatants after centrifugation were used for TCID_50_ detection. Briefly, the viral suspension was serially diluted tenfold using DMEM containing 2% FBS and then added to fresh A549 cells. Eight repeats of each diluted sample were tested. At 72 h after infection, the cells infected with IAV were subjected to an IFA (immunofluorescence assay) with anti‐M2 mouse mAbs (1:1000 dilution).^[^ ^78^ ^]^ Virus titers of IAV and VSV were separately determined by observing M2 fluorescence and cytopathic effects (CPE), and calculated the TCID_50_ per 0.1 mL. Data were shown as means ± SEM (n ≥ 3 biological replicates).

### Immunoblot Analysis

The procedure was performed as previously described.^[^ ^17^ ^]^ Briefly, equal amounts of total protein from each sample were separated by SDS‐PAGE and transferred to nitrocellulose membranes (10600001, GE Healthcare Life Sciences).

After blocking with 5% nonfat dry milk containing 0.1% Tween 20 (P1379‐500 mL, Sigma–Aldrich) for 1 h at room temperature, the membranes were incubated with primary antibodies for overnight at 4 °C, followed by incubation with horseradish peroxidase (HRP)‐conjugated anti‐mouse/rabbit IgG and visualization using Superkine Chemiluminescence Substrate (BMU102‐CNAbbkine). β‐Actin or GAPDH expression served as a loading control for normalization. Protein bands were quantified by Image J software, and shown as mean ± SEM (n ≥ 3 independent experiments).

### Native PAGE for IRF3 Dimer

WT and *API5* ^−/‐^ A549 cells were individually infected with IAV (MOI = 3), SeV (25 HA units), or VSV (MOI = 0.1). The cells were harvested at different time points following infection to detect IRF3 dimer. The subsequent experimental procedure was conducted as previously described.^[^ ^79^ ^]^ Briefly, 5×10^6^ cells were lysed in 250 µL RIPA buffer (P0013D, Beyotime) supplemented with phenylmethylsulfonyl fluoride (PMSF; P8340, Solarbio) and stored overnight at −80 °C. Following three freeze‐thaw cycles, the lysates were centrifuged (15 000 × *g*, 10 min, 4 °C) and the supernatant was split into two aliquots. The first aliquot was mixed with native sample buffer (1 m Tris‐HCl pH 6.8, 0.02% bromophenol blue, 50% glycerol) for IRF3 dimer analysis: A native PAGE gel was pre‐run at 70 V for 30 min in 25 mm Tris‐HCl/192 mm glycine (pH 8.4), with 1% deoxycholate (DOC) added exclusively to the cathode chamber. Samples were then loaded and electrophoresed for 3 h at 70 V. Proteins were transferred to a nitrocellulose membrane using transfer buffer (25 mm Tris‐HCl, 192 mm glycine, 20% methanol), followed by standard immunoblotting procedures. The second aliquot was combined with SDS sample buffer for Western blotting with an anti‐IRF3 rabbit polyclonal antibody. GAPDH expression was assessed as a loading control.

### Quantitative Real‐Time Polymerase Chain Reaction (qRT‐PCR) Assay

Cells treated with various stimuli and mice lung samples with virus infection were harvested in TRIzol. Total RNA was extracted and reverse‐transcribed into cDNA using PrimeScript RT reagent Kit (RR047A, Takara). The cDNA was then subjected to qPCR using TB Green Premix Ex Taq (RR420A, Takara). Data shown are the relative abundance of the indicated mRNAs normalized to that of *GAPDH* mRNA. Data were analyzed by the 2^−ΔΔCt^ method and shown as means ± SEM (n ≥ 3 biological replicates). The primers used are listed in Table S2 (Supporting Information).

### ELISA Assay

WT and *API5* ^−/‐^ A549 cells were stimulated with IAV (MOI = 3), SeV (25 HA units), or VSV (MOI = 0.1) for 12 h. WT, *Api5* ^−/−^, AAV6‐shCON, AAV6‐sh*Api5*, AAV6‐Flag‐API5, AAV6‐Flag‐API5^S464A,^ and AAV6‐empty vector C57BL/6 mice were intranasally infected with IAV (5×10^5^ PFU mL^−1^; 50 µL per mouse) for 4 day. The culture media and the bronchoalveolar lavage fluid were collected, and IFN‐β, IL‐6, CXCL10, and TNF‐α protein levels were measured using ELISA kits according to the manufacturer's instructions. Data were shown as means ± SEM (n ≥ 3 biological replicates).

### CRISPR‐Cas9 Knockout

The target sequences of different genes were separately inserted into the guide RNA expression plasmid PX459 (62 988, Addgene; deposited by Feng Zhang). The recombinant constructs were transfected individually into the indicated cells using transfection reagent. At 36 h after transfection, the cells were selected using puromycin (10 µg mL^−1^) for 48 h. Subsequently, monoclonal cells were obtained using the limiting dilution method and identified by immunoblotting assays with indicated antibodies. Additionally, *API*5^−/‐^ A549 cells were verified by DNA sequencing and T7 Endonuclease assay (EN303‐01, Vazyme Biotech Co., Ltd). The primers used for the identification of *API5* ^−/‐^ A549 cells are as follows: *API5*‐F: 5ʹ‐GACTCATCCATCTCC‐

CCCAACTC‐3ʹ and *API5*‐R: 5ʹ‐GTGAGCCACAACCCATTTTTAACAG‐3ʹ. The oligonucleotides used to construct the knockout cell lines and all cell lines employed are listed in Table S3 (Supporting Information).

### Lentivirus Mediated Gene Transfer

*API5* knockdown cell lines (THP‐1, WT, and *p62* ^−/‐^ A549 cells) were generated by lentiviral‐mediated RNA interference. Briefly, the *API*5 gene target sequence 5ʹ‐GGCCGACCTAGAACAGACCTT‐3ʹ and scramble control sequence 5ʹ‐CGGATCGCTACAAATAAG‐3ʹ were inserted into the lentiviral vector pGreenPuro shRNA (SI505A‐1, Biovector Science Lab, Inc.), respectively. WT and *API5* ^−/−^ A549 cell lines with stable expressing GFP‐LC3 were generated with lentiviral vector pCDH‐CMV‐MCS‐EF1 (CD5210B‐1, System Biosciences) followed by the insertion of GFP‐LC3 fragment. In brief, the GFP‐LC3 fragment was subcloned into the lentiviral vector pCDH‐CMV‐MCS‐EF1. The resultant recombinant lentiviral plasmids, along with psPAX2 (12 260, Addgene) and pMD2.G (12 259, Addgene) packaging plasmids, were separately co‐transfected into HEK293KT cells using Biobest transfection reagent for 48 h. After centrifugation at 10 000 × *g* for 10 min at 4 °C, the supernatants were added to specified cells (THP‐1, WT, and *p62* ^−/−^ A549 cells). At 24 h after infection, the resultant cells were selected with puromycin (5 µg mL^−1^; 58‐58‐2, Invivogen,) for 1 week to obtain *API*5‐silencing THP‐1, WT, and *p62* ^−/‐^ A549 cells, and GFP‐LC3 overexpressing WT and *API5* ^−/−^ A549 cells. Finally, these cell lines were confirmed by immunoblotting with indicated antibodies or GFP protein fluorescence observation. All generated knockdown and stable expression cell lines are listed in the Table S3 (Supporting Information).

### Mass Spectrometry (MS)

To identify phosphorylation sites on API5 and the host protein kinases interacting with API5, HEK293T cells were transfected with Flag‐API5 for 24 h, following infection with IAV (MOI = 5) for 12 h. The cells were lysed in NP‐40 buffer (P0013F, Beyotime) supplemented with PMSF (P8340, Solarbio). The supernatant was subjected to immunoprecipitation assays with ANTI‐FLAG M2 Agarose Affinity Gel (A2220, Sigma–Aldrich). The immunoprecipitation complexes were separated by SDS‐PAGE and stained with Coomassie Brilliant Blue R250. Subsequently, the staining gels were manually excised and subjected to Liquid Chromatography Mass Spectrometry (LC‐MS) analysis in APTBio (Shanghai, China).

### Protein Purification and GST Affinity‐Isolation

*Escherichia coli* BL21 (pLysS) (B528415‐0010, Sangon Biotechnology) harboring pGEX‐4T‐1‐API5 or pET‐28a‐SRPK1 was cultured in 200 mL LB medium (l0 g L^−1^ Tryptone [A505250‐0500, Sangon Biotechnology], 5 g L^−1^ Yeast extract [A515245‐0500], and 10 g L^−1^ NaCl [10 019 318, Sinopharm Chemical Reagent Co., Ltd]) and induced with 1 mm isopropyl β‐d‐thiogalactopyranoside (IPTG; A600168‐0025, Sangon Biotechnology) at 16 °C with shaking at 90 rpm overnight. Cell pellets were lysed by sonication in the binding buffer. For His‐SRPK1 purification, the lysate was incubated with Ni‐NTA agarose (30 210, QIAGEN) in binding buffer containing 50 mm Tris‐HCl (pH 8.0) and 10 mm imidazole. After extensive washing, the target protein was eluted with the same buffer containing 80 mm imidazole. For GST and GST‐API5 purification, the supernatant was mixed with Glutathione (GST) Resin (L00206, GenScript) in binding buffer (50 mm Tris‐HCl, pH 8.0, 150 mm NaCl), and the fusion proteins were eluted with 10 mg/5 mL reduced glutathione (A100399‐0005, Sangon Biotechnology) in the same buffer. Purified GST (20 ng) and GST‐API5 (20 ng) proteins were individually incubated with His‐SRPK1 (50 ng) at 4 °C for 4 h. Subsequently, GST resin (60 µL) was added to capture the protein complexes. After incubation, the resin was pelleted by centrifugation and washed five times with NP‐40 lysis buffer (P0013F, Beyotime) at 4 °C to remove non‐specific interactions, and then lysed for immunoblotting analysis.

### In Vitro Kinase Assay

His‐SRPK1, kinase‐dead mutant His‐SRPK1^K109A^, and GST‐API5 proteins were purified from *Escherichia coli* BL21 (pLysS) cells harboring pET‐28a‐SRPK1, pET‐28a‐SRPK1^K109A^, or pGEX‐4T‐1‐API5 plasmids. Subsequently, an in vitro kinase assay was conducted as previously described.^[^ ^80^ ^]^ Briefly, 500 µL of kinase buffer (50 mm Tris‐HCl, pH 7.5, 10 mm MgCl_2_, 50 mm NaCl, 10 mm β‐glycerophosphate disodium salt hydrate [G9422‐10G, Sigma‐Aldrich], 2 mm 1,4‐dithio‐DL‐threitol [DTT; 3483‐12‐3, Solarbio], supplemented with 5 mm ATP [D7378‐1 mL, Beyotime]), was mixed with GST‐API5 (20 µg) and 40 µg of His‐SRPK1 or mutant His‐SRPK1^K109A^. The mixture was incubated at 30 °C for 40 min. After incubation, 60 µL reaction production was stopped by adding 4× SDS sample loading buffer (P1016, Solarbio) and subjected to SDS‐PAGE followed by specified immunoblotting. The remaining reaction mixture was inactivated at 95 °C for 4 min and then subjected to GST affinity‐isolation assay. The pellets were washed using NP‐40 lysis buffer (P0013F, Beyotime), and the samples were then placed in 4× SDS sample loading buffer and boiled for 10 min. Finally, the samples were fractionated by 10% SDS‐PAGE followed by western blotting with the indicated antibodies.

### In Vitro Deubiquitylation Assay

His‐tagged E1(UBA1), UBE2D2, UBE2D3, ubiquitin, Flag‐p62 proteins were purified from *Escherichia coli* BL21 (pLysS) cells. Subsequently, in vitro ubiquitylation assay was conducted as previously described.^[^ ^17^ ^]^ Briefly, 500 µL of ubiquitination buffer (20 mm Tris‐HCl, pH 7.5, 5 mm MgCl2, 2 mm DTT, supplemented with 2 mm ATP) was mixed with ubiquitin (50 µg), UBA1 (2 µg), UBE2D2/ UBE2D2 (3 µg), TRIM21 (20 µg), and His‐Flag‐p62 (20 µg). The mixture was incubated to synthesize ubiquitinated p62 at 37 °C for 2 h. The ubiquitinated p62 was enriched by anti‐FLAG M2 Agarose Affinity Gel (A2220, Sigma‐Aldrich). After extensive washing with RIPA buffer (P0013C, Beyotime), the proteins were eluted with 3× Flag Peptide (P9801, Beyotime). The recombinant Flag‐tagged API5^WT^ and mutant API5^S464A^ were purified from API5^WT^ or API5^S464A^ overexpressing IAV (MOI = 5) infected‐HEK293T cells by anti‐FLAG M2 Agarose Affinity in NP‐40 lysis buffer (P0013F, Beyotime). Ubiquitinated p62 generated in vitro was incubated with Flag‐API5^WT^, Flag‐API5^S464A^ in the deubiquitination buffer (50 mm Tris‐HCl [pH 8.0], 50 mm NaCl, 1 mm EDTA, 10 mm DTT, 5% glycerol) for 1 h at 37 °C, and analyzed by Western blotting.

### Subcellular Fractionation

The procedure was conducted as previously described.^[^ ^79^ ^]^ WT and *API5* ^−/‐^ A549 cells were infected with IAV (MOI = 3), SeV (25 HA units), or VSV (MOI = 0.1). Cells were harvested at the indicated time points for nuclear and cytoplasmic fractionation. Briefly, 5×10^6^ cells were lysed in 250 µL 0.1% NP‐40 lysis buffer (P0013F, Beyotime) diluted in phosphate‐buffered saline (PBS; 137 mm NaCl [Sinopharm Chemical Reagent Co., Ltd, 10019318], 10 mm Na_2_HPO_4_ [Sinopharm Chemical Reagent Co., Ltd, 10020318], 1.8 mm KH_2_PO_4_ [Sinopharm Chemical Reagent Co., Ltd, 10017618], pH 7.4), 2.7 mm KCl [Sinopharm Chemical Reagent Co., Ltd, 10016318). After centrifugation (1000 × *g*, 5 min), the supernatant was transferred and centrifuged again (15 000 × *g*, 10 min, 4 °C) to obtain the cytoplasmic fraction. The pellet was washed twice with ice‐cold 0.1% NP‐40 lysis buffer, and the remaining pellet was lysed in 120 µL RIPA buffer (P0013C, Beyotime) to extract the nuclear fraction.

### Immunofluorescent Assay (IFA) and Confocal Microscopy

For the IFA, A549 cells grown on a 96‐well plate were infected with IAV for 72 h. The cells were fixed with a methanol‐acetone mixture (1:1 [vol/vol]) at −20 °C for 20 min. The resultant cells were blocked with 5% skim milk diluted in phosphate‐buffered saline (PBS; 137 mm NaCl [Sinopharm Chemical Reagent Co., Ltd, 10019318], 10 mm Na_2_HPO_4_ [Sinopharm Chemical Reagent Co., Ltd, 10020318], 1.8 mm KH_2_PO_4_ [Sinopharm Chemical Reagent Co., Ltd, 10017618], pH 7.4), 2.7 mm KCl [Sinopharm Chemical Reagent Co., Ltd, 10 016 318) containing 0.1% Tween 20 (Amresco, 0777‐1 L) for 30 min at 37 °C and incubated with mouse anti‐M2 mAb (1:1000 dilution) for 2 h at 37 °C, followed by incubation with FITC‐conjugated goat anti‐mouse IgG (1:400 dilution) for 1.0 h at 37 °C. Viral titers were determined by observing infected cells under a fluorescence microscope (OLYMPUS, U‐RFL‐T) and calculating the TCID_50_ per 0.1 mL. Confocal microscopy was used to analyze GFP‐LC3 puncta formation, p62 aggregation, colocalization of p62 with GFP‐LC3, as well as API5 colocalization with both RIG‐I and MDA5. WT and *API5* ^−/−^ A549 cells with stable expressing GFP‐LC3, WT and *API5* ^−/−^ A549 cells transfected with mCherry‐API5^WT^, mCherry‐API5^S464A^, were infected with IAV (MOI = 3) for 12 h, the cells were fixed with 4% paraformaldehyde for 10 min at room temperature and then incubated overnight at 4 °C with the following primary antibodies: rabbit anti‐p62 (ab109012, Abcam; 1:200 dilution), rabbit anti‐API5 (ab65836, Abcam; 1:200 dilution) and mouse anti‐RIG‐I (sc‐376845, Santa Cruz; 1:100 dilution), and mouse anti‐API5 (sc‐101203, Santa Cruz; 1:100 dilution) and rabbit anti‐MDA5 (A13645, ABclonal; 1:200 dilution). After being washed three times with phosphate‐buffered saline (PBS; 137 mm NaCl [Sinopharm Chemical Reagent Co., Ltd, 10 019 318], 10 mm Na_2_HPO_4_ [Sinopharm Chemical Reagent Co., Ltd, 10 020 318], 1.8 mm KH_2_PO_4_ [Sinopharm Chemical Reagent Co., Ltd, 10 017 618], pH 7.4), 2.7 mm KCl [Sinopharm Chemical Reagent Co., Ltd, 10 016 318) containing 0.1% Tween 20 (Amresco, 0777‐1 L), cells were further incubated for 1 h at room temperature with the following secondary antibodies: Alexa Fluor 546‐conjugated donkey anti‐rabbit (A10040, Invitrogen; 1:500 dilution), FITC‐labeled goat anti‐rabbit IgG (172‐1506, Kirkegaard & Perry Laboratories; 1:400 dilution), Alexa Fluor 546‐conjugated donkey anti‐rabbit (A10040, Invitrogen; 1:500 dilution) and FITC‐labeled goat anti‐mouse IgG (172‐1806, Kirkegaard & Perry Laboratories;1:400 dilution), and Alexa Fluor 546‐conjugated donkey anti‐mouse (A10036, Invitrogen; 1:500 dilution) and FITC‐labeled goat anti‐rabbit IgG (172‐1506, Kirkegaard & Perry Laboratories; 1:400 dilution). Cellular nuclei were stained with 4ʹ,6ʹ‐diamidino‐2‐phenylindole (DAPI; 28718‐90‐3, BioFroxx; 1 mg mL^−1^, 1:1000 dilution) for 5 min. Finally, the GFP‐LC3 puncta (a key autophagy marker),^[^ ^60^ ^]^ p62 aggregates, their colocalization, and API5 colocalization with RIG‐I and MDA5 were imaged using an LSM780 laser scanning confocal microscope (Zeiss, Oberkochen, Germany) under a ×60 oil objective.

### Statistical Analysis

In this study, statistical analysis and presentation graphics were carried out by the GraphPad Prism 9.0 software. The Kaplan‐Meier method was employed for animal survival analysis, and the survival curves were analyzed using log‐rank (Mantel‐Cox) analysis. For Western blot analysis, protein bands were quantified by measuring the mean gray values within the linear range, with background subtraction, using Image J software. The results were presented as the means ± SEM from at least three biological replicates. Statistical significance was analyzed by unpaired two‐tailed Student's t test or two‐way ANOVA. The *p* values< 0.05 are deemed statistically significant difference (*p < 0.05; **p < 0.01; ***p < 0.001; ns, no significant).

## Conflict of Interest

The authors declare no conflict of interest.

## Author Contributions

Tingjuan Deng performed most experiments, and Jianan Xu., Linglong Qin., Xingbo Wang., Chenhe Lu., Yanming Huang., and Da Liu. contributed to several experiments. Tingjuan Deng. and Jianan Xu. quantified the protein bands in all Western blot experiments, performed the statistical analysis, and data presentation. Yan Yan. and Weiren Dong. participated in the analysis of several experimental data. Pinglong Xu. offered critical reviews and revision of the manuscript. Jiyong Zhou. and Tingjuan Deng. conceived the study and experimental design, and Jiyong Zhou. and Tingjuan Deng. wrote the manuscript.

## Supporting information



Supporting Information

## Data Availability

The data that support the findings of this study are available from the corresponding author upon reasonable request.
