# Supplementary material for: API5 Phosphorylation Promotes Antiviral Immunity by Inhibiting Degradation of Cytosolic RNA Sensor RLRs
Source: Adv Sci (Weinh). 2025 Jul 11;12(37):e05479. doi: 10.1002/advs.202505479 (PMC12499447; doi:10.1002/advs.202505479)
Supplement: Supplementary file 1 — Supporting Information [file ADVS-12-e05479-s001.docx]

**Supporting Information**

**API5 Phosphorylation Promotes Antiviral Immunity by Inhibiting Degradation**

**of Cytosolic RNA Sensor RLRs**

Tingjuan Deng^1,2^, Jianan Xu^1^, Linglong Qin^1^, Xingbo Wang^1^, Chenhe Lu^1^, Yanming Huang^1^, Da Liu^1^, Yan Yan^1^, Weiren Dong^1^, Pinglong Xu^2,3^, Jiyong Zhou^1,2^*

^1^MOA Key Laboratory of Animal Virology, Zhejiang University Center for Veterinary Sciences, Hangzhou, 310058, China

^2^State Key Laboratory for Diagnosis and Treatment of Severe Infectious Diseases, First Affiliated Hospital, Zhejiang University, Hangzhou, 310058, China

^3^Life Sciences Institute, Zhejiang University, Hangzhou, 310058, China

*Correspondence: [jyzhou@zju.edu.cn](mailto:jyzhou@zju.edu.cn)


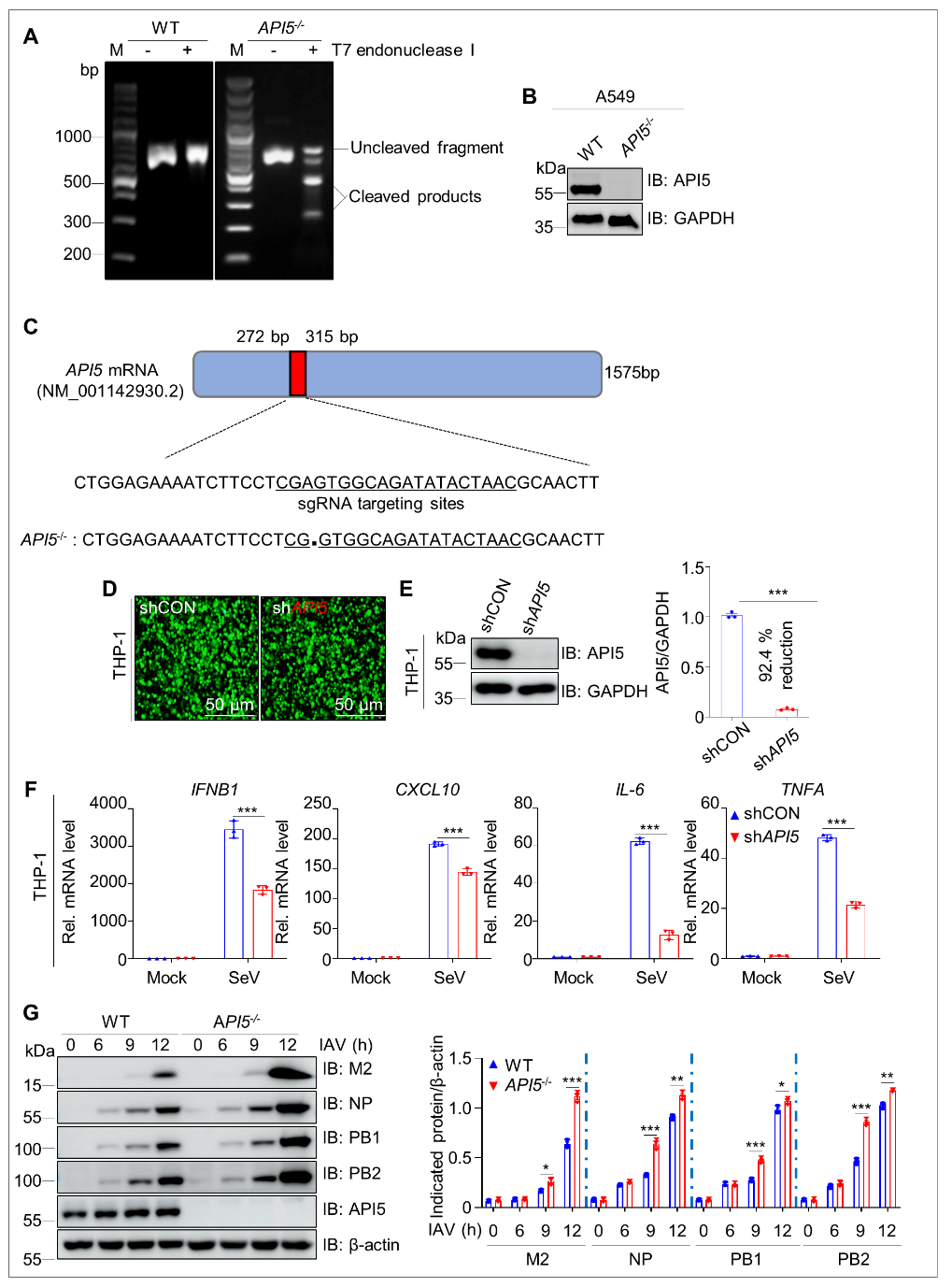


**Figure S1.** API5 promotes innate antiviral signaling. (A-C) Identification of *API5*^-/-^ A549 cells. The genomic DNA and cellular lysates from WT and *API5*^-/-^ A549 cells were separately subjected to T7 endonuclease I (A), Western blotting (B), and sequencing analysis (C). (D and E) The generation of *API5* knockdown THP-1 cells. shCON and sh*API5* THP-1 cells were observed by fluorescence microscopy (D) and were immunoblotted using anti-API5 rabbit pAb (E). Right: the immunoblot protein bands (left) were quantified as the API5/GAPDH ratio. (F) qPCR analysis of mRNA levels of *IFNB1*, *CXCL10*, *IL-6* and *TNF*A in shCON and sh*API5* THP-1 cells induced by SeV infection for 6 h. (G) WT and *API5*^-/-^ A549 cells were infected with IAV (MOI = 3). The cells were lysed in RIPA buffer (P0013C, Beyotime) for immunoblotting at different time points (Left). Right: data were quantified and shown as the ratio of M2 to β-actin, NP to β-actin, PB1 to β-actin and PB2 to β-actin. For B, E, and G, data are one representative of three biological replicates. Data represent the mean ± SEM (n = 3 biological replicates). Statistical significance was analyzed by unpaired two-tailed Student’s t test. (**p* < 0.05; ***p* < 0.01; ****p* < 0.001).


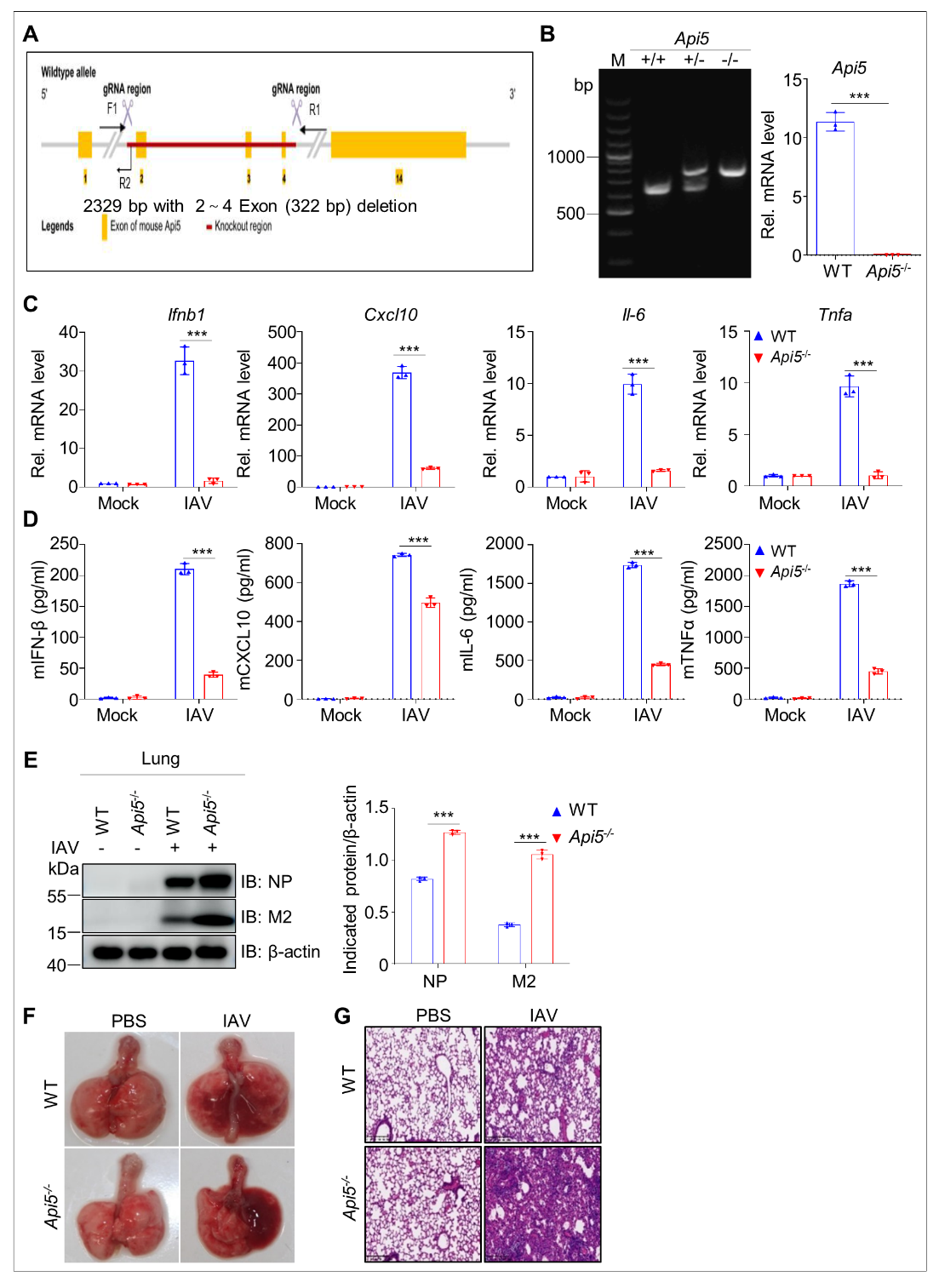

**Figure S2.** *Api5* knockout reduces antiviral defense in mice. (A) Schematic representation of *Api5* knockout strategy. Exons 2 to 4 of the *Api5* gene were deleted, resulting in a frameshift mutation and loss of API5 expression. (B) Genotype identification of the *Api5* wild-type (WT), heterogeneous, and knockout mice, and qPCR analysis of *Api5*^-/-^ mice (Right). (C) WT and *Api5*^-/-^ mouse lung tissues (n = 3 mice/group) infected with IAV were subjected to qPCR analysis for mRNAs of *Ifnb*1, *Cxcl*10, *Il*-6, and *Tnfa*. (D) BAL from IAV- or mock-infected shCON and sh*Api5* mice (n = 3 mice/group) were used for ELISA analysis of IFN-β, CXCL10, IL-6, and TNFa. (E) WT and *Api5*^-/-^ mice were intra-nasally infected with IAV (5×10^5^ PFU/mL; 50 μL per mouse) for 4 days. Viral proteins in the lung of IAV-infected WT and *API5*^-/-^ mice were immunoblotted. Right: data were quantified and shown as the ratio of M2 to β-actin and NP to β-actin. (F) Gross images of the lungs of IAV-infected WT and *Api5*^-/-^ mice at 4 days. The images are from representative one of three mice per group. (G) HE staining of lung tissues from IAV-infected WT and *Api5*^-/-^ mice at 4 days. Scale bar, 200 μm. The images are from representative one of three mice per group. Data were shown as mean ± SEM (n = 3 independent samples). Statistical significance was analyzed by unpaired two-tailed Student’s t test. (****p* < 0.001).


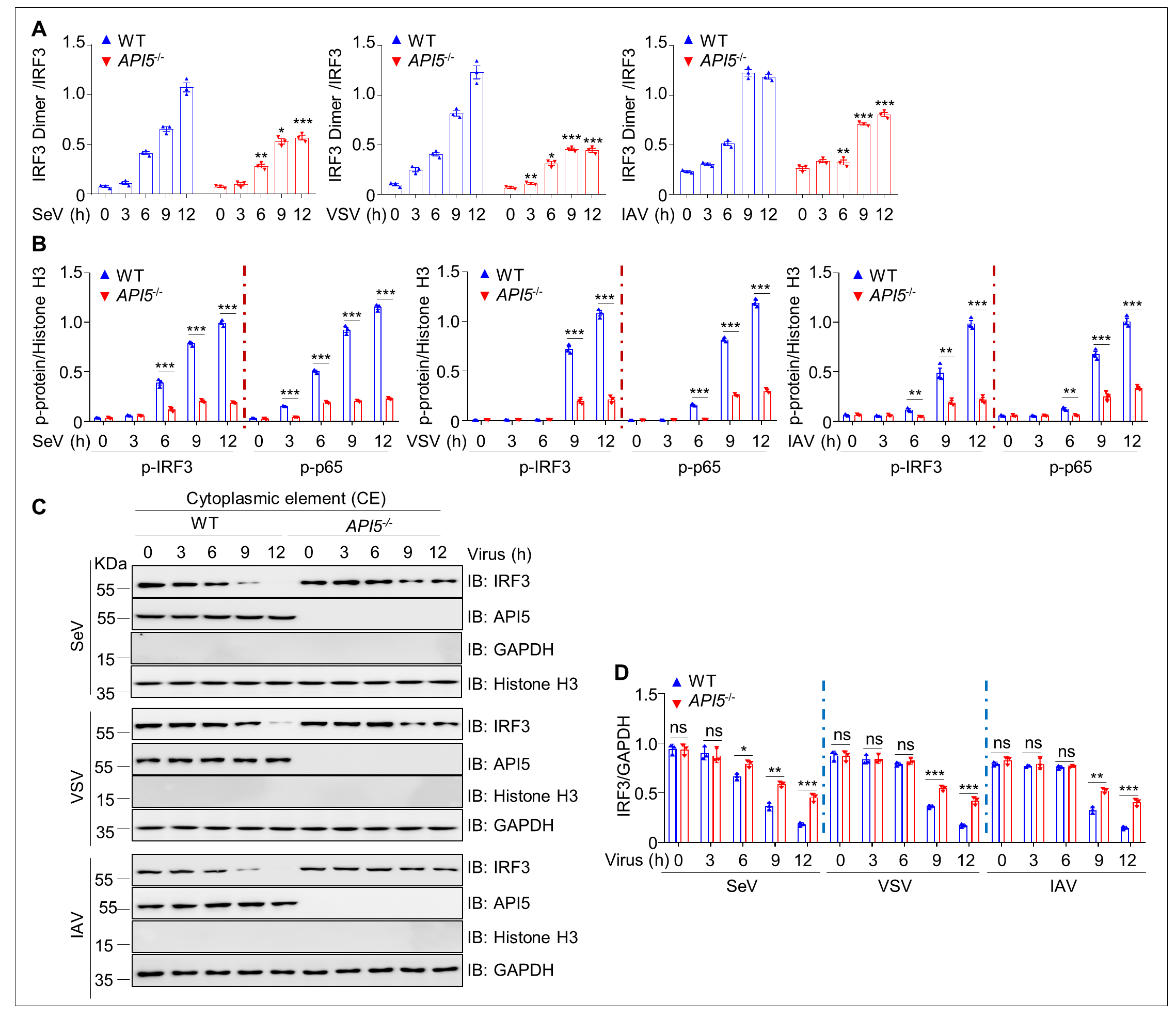


**Figure S3.** *API5* deficiency impaired IRF3 nuclear translocation. (A and B) Data from Figure 3A, B were quantified and shown as the ratio of IRF3 dimer to IRF3 (A), p-IRF3 to Histone H3 and p-p65 to Histone H3 (B). (C) WT and *API5*^-/-^ A549 cells were infected with SeV, VSV, or IAV as described in the methods. The cells were then subjected to subcellular fractionation and immunoblotting using an anti-IRF3 rabbit polyclonal antibody. GAPDH and Histone H3 were used as loading controls for the cytoplasmic and nuclear fractions, respectively. (D) The data from (C) were quantified and shown as the ratio of IRF3 to GAPDH. For C, data is one representative of three biological replicates. Data represent mean ± SEM (n = 3 biological replicates). Statistical significance was determined by unpaired two-tailed Student’s t-test. (**p* < 0.05; ***p* < 0.01; ****p* < 0.001; ns, no significant).

**
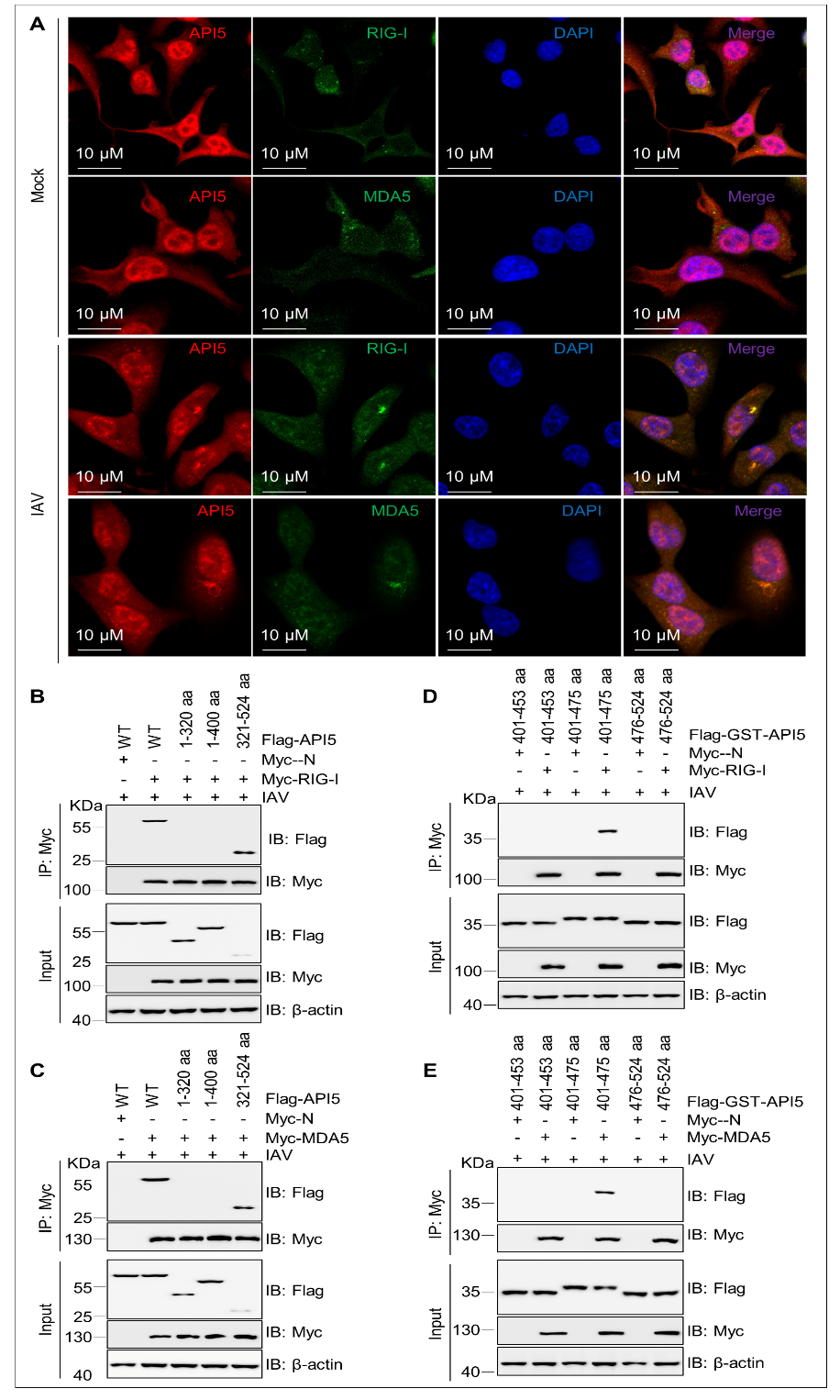
**

**Figure S4.** The residues 454-475 aa of API5 are critical for targeting RIG-I and MDA5. (A) API5 co-localized with RIG-I and MDA5 during IAV infection. A549 cells were infected with IAV (MOI = 3) for 12 h and were fixed for confocal microscopy. (B and C) The residues 321-524 aa of API5 is required for interaction with RIG-I and MDA5. HEK293T cells were co-transfected with Flag-API5 truncation constructs and Myc-tagged RIG-I (B) or MDA5 (C) for 24 h, followed by IAV infection for 12 h. Cell lysates were analyzed by co-IP and immunoblotting using the indicated antibodies. (D and E) The 454-475 aa region of API5 is essential for binding both RIG-I and MDA5. HEK293T cells were co-transfected with Myc-tagged RIG-I (D) or MDA5 (E) together with various Flag-GST-API5 truncation mutants. After 24 h transfection, cells were infected with IAV for 12 h. The lysates were analyzed by co-IP using anti-Flag beads, followed by immunoblotting with the indicated antibodies. Data are one representative of three biological replicates.

**
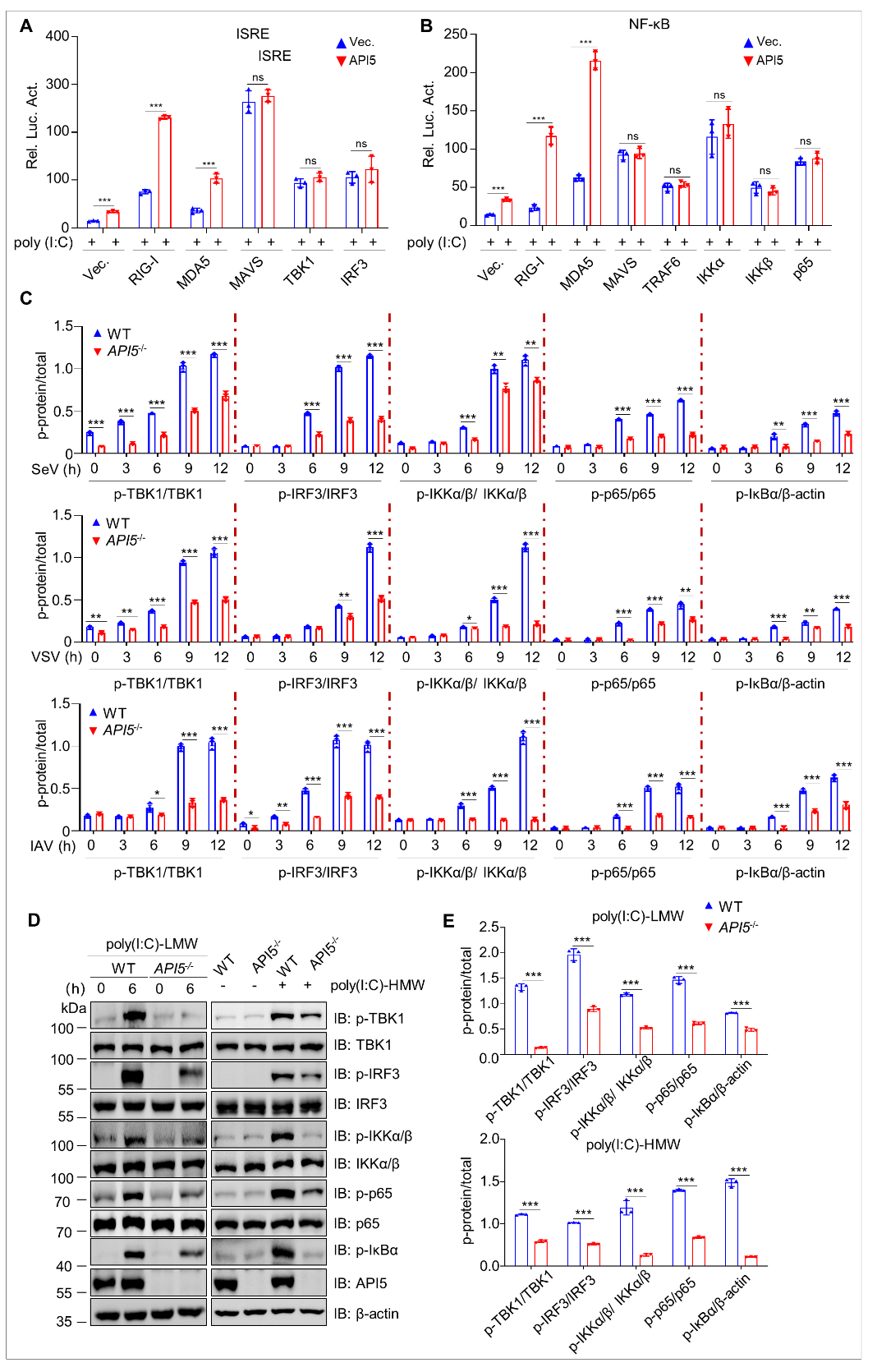
**

**Figure S5.** API5 promotes RLRs-mediated antiviral innate immunity. (A and B) Effects of API5 overexpression on poly(I:C)-triggered activation of ISRE and NF-κB promoters mediated by different signaling molecule. HEK293T cells were co-transfected with ISRE or NF-κB reporter plasmids, pRL-TK, indicated plasmid encoding components of IRF3 (A) or NF-κB (B) pathway and API5 plasmids for 24 h, followed by stimulation with poly(I:C) for 12 h. The cellular lysates were used for luciferase activity analysis. (C) Data from Figure 3F were quantified and shown as the ratio of p-TBK1 toTBK1, p-IRF3 to IRF3, p-IKKα/β to IKKα/β, p-p65 to p65, and p-IκBα to β-actin. (D) WT and *API5*^-/-^ A549 cells were transfected with poly(I:C)-LWM or poly(I:C)-HWM (1ug) for 6 h. The lysates were analyzed by immunoblotting with indicated antibodies. The data are one representative of three biological replicates. (E) The data from (D) were quantified and presented as the ratio of p-TBK1 toTBK1, p-IRF3 to IRF3, p-IKKα/β to IKKα/β, p-p65 to p65, and p-IκBα to β-actin. The data represent the mean ± SEM (n = 3 biological replicates). Statistical significance was analyzed by unpaired two-tailed Student’s t test. (****p* < 0.001; ns, no significant).


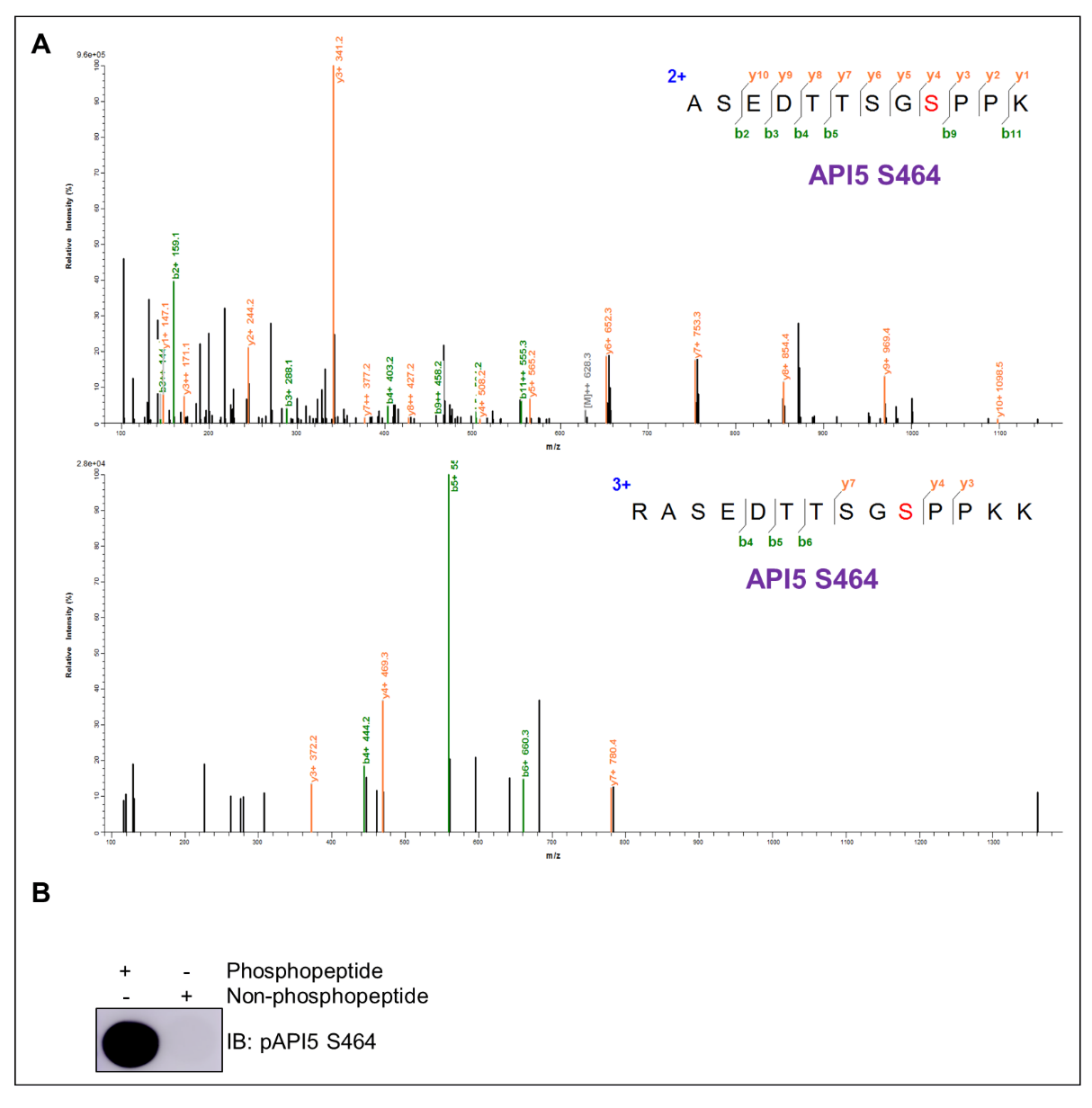


**Figure S6.** Identification of phosphorylation sites of API5. (A) Mass spectrometry identification of phosphorylation site at S464 in API5. The purified Flag-API5 obtained from transfected HEK293T cells with IAV infection by the IP assay was subjected to liquid chromatography-tandem mass spectrometry (LC-MS). The MS spectra of the selected phosphorylated API5 peptides, ASEDTTSGS^464^PPK and RASEDTTSGS^464^PPKK, are shown. (B) The reaction specificity of rabbit anti-pAPI5 (S464) pAb. 5 μg TTSG (S-p) PPKK and TTSGSPPKK peptides were used to Dot blot assays with anti-pAPI5 rabbit pAb. The data are one representative of three biological replicates.


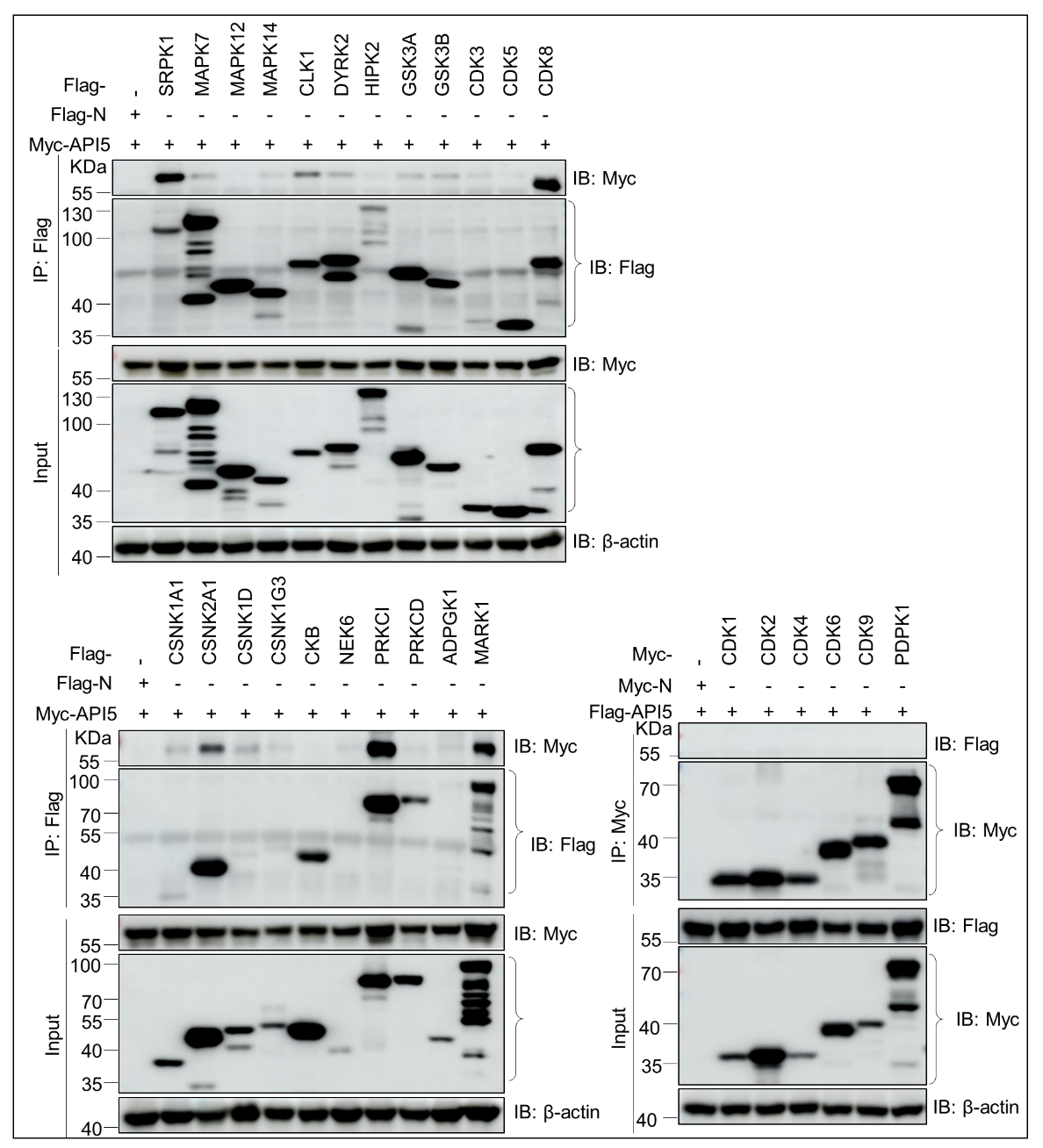


**Figure S7.** Identification of kinase interacting with API5. Different Flag-tagged or Myc-tagged protein kinases and Myc-API5 or Flag-API5 were co-transfected into HEK293T cells for 36 h. The lysates were subjected to co-IP and immunoblotting with indicated antibodies. The data are one representative of three biological replicates.


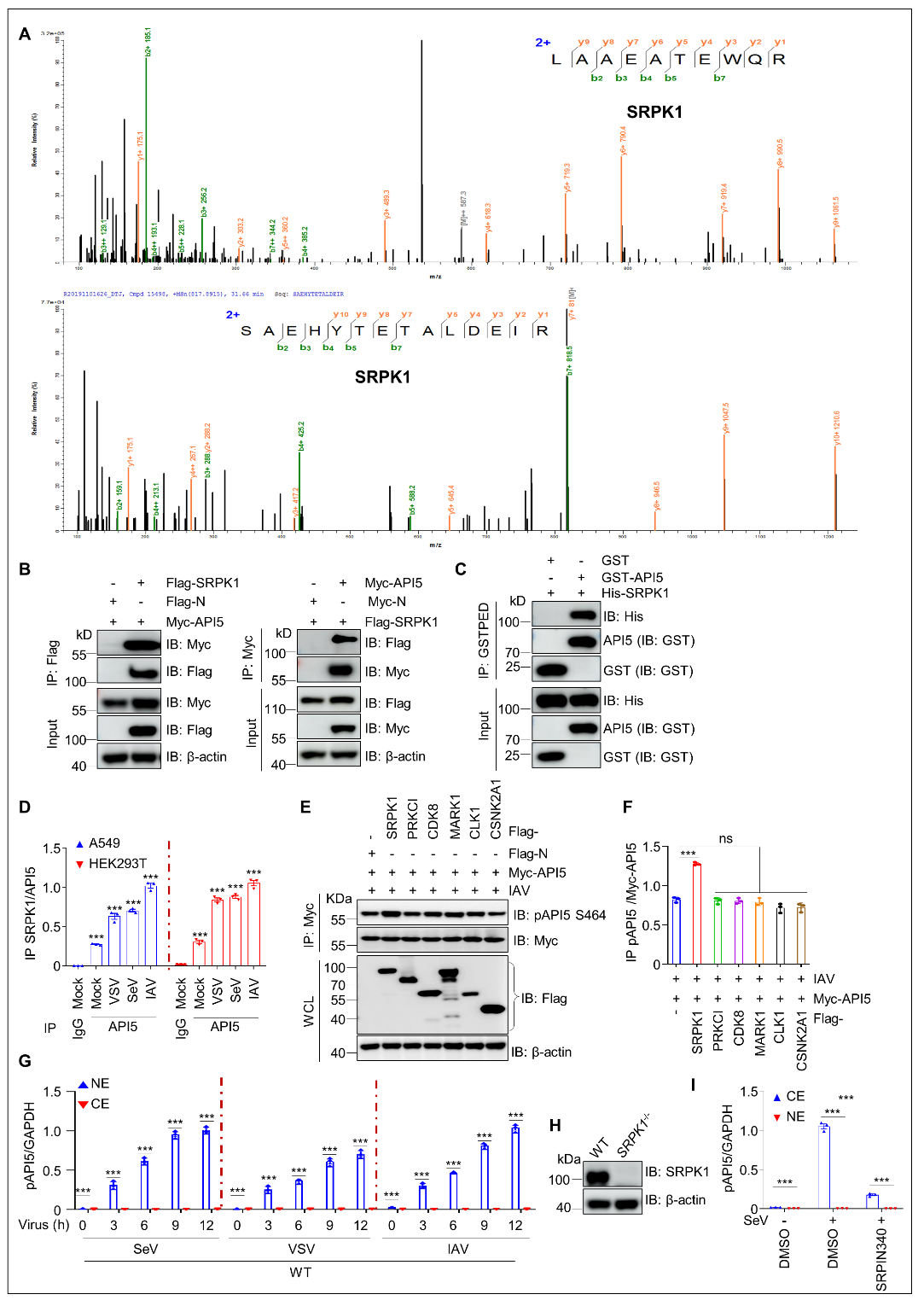


**Figure** **S8.** SRPK1 mediates phosphorylation of API5. (A) Mass spectrometry identification of SRPK1 binding to API5. Mass spectrometry analysis of purified Flag-API5-immunoprecipitated complex from cellular lysates transfected HEK293T cells with IAV infection. The MS spectra of the selected SRPK1 peptides, LAAEATEWQR and SAEHYTETALDEIR, are shown. (B) Co-IP analysis of SRPK1 and API5 in HKE293T cells transfected with Flag-SRPK1 and Myc-API5 for 36 h. (C) API5 directly interacts with SRPK1. GST or GST-API5 and His-SRPK1 purified from *E. coli* BL21 were subjected to GST-affinity-solation assays. (D) The band intensities of SRPK1 in immuno-precipitates (IP) from Figure 4A were quantified on immunoblots and shown as the ratio of SRPK1 to API5. (E) Flag-tagged SRPK1, PRKCI, CDK8, MARK1, CLK1, CSNK2A1 and Myc-API5 were co-transfected into HEK293T cells for 24 h, followed by infection with IAV for 12 h. The cellular lysates were immunoprecipitated with anti-MYC magnetic beads, and then the immunoprecipitation complex was subjected to immunoblotting assays by using anti-Myc and anti-pAPI5 S464 antibodies. (F) Immunoblot band intensities of pAPI5 S464 (D) were quantified and presented as the ratio of pAPI5 to Myc-API5 in IP. (G) Data from Figure 4C were quantified and shown as the ratio of pAPI5 to GAPDH. (H) Immunoblotting analysis of *SRPK1*^-/-^ cells. Lysates from WT and *SRPK1*^-/-^ A549 cells were analyzed with anti-SRPK1 rabbit pAb. (I) Data from Figure 4E were quantified and shown as the ratio of pAPI5 to GAPDH. For A-C, E, H, data are one representative of three biological replicates. Data were shown as mean ± SEM (n = 3 biological replicates). Statistical significance was analyzed by unpaired two-tailed Student’s t test. (****p* < 0.001; ns, no significant).


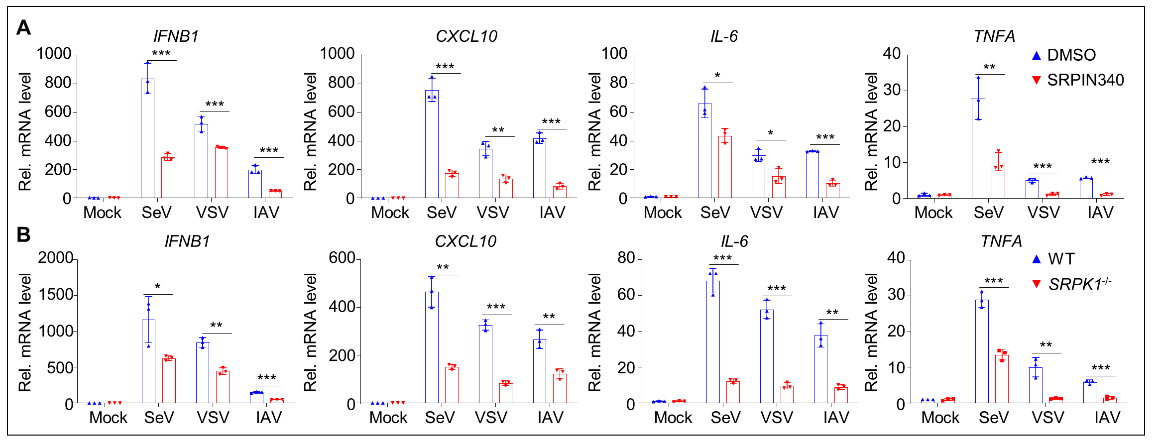


**Figure S9.** SRPK1 mediated-phosphorylation of API5 promotes antiviral signaling. (A) qPCR analysis of *IFNB1*, *CXCL10*, *IL-6* and *TNFA* mRNA in A549 cells treated with DMSO and SRPK1 inhibitor SRPIN340 (10 μM) for 12 h followed by SeV, VSV, or IAV infection for 9 h. (B) qPCR analysis of *IFNB1*, *CXCL10*, *IL-6* and *TNFA* mRNA in WT and *SRPK1*^-/-^ A549 cells with SeV, VSV, or IAV infection for 9 h. Data were shown as mean ± SEM (n = 3 biological replicates). Statistical significance was analyzed by unpaired two-tailed Student’s t test. (**p* < 0.05; ***p* < 0.01; ****p* < 0.001).

**
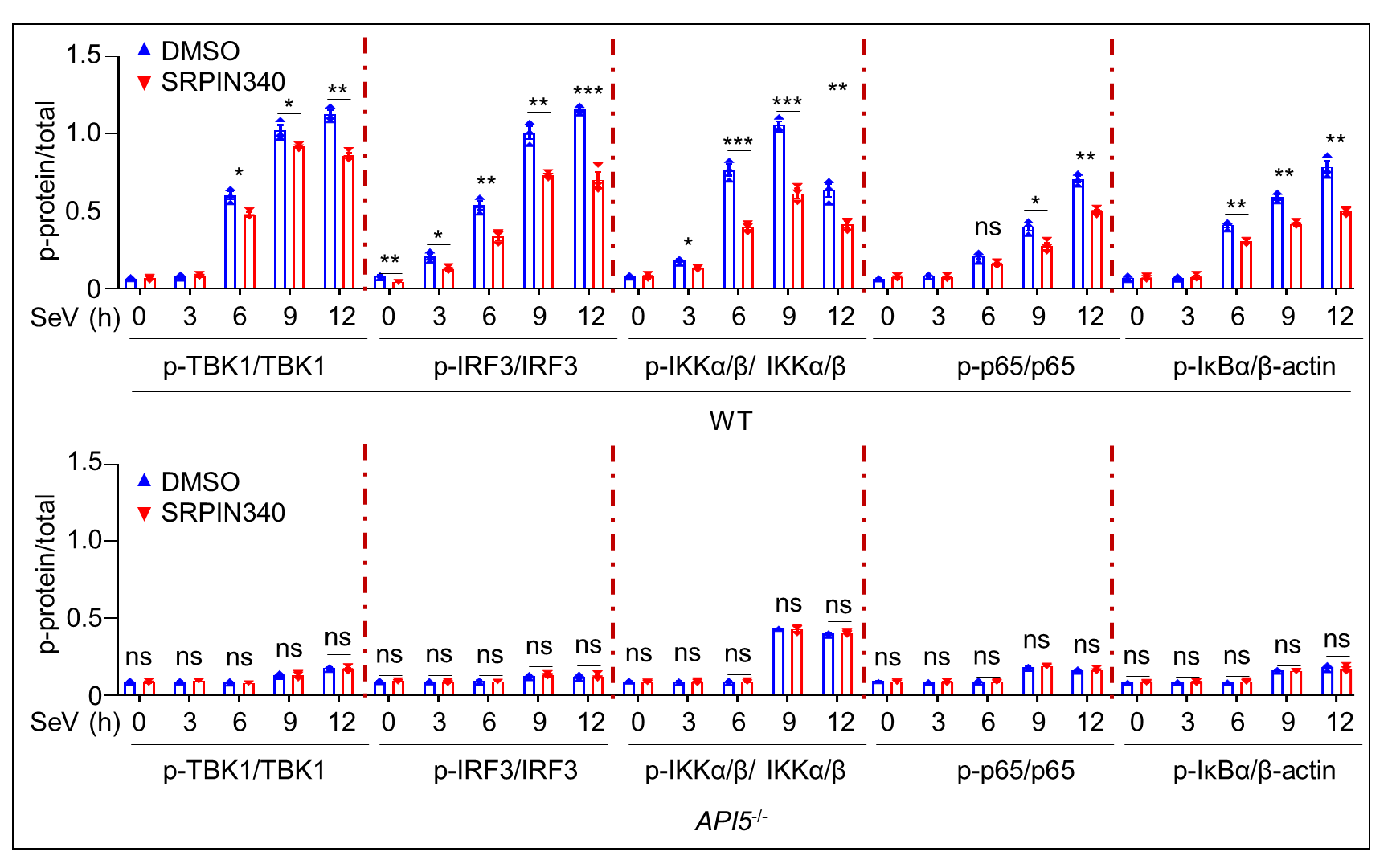
Figure S10.** Densitometric analysis of immunoblots shown in Figure 4F. Data were quantified and presented as the ratio of p-TBK1 toTBK1, p-IRF3 to IRF3, p-IKKα/β to IKKα/β, p-p65 to p65, and p-IκBα to β-actin. The data represent the mean ± SEM (n = 3 biological replicates). Statistical significance was determined by unpaired two-tailed Student’s t-test. (**p* < 0.05; ***p* < 0.01; ****p* < 0.001; ns, no significant).

**
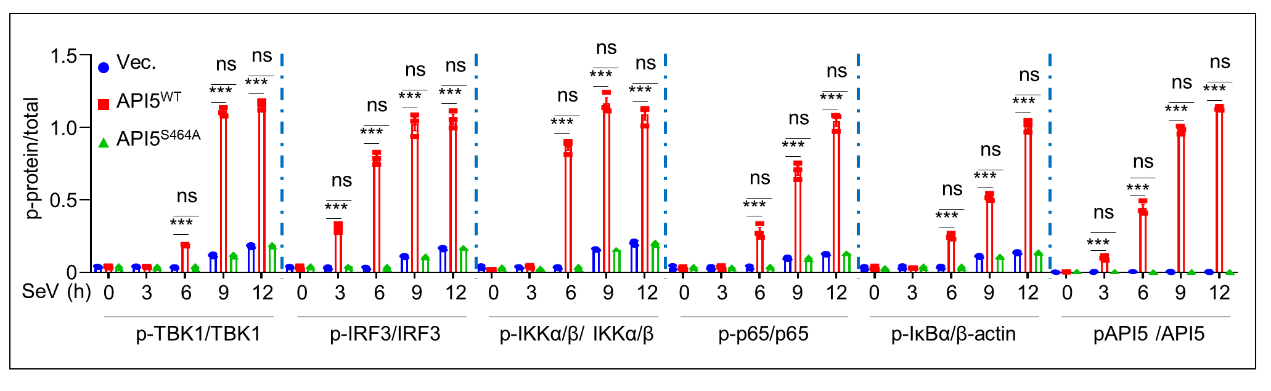
**

**Figure S11.** Densitometric analysis of immunoblots presented in Figure 5E. Data were quantified and presented as the ratio of p-TBK1 toTBK1, p-IRF3 to IRF3, p-IKKα/β to IKKα/β, p-p65 to p65, p-IκBα to β-actin and pAPI5 to API5. Data represent the mean ± SEM (n = 3 biological replicates). Statistical significance was determined by unpaired two-tailed Student’s t-test. (****p* < 0.001; ns, no significant).

**
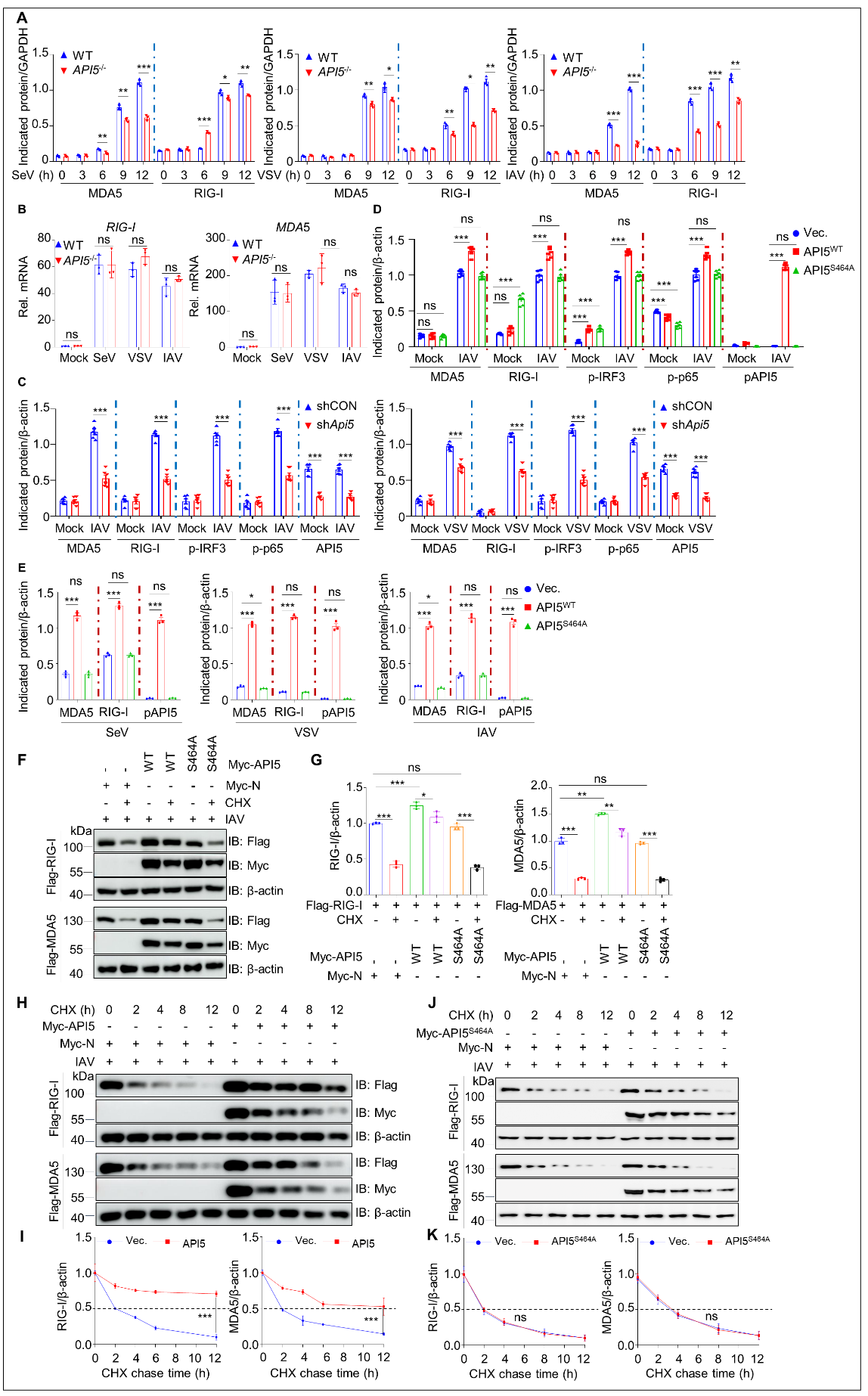
**

**Figure S12.** API5 represses the degradation of RIG-I and MDA5 through phosphorylation. (A) Data from Figure 7A were quantified and presented as the ratio of MDA5 to GAPDH, and RIG-I to GAPDH. (B) qPCR analysis of *RIG-I* and *MDA*5 mRNA in WT and *API5*^-/-^ A549 cells infected with SeV, VSV, or IAV for 12 h. (C and D) Data from Figure 7B, D were quantified and presented as the ratio of MDA5 to β-actin, RIG-I to β-actin, p-IRF3 to β-actin, p-p65 to β-actin and pAPI5 to β-actin. (E) Data from Figure 7C were quantified and shown as the ratio of MDA5 to β-actin, RIG-I to β-actin and pAPI5 to β-actin. (F) Immunoblot analysis of RIG-I and MDA5 in HEK293T cell co-transfected with Flag-RIG-I or Flag-MDA5 and empty vector, or plasmid encoding API5^WT^ or API5^S464A^ for 18 h, followed by infection with IAV for 12h and treatment with cycloheximide (CHX, 100 μg/mL) for 6 h. The cellular lysates were used for immunoblotting analysis with indicated antibodies. (G) Data from (F) were quantified and shown as the ratio of MDA5 to β-actin and RIG-I to β-actin. (H-K) API5 prolongs the half-life of RIG-I and MDA5 through phosphorylation. Flag-tagged RIG-I or MDA5 and Myc-API5 (H) or mutant Myc-API5^S464A^ (J) were co-transfected into HEK293T cells for 18 h, followed by IAV infection for 12 h. The resultant cells were treated with CHX (100 μg/mL) for indicated time points, and then the lysates were subjected to immunoblotting with indicated antibodies. (I and K) Data from (H) and (J) were quantified and presented as the ratio of MDA5 to β-actin and RIG-I to β-actin. For F, H, J, data are one representative of three biological replicates. Data represent the mean ± SEM (n ≥ 3 biological replicates). For A-E and G, statistical significance was analyzed by unpaired two-tailed Student’s t test. For I and K, statistical significance was analyzed by two-way Anova. (**p* < 0.05; ***p* < 0.01; ****p* < 0.001; ns, no significant).


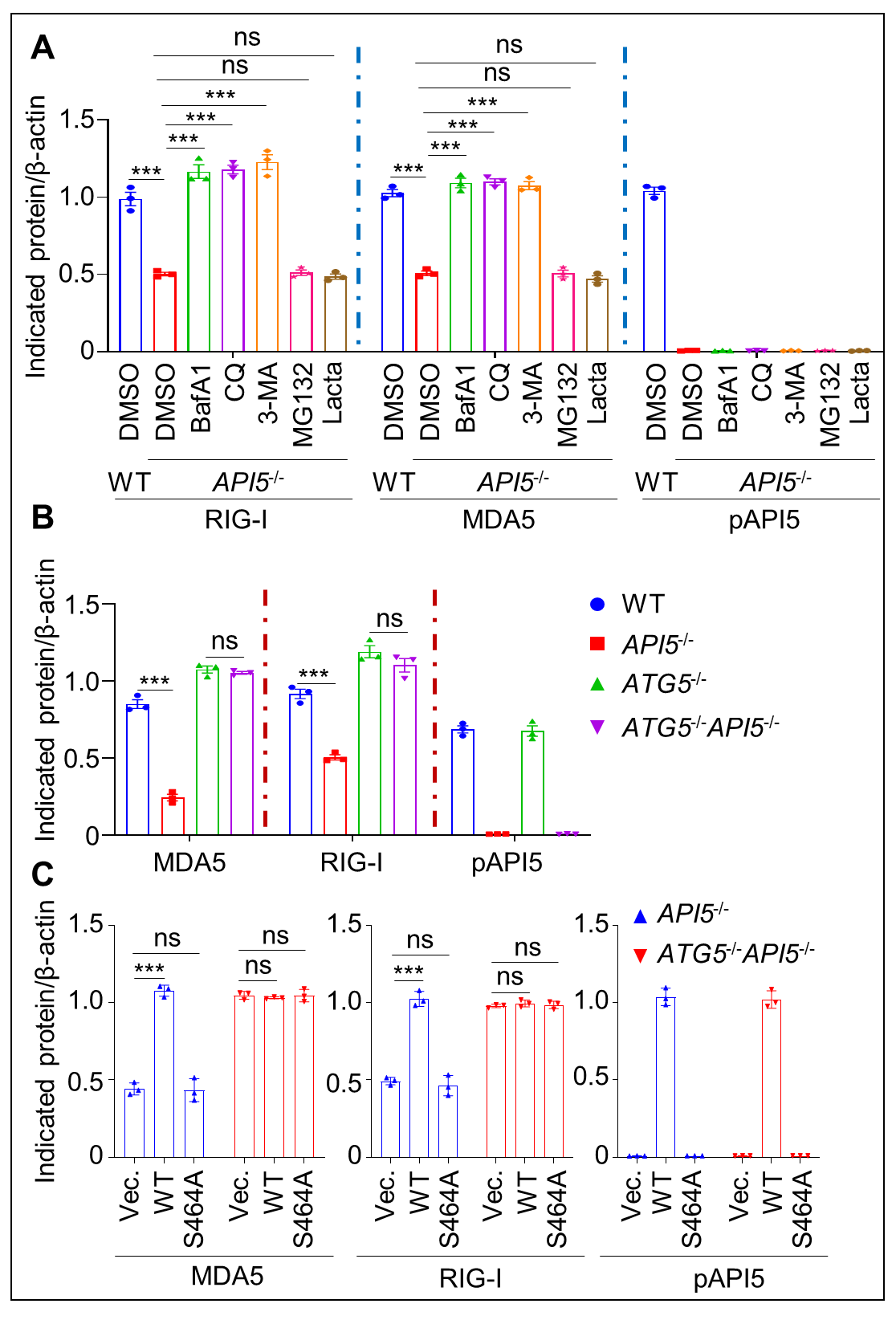


**Figure S13.** Densitometric analysis of immunoblots presented in Figure 7E-G. Data were quantified and shown as the ratio of RIG-I to β-actin, MDA5 to β-actin and pAPI5 to β-actin. Data represent the mean ± SEM (n = 3 biological replicates). Statistical significance was determined by unpaired two-tailed Student’s t-test. (****p* < 0.001; ns, no significant).


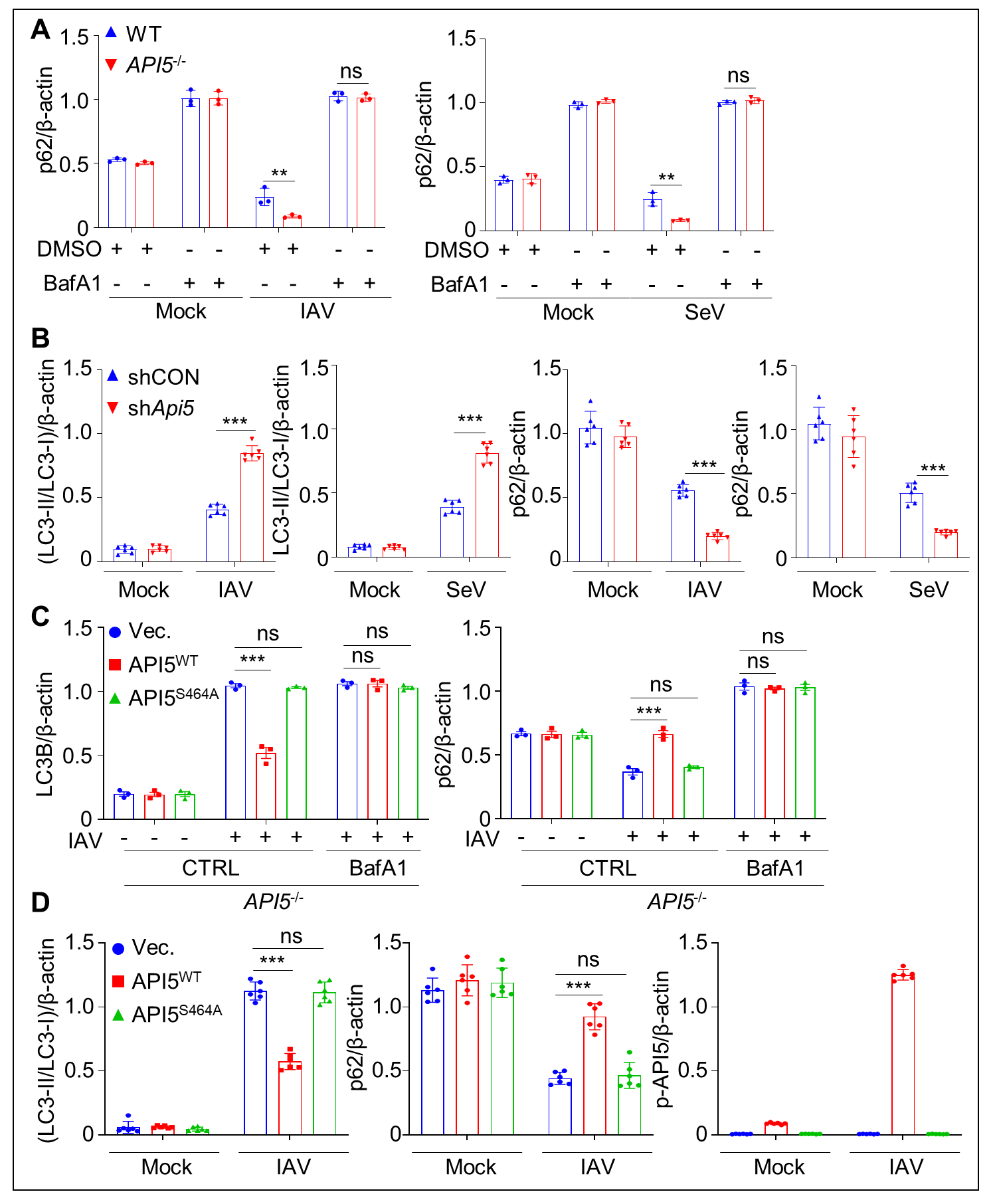


**Figure S14.** Densitometric analysis of immunoblots presented in Figure 8B, C, H, I. (A) Data from Figure 8B were quantified and shown as the ratio of p62 to β-actin. (B) Data from Figure 8C were quantified and shown as the ratio of LC3-II/LC3-I to β-actin. (C) Data from Figure 8H were quantified and shown as the ratio of LC3B to β-actin and p62 to β-actin. (D) Data from Figure 8I were quantified and shown as the ratio of LC3-II/LC3-I to β-actin, p62 to β-actin and pAPI5 to β-actin. Data represent the mean ± SEM (n ≥ 3 biological replicates). Statistical significance was determined by unpaired two-tailed Student’s t-test. (***p* < 0.01; ****p* < 0.001; ns, no significant).


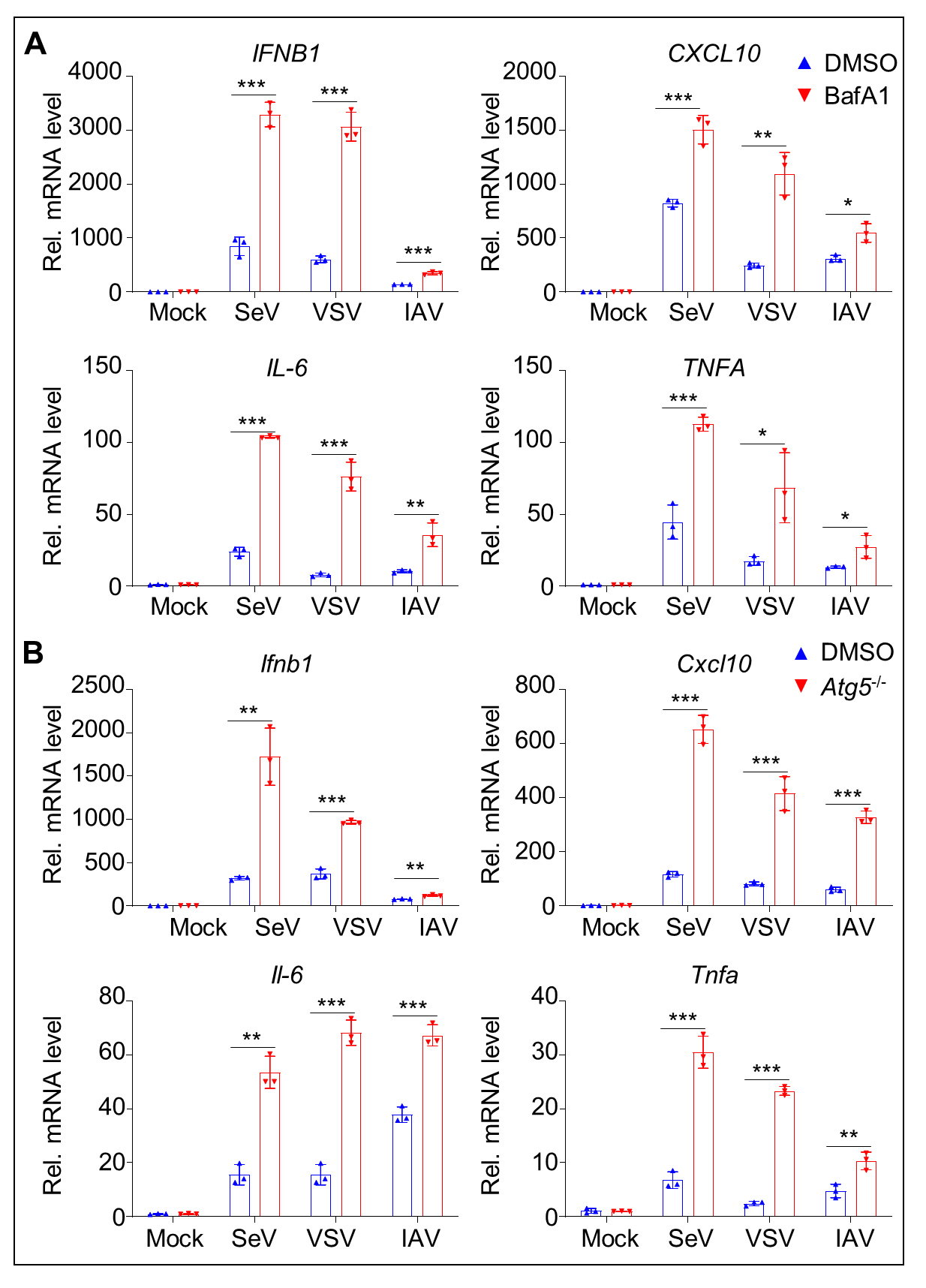


**Figure S15.** Inhibition of autophagy contributes to antiviral immune response. (A) qPCR analysis of *IFNB1*, *CXCL10*, *IL-6* and *TNFA* mRNA in A549 cells treated with DMSO and autophagy inhibitor bafilomycin A1 (BafA1; 200 nM) for 12 h followed by SeV (25 HA units), VSV (MOI = 0.1), or IAV (MOI = 3) infection for 9 h. (B) qPCR analysis of *Ifnb1*, *Cxcl10*, *Il*-*6* and *Tnfa* mRNA in WT and *Atg5*^-/-^ mouse embryo fibroblasts (MEF) cells with SeV (25 HA units), VSV (MOI = 0.1), or IAV (MOI = 3) infection for 9 h. Data were shown as mean ± SEM (n = 3 biological replicates). Statistical significance was analyzed by unpaired two-tailed Student’s t test. (**p* < 0.05; ***p* < 0.01; ****p* < 0.001).


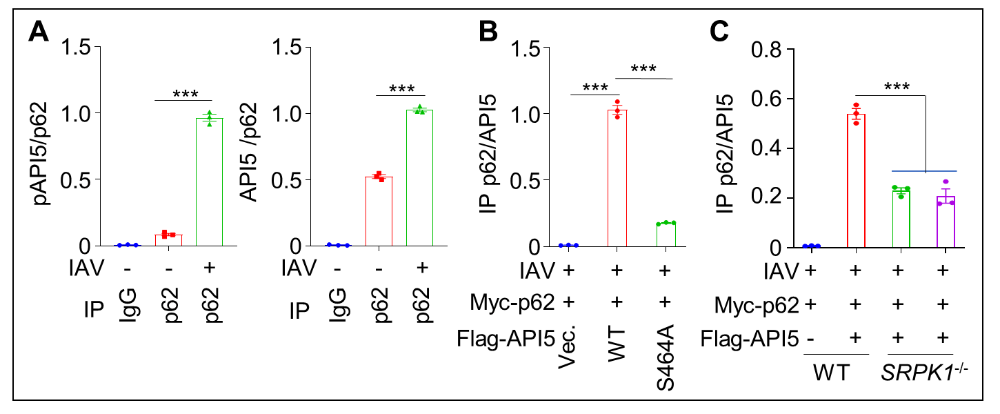


**Figure S16.** Densitometric quantification of immunoprecipitation (IP) immunoblots shown in Figure 9A-C. (A) Data from Figure 9A were quantified and shown as the ratio of pAPI5 to p62 in IP. (B and C) Data from Figure 9B (B) and Figure 9C (C) were quantified and presented as the ratio of p62 to API5 in IP. Data represent mean ± SEM (n = 3 biological replicates). Statistical significance was determined by unpaired two-tailed Student’s t-test. (****p* < 0.001).


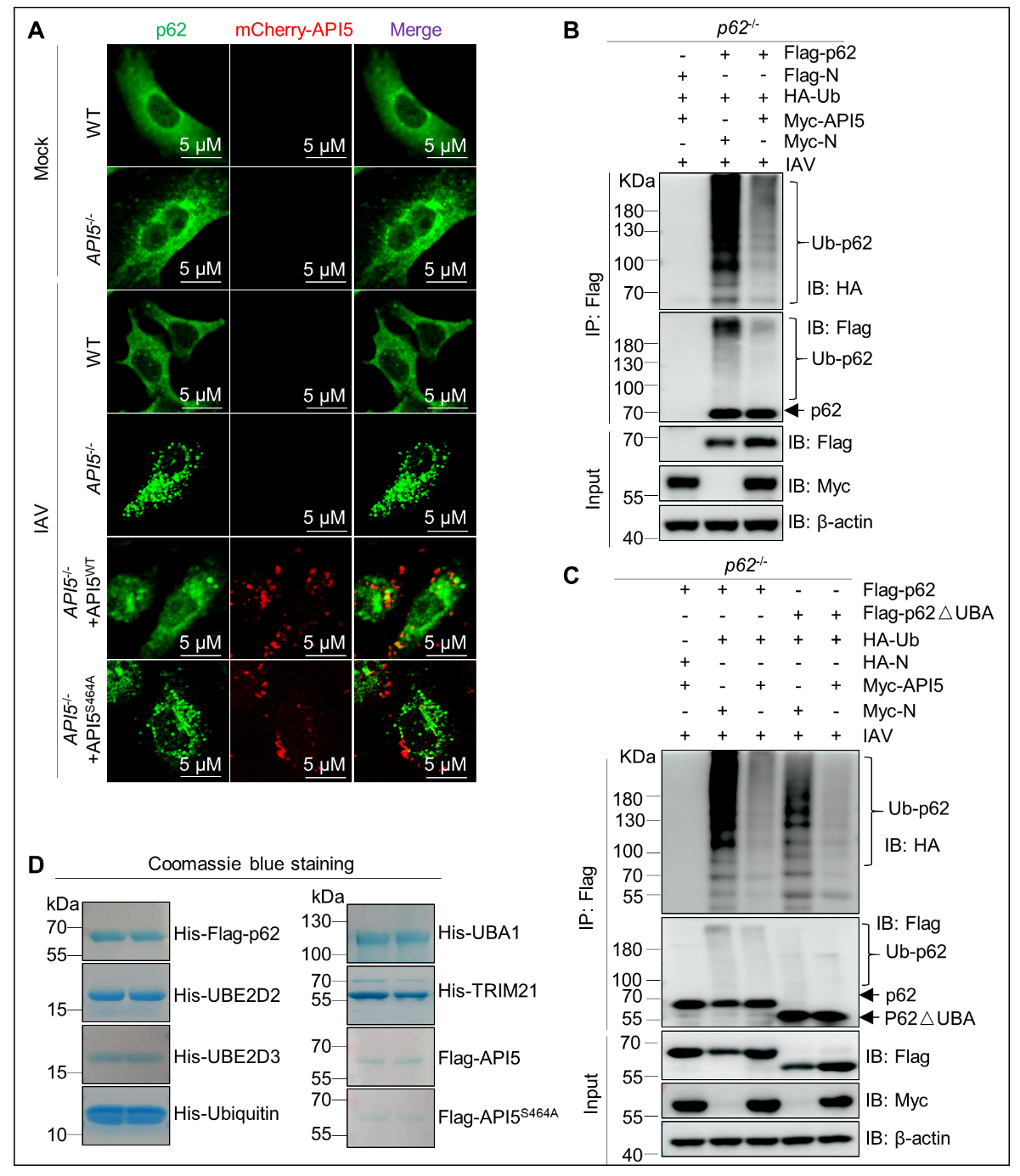


**Figure S17.** API5 inhibits p62 ubiquitination to promote p62 aggregates. (A) Confocal analysis of IAV-induced p62 aggregates in WT and *API5*^-/-^ A549 cells transfected with mCherry- API5^WT^ or API5^S464A^ for 24 h, followed by infection with IAV for 12 h. (B and C) API5 overexpression reduces ubiquitination of transfected p62^WT^ and p62△UBA. Flag-p62 (B) or Flag-p62△UBA (C), HA-Ub and Myc-API5 were separately co-transfected into *p62*^-/-^ HEK293T cells for 24 h, followed by infection with IAV for 12 h. The lysates were subjected to immunoprecipitation using Anti-FLAG® M2 Affinity Gel. Ubiquitinated Flag-p62 and Flag-p62△UBA were eluted by Flag peptides. The eluates were used to immunoblotting assays with indicted antibodies. (D) Brilliant Blue staining of purified His-tagged recombinant proteins Flag-p62, UBE2D2, UBE2D3, Ubiquitin, TRIM21, UBA1 from *E. coli* BL21 and Flag-tagged API5 andAPI5^S464A^ from HEK293T cells. For A-C, data are one representative of three biological replicates.


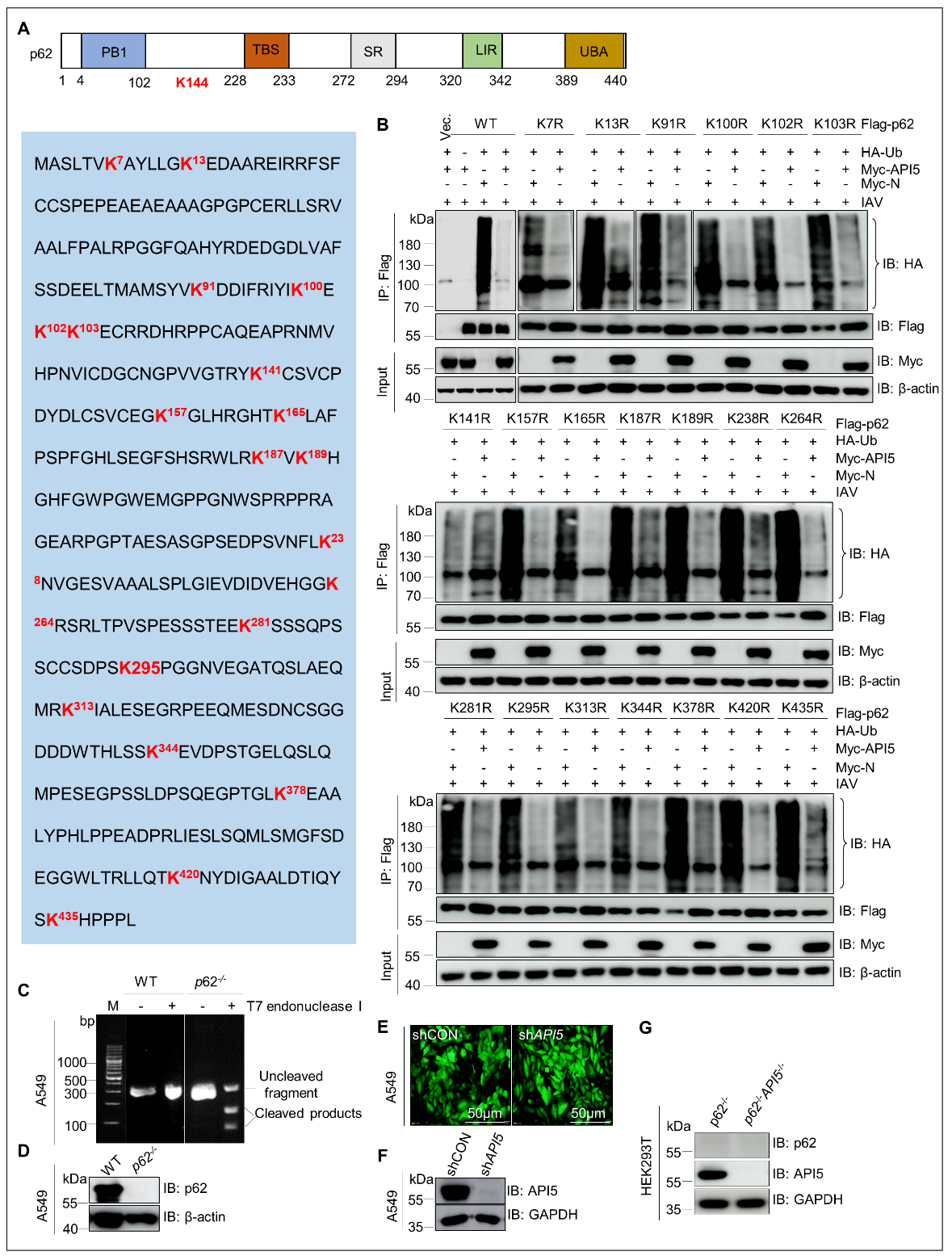


**Figure S18.** API5 deubiquitinates p62 at K141. (A) Schematic representation of Flag-p62 (Top) and the residual sequence of p62 (Bottom). The Lysine residues were displayed in red boldface. (B) Western blotting analysis of different mutants p62 ubiquitination regulated by API5. Different Flag-p62 mutants bearing single lysine (K)-to-arginine (R) substitution and Myc-API5 were co-transfected into HEK293T cells for 24h, followed by infection with IAV for 12 h. The lysates were immunoprecipitated with Anti-FLAG® M2 Affinity Gel and immunoblotting with indicated antibodies. (C and D) Identification of *p62*^-/-^ A549 cells. The genomic DNA and cellular lysates from WT and *p62*^-/-^ A549 cells were separately subjected to T7 endonuclease I (C), and western blotting (D). (E and F) The generation of *API5* knockdown *p62*^-/-^ A549 cells. *p62*^-/-^ shCON and *p62*^-/-^ sh*API5* A549 cells were observed and imaged by fluorescence microscopy (E), and the lysates were immunoblotted using anti-API5 rabbit pAb (F). (G) Western blotting analysis of p62 and API5 in *p62*^-/-^ and *p62*^-/-^*API5*^-/-^ HEK293T cells. For B-G, data are one representative of three biological replicates.


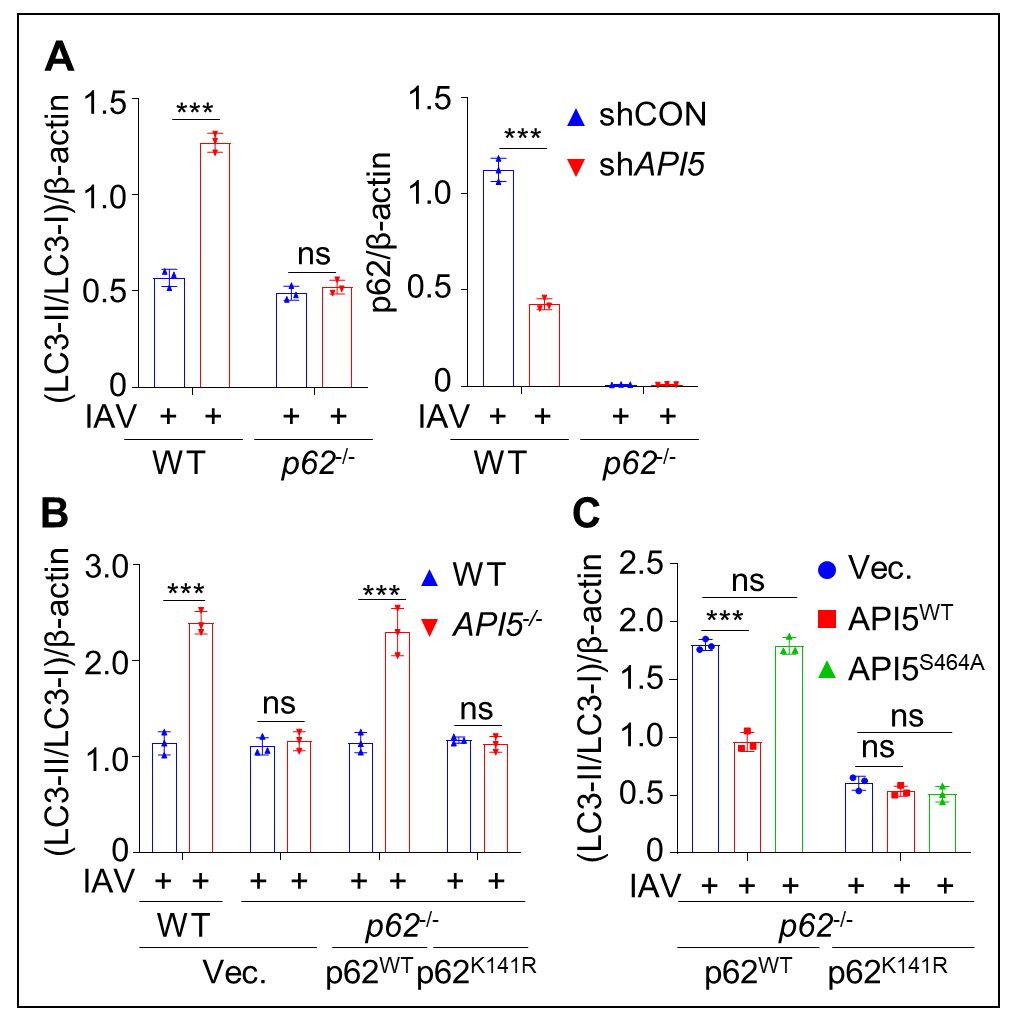


**Figure S19.** Densitometric analysis of immunoblots from Figure 9J-L. (A) Data from Figure 9J were quantified and shown as the ratio of LC3-II/LC3-I to β-actin and p62 to β-actin. (B and C) Data from Figure 9K (B) and Figure 9L (C) were quantified and presented as the ratio of LC3-II/LC3-I to β-actin. Data represent mean ± SEM (n = 3 biological replicates). Statistical significance was determined by unpaired two-tailed Student’s t-test. (****p* < 0.001; ns, no significant).

**
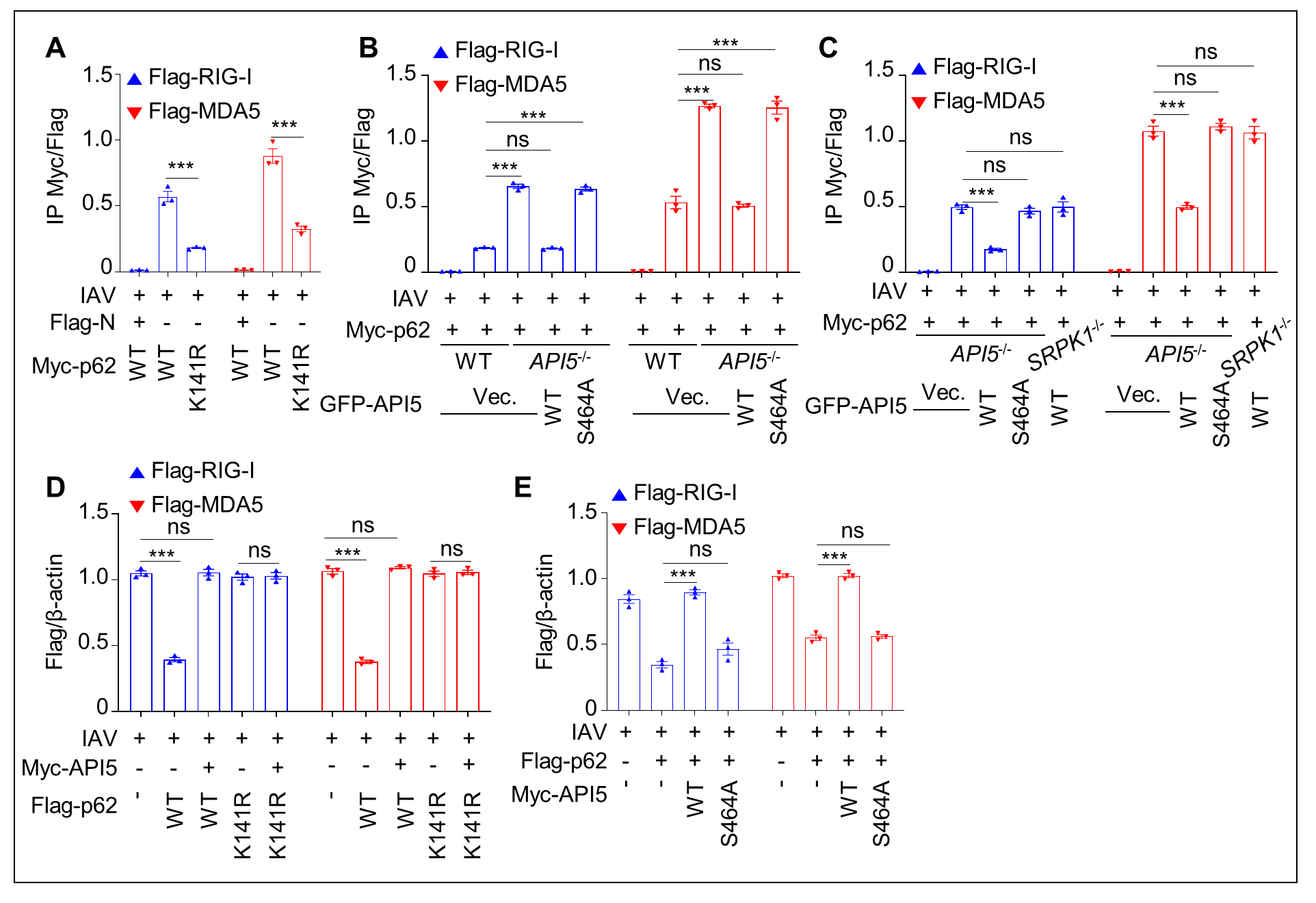
Figure S20.** Densitometric quantification of protein band intensities in immunoblots presented in Figure 10A-E. (A-C) Data from Figure 10A-C were quantified and shown as the ratio of Myc-p62 (Myc) to Flag-RIG-I or Flag-MDA5 (Flag) in IP. (D and E) Data from Figure 10 D and E were quantified and presented as the ratio of Flag-RIG-I or Flag-MDA5 (Flag) to β-actin. Data represent mean ± SEM (n = 3 biological replicates). Statistical significance was determined by unpaired two-tailed Student’s t-test. (****p* < 0.001; ns, no significant).

**
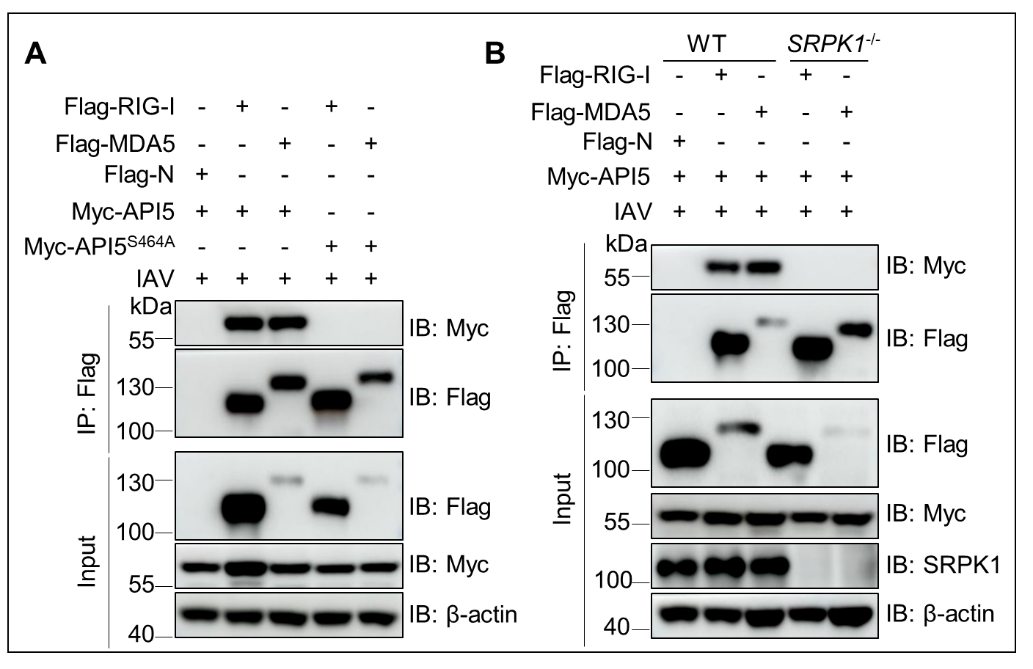
**

**Figure S21.** Phosphorylation of API5 is essential for binding to both RIG-I and MDA5. (A) Phosphorylated API5 interacts with transfected RIG-I/MDA5. Flag-RIG-I, Flag-MDA5 and Myc-API5 or Myc-API5^S464A^ were co-transfected into HEK293T cells for 24 h, followed by infection with IAV for 12 h. The lysates were subjected to immunoprecipitation and immunoblotting. (B) SRPK1 is required for API5 to associate with both RIG-I and MDA5. Flag-RIG-I, Flag-MDA5, and Myc-API5 were separately co-transfected into WT and *SRPK1*^-/-^ HEK293T cells for 36 h. The lysates were subjected to immunoprecipitation and immunoblotting. Data are one representative of three biological replicates.


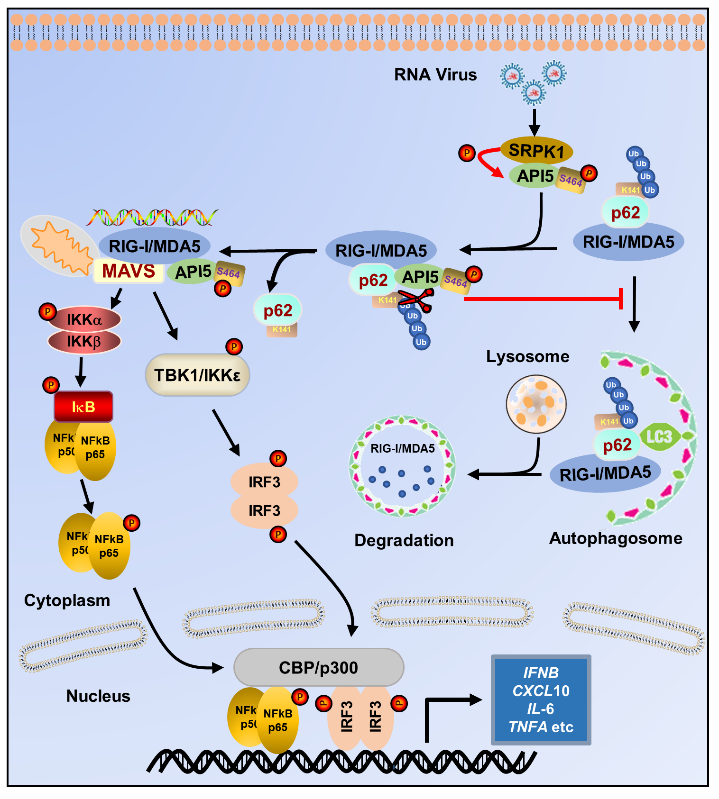


**Figure S22.** The working model of phosphorylated API5 at S464 site to drive innate antiviral defenses. RNA virus infection promotes SRPK1-mediated API5 phosphorylation at S464. Subsequently, phosphorylated API5 interacts with p62, resulting in the de-ubiquitination of p62 at residue K141, thereby inhibiting the association of p62 with the cytoplasmic viral RNA sensors RIG-I and MDA5 and preventing p62-mediated autophagic degradation of both RIG-I and MDA5. This process ultimately promotes innate antiviral immunity.

**Table S1. Plasmid information used in this study**

| VECTOR | SOURCE | IDENTIFIER |
| --- | --- | --- |
| IFN-β luciferase-reporter | Jihui Ping (Nanjing Agricultural University) | N/A |
| ISRE luciferase-reporter | Beyotime Biotechnology | Cat#D2179 |
| NF-κB luciferase-reporter | Beyotime Biotechnology | Cat#D2206 |
| PX459-API5 | This paper | N/A |
| pSIREN-API5 | This paper | N/A |
| PX459-SRPK1 | This paper | N/A |
| PX459*-*SQSTM1 | Lab stored | N/A |
| PX459-ATG5 | Lab stored | N/A |
| pCMV-Flag*-*RIG-I | Lab stored | N/A |
| pCMV-Flag-MDA5 | Lab stored | N/A |
| pCMV-Flag-API5 | This paper | N/A |
| pCMV-Flag-API5^S464A^ | This paper | N/A |
| pCMV-Myc-API5 | This paper | N/A |
| pCMV-Myc-API5^S464A^ | This paper | N/A |
| pEGFP-API5 | This paper | N/A |
| pEGFP-API5^S464A^ | This paper | N/A |
| mCherry-API5 | This paper | N/A |
| mCherry-API5^S464A^ | This paper | N/A |
| pCMV-Flag-API5 (1-320 aa) | This paper | N/A |
| pCMV-Flag-API5 (1-400 aa) | This paper | N/A |
| pCMV-Flag-API5 (321-524 aa) | This paper | N/A |
| pCMV-Flag-GST-API5 (401-453 aa) | This paper | N/A |
| pCMV-Flag-GST-API5 (401-475 aa) | This paper | N/A |
| pCMV-Flag-GST-API5 (476-524 aa) | This paper | N/A |
| pCMV-Flag-SRPK1 | This paper | N/A |
| pCMV-Flag-MAPK7 | This paper | N/A |
| pCMV-Flag-MAPK12 | This paper | N/A |
| pCMV-Flag-MAPK14 | This paper | N/A |
| pCMV-Flag-CLK1 | This paper | N/A |
| pCMV-Flag-DYRK2 | This paper | N/A |
| pCMV-Flag-HIPK2 | This paper | N/A |
| pCMV-Flag-GSNK1A1 | This paper | N/A |
| pCMV-Flag-GSNK1D1 | This paper | N/A |
| pCMV-Flag-GSNK1G3 | This paper | N/A |
| pCMV-Flag-GSNK2A1 | This paper | N/A |
| pCMV-Flag-GSNK3A | This paper | N/A |
| pCMV-Flag-GSNK3B | This paper | N/A |
| pCMV-Flag-CKB | This paper | N/A |
| pCMV-Flag-NEK6 | This paper | N/A |
| pCMV-Flag-PRKCI | This paper | N/A |
| pCMV-Flag-PRKCD | This paper | N/A |
| pCMV-Flag-ADPGK1 | This paper | N/A |
| pCMV-Flag-MARK1 | This paper | N/A |
| pCMV-Myc-CDK1 | This paper | N/A |
| pCMV-Myc-CDK2 | This paper | N/A |
| pCMV-Flag-CDK3 | This paper | N/A |
| pCMV-Myc-CDK4 | This paper | N/A |
| pCMV-Flag-CDK5 | This paper | N/A |
| pCMV-Myc-CDK6 | This paper | N/A |
| pCMV-Flag-CDK8 | This paper | N/A |
| pCMV-Myc-CDK9 | This paper | N/A |
| pCMV-Myc-PDPK1 | Lab stored | N/A |
| pCMV-HA-Ub | Lab stored | N/A |
| pCMV-Myc-p62 | Lab stored | N/A |
| pCMV-Flag-p62 | Lab stored | N/A |
| pCMV-Flag*-* p62^K7R^ | This paper | N/A |
| pCMV-Flag- p62^K13R^ | This paper | N/A |
| pCMV-Flag- p62^K91R^ | This paper | N/A |
| pCMV-Flag- p62^K100R^ | This paper | N/A |
| pCMV-Flag- p62^K102R^ | This paper | N/A |
| pCMV-Flag p62^K103R^ | This paper | N/A |
| pCMV-Flag- p62^K141R^ | This paper | N/A |
| pCMV-Flag- p62^K157R^ | This paper | N/A |
| pCMV-Flag- p62^K165R^ | This paper | N/A |
| pCMV-Flag- p62^K187R^ | This paper | N/A |
| pCMV-Flag- p62^K189R^ | This paper | N/A |
| pCMV-Flag- p62^K238R^ | This paper | N/A |
| pCMV-Flag- p62^K264R^ | This paper | N/A |
| pCMV-Flag- p62^K281R^ | This paper | N/A |
| pCMV-Flag- p62^K295R^ | This paper | N/A |
| pCMV-Flag- p62^K313R^ | This paper | N/A |
| pCMV-Flag- p62^K344R^ | This paper | N/A |
| pCMV-Flag- p62^K378R^ | This paper | N/A |
| pCMV-Flag- p62^K420R^ | This paper | N/A |
| pCMV-Flag- p62^K435R^ | This paper | N/A |
| pGEX-4T-API5 | This paper | N/A |
| pET-28a-Flag- p62 | This paper | N/A |
| pET-28a-TRIM21 | Lab stored | N/A |
| pET-28a-SRPK1 | This paper | N/A |
| pET-28a-SRPK1^K109A^ | This paper | N/A |
| pET-28a-Ub | Lab stored | N/A |
| pET-28a-UBA1 | Lab stored | N/A |
| pET-28a-UBE2D2 | This paper | N/A |
| pET-28a-UBE2D3 | This paper | N/A |
| Flag-MAVS | Lab stored | N/A |
| Flag-TBK1 | Lab stored | N/A |
| Flag-IRF3 | Lab stored | N/A |
| Flag-TRAF6 | Lab stored | N/A |
| Flag-IKKα | Lab stored | N/A |
| Flag-IKKβ | Lab stored | N/A |
| Flag-p65 | Lab stored | N/A |

**Table S2. Primers used for quantitative PCR**

| Gene | Forward sequence (5 ʹ-3 ʹ) | Reverse sequence (5 ʹ-3 ʹ) |
| --- | --- | --- |
| *GAPDH* | ATGACATCAAGAAGGTGGTG | CATACCAGGAAATGAGCTTG |
| *IFNB*1 | TTGTTGAGAACCTCCTGGCT | TGACTATGGTCCAGGCACAG |
| *CXCL*10 | GGTGAGAAGAGATGTCTGAATCC | GTCCATCCTTGGAAGCACTGCA |
| *IL-*6 | AGACAGCCACTCACCTCTTCAG | TTCTGCCAGTGCCTCTTTGCTG |
| *TNFα* | GCCGCATCGCCGTCTCCTAC | CCTCAGCCCCCTCTGGGGTC |
| m*Gapdh* | GTCAAGGCCGAGAATGGGAA | CTCGTGGTTCACACCCATCA |
| m*Api5* | CTGTACCGCAACTACGGCAT | AATCGCTTTTCCTTGGTGCC |
| m*Ifnb*1 | TCCTGCTGTGCTTCTCCACCACA | AAGTCCGCCCTGTAGGTGAGGTT |
| m*Cxcl*10 | ATCATCCCTGCGAGCCTATCCT | GACCTTTTTTGGCTAAACGCTTTC |
| m*Il*6 | TCTGCAAGAGACTTCCATCCAGTTGC | AGCCTCCGACTTGTGAAGTGGT |
| m*Tnfa* | ACTGAACTTCGGGGTGATCG | TCTTTGAGATCCATGCCGTTG |

**Table S3. Key Resources Table**

| REAGENT or RESOURCE | SOURCE | IDENTIFIER |
| --- | --- | --- |
| Antibodies | | |
| Mouse control IgG (4 μg/sample) | Beyotime Biotechnology | Cat#A7028 |
| Rabbit control IgG (4 μg/sample) | Beyotime Biotechnology | Cat#A7016 |
| HRP-conjugated goat-anti mouse IgG (1:5000 dilution) | Kirkegaard & Perry Laboratories | Cat#074-1806 |
| HRP-conjugated goat-anti rabbit IgG (1:8000 dilution) | Kirkegaard & Perry Laboratories | Cat#074-1506 |
| Alexa Fluor 546-conjugated donkey anti-rabbit IgG (1:500 dilution) | Invitrogen | Cat#A10040 |
| Alexa Fluor 546-conjugated donkey anti-mouse (1:500 dilution) | Invitrogen | Cat#A10036 |
| FITC-labeled goat anti-rabbit IgG (1:400 dilution) | Kirkegaard & Perry Laboratories | Cat#172-1506 |
| FITC-labeled goat anti-mouse IgG (1:400 dilution) | Kirkegaard & Perry Laboratories | Cat#172-1806 |
| 4ʹ,6ʹ-diamidino-2-phenylindole (1mg/mL, 1:1000 dilution) | BioFroxx | Cat#28718-90-3 |
| Rabbit anti-Flag Tag (1:1000 dilution) | HuaAn Biotechnology | Cat#0912 |
| Mouse anti-Flag Tag (1:2000 dilution) | Sigma-Aldrich | Cat#F1804; RRID: AB_262044 |
| Rabbit anti-Myc Tag (1:1000 dilution) | HuaAn Biotechnology | Cat#R1208 |
| Mouse anti-GFP Tag (1:2000 dilution) | Santa Cruz Biotechnology | Cat#sc9996; RRID: AB_627695 |
| Mouse anti-HA Tag (1:5000 dilution) | Sigma-Aldrich | Cat#H3663; RRID: AB_262051 |
| Rabbit anti-His Tag (1:2000 dilution) | HuaAn Biotechnology | Cat#0812 |
| Mouse anti-GST Tag (1:2000 dilution) | HuaAn Biotechnology | Cat#EM80701 |
| Mouse anti-β-Actin (1:3000 dilution) | HuaAn Biotechnology | Cat#M1210 |
| Mouse anti-Histone H3 (1:5000 dilution) | HuaAn Biotechnology | Cat#M1306 |
| Rabbit anti-GAPDH (1:1000 dilution) | GoodHere Technology | Cat#AB-P-R001 |
| Mouse anti-influenza viral proteins (1:1000 dilution) | Lin et al., 2023 | N/A |
| Rabbit anti-API5 (1:2000 dilution) | Abcam | Cat# ab65836; RRID: AB_1141081 |
| Mouse anti-API5 for IP (4 μg/sample) | Santa Cruz Biotechnology | Cat#sc-393341 |
| mouse anti-API5 for IFA (1:100 dilution) | Santa Cruz Biotechnology | Cat#sc-101203; AB_2242878 |
| Rabbit anti-p-API5(Ser464) (1:1000 dilution) | Customed by ABclonal | Cat#AP1247 |
| Rabbit anti-SRPK1 (1:1000 dilution) | ABclonal | Cat#A5854; RRID: AB_2766604 |
| Rabbit anti-RIG-I for IP (4 μg/sample) | Proteintech | Cat#20566-1-AP; RRID: AB_10700006 |
| Mouse anti-RIG-I for IFA (1:100 dilution) | Santa Cruz Biotechnology | Cat# sc-376845; RRID: AB_2732794 |
| Rabbit anti-MDA5 (1:1000 dilution) | Cell Signaling Technology | Cat#5321; RRID: AB_10694490 |
| Rabbit anti-MDA5 for IFA (1:100 dilution) | ABclonal | Cat#A13645; AB_2760507 |
| Mouse anti-Ubiquitin (1:1000 dilution) | Cell Signaling Technology | Cat#3936; RRID: AB_331292 |
| Rabbit anti-TBK1 (1:2000 dilution) | Cell Signaling Technology | Cat#3504; RRID: AB_2255663 |
| Rabbit anti-phospho-TBK1 (1:2000 dilution) | Cell Signaling Technology | Cat#5483; RRID: AB_10693472 |
| Rabbit anti-IKKα/β (1: 1000 dilution) | HuaAn Biotechnology | Cat#ET1611-23 |
| Rabbit anti-phospho-IKKα/β (1:1000 dilution) | Cell Signaling Technology | Cat#2697; RRID: AB_2079382 |
| Rabbit anti-IRF3 (1:2000 dilution) | Proteintech | Cat#11312-1-AP; RRID: AB_2127004 |
| Rabbit anti-phospho-IRF3 (1:1000 dilution) | Signaling way antibody | Cat#13786 |
| Rabbit anti-p65 (1:1000 dilution) | HuaAn Biotechnology | Cat#ET1603-12 |
| Rabbit anti-phospho-p65 (1:3000 dilution) | Cell Signaling Technology | Cat#3033; RRID: AB_331284 |
| Rabbit anti-phospho-IκBα (1:1000 dilution) | Cell Signaling Technology | Cat#2859; RRID: AB_561111 |
| Mouse anti-p62 for IFA (1:200 dilution) | Cell Signaling Technology | Cat#88588; RRID: AB_2800125 |
| Rabbit anti-p62 (1:1000 dilution) | Abcam | Cat#ab109012; RRID: AB_2810880 |
| Mouse anti-p62 for IP (4 μg/sample) | Proteintech | Cat#66184-1-Ig; RRID: AB_2881579 |
| Rabbit anti-LC3B (1:1000 dilution) | Cell Signaling Technology | Cat#2775; RRID: AB_915950 |
| Rabbit anti-ATG5 (1:2000 dilution) | HuaAn Biotechnology | Cat#ET1611-38 |
| Bacterial and Virus Strains | | |
| eGFP-tagged Vesicular Stomatitis Virus (VSV) | Pinglong Xu (Zhejiang University) | N/A |
| Sendai Virus (SeV) | Jin jin (Zhejiang University) | N/A |
| A/Puerto Rico/8/34(PR8, H1N1) | Lin et al., 2023 | N/A |
| Adeno-associated virus 6 (AAV6) with sh*Api5* | WZ Biosciences Inc. | N/A |
| AAV6 with scrambled shRNA control | WZ Biosciences Inc. | N/A |
| AAV6 expressing Flag-API5 | WZ Biosciences Inc. | N/A |
| AAV6 expressing Flag-API5^S464A^ | WZ Biosciences Inc. | N/A |
| Chemicals, Peptides, and Recombinant Proteins | | |
| Poly (I:C)-LMW (1 μg/well of a 12-well plate) | Invivogen | Cat#tlrl-picw |
| Poly (I:C)-HMW (1 μg/well of a 12-well plate) | Invivogen | Cat#tlrl-pic |
| Cycloheximide (CHX) (100 mg/mL) | MedChemExpress | Cat#HY-12320 |
| Bafilomycin A1 (BafA1) (200 nM) | MedChemExpress | Cat#HY-100558 |
| Chloroquine (CQ) (50 μM) | MedChemExpress | Cat#HY-17589A |
| 3-methyladenine (3-MA) (10 mM) | MedChemExpress | Cat#HY-19312 |
| MG-132 (10 μM) | MedChemExpress | Cat#HY-13259 |
| Lactacystin (Lacta) (10 μM) |  | Cat#HY-16594 |
| SRPIN340 (10 μM) | MedChemExpress | Cat#HY-13949 |
| Puromycin (10 μg/mL) | Invivogen | Cat#58-58-2 |
| Penicillin-streptomycin (1:1000 dilution) | Sigma-Aldrich | Cat#P0781 |
| phenylmethylsulfonyl fluoride (PMSF) (1 mM) | Solarbio | Cat#P8340 |
| N-ethylmaleimide (NEM) (20 nM) | Sigma-Aldrich | Cat#E3876 |
| β-glycerophosphate disodium salt hydrate (10 mM) | Sigma-Aldrich | Cat#G9422-10G |
| 1,4-dithio-DL-threitol (DTT) (2 mM) | Solarbio | Cat#3483-12-3 |
| Adenosine 5'-triphosphate (ATP) (5 mM) | Beyotime Biotechnology | Cat#D7378-1ml |
| Anti-FLAG® M2 Affinity Gel (10 μL/sample) | Sigma-Aldrich | Cat#A2220 |
| Protein A/G PLUS-Agarose (60 μL/sample) | Santa Cruz Biotechnology | Cat#sc-2003 |
| GST resin (60 μL/sample) | Thermo Fisher | Cat#16100 |
| Ni-NTA agarose (500 μL/sample) | QIAGEN | Cat#30210 |
| 3× Flag Peptide (200 μg/mL) | Beyotime Biotechnology | Cat# P9801 |
| JetPRIME transfection reagent (2 μL: 1 μg DNA) | Polyplus | Cat#PT-114-15 |
| Biobest transfection reagent (2 μL: 1 μg DNA) | BioBEST Biotechnology | Cat#BB0002 |
| Reduced glutathione (2 mg/mL) | Sangon Biotech | Cat#A100399-0005 |
| Critical Commercial Assays | | |
| Dual-luciferase reporter assay kit | Beyotime Biotechnology | Cat#RG027 |
| PrimeScript™ RT reagent Kit | Takara | Cat#RR047A |
| TB Green® Premix Ex Taq™ | Takara | Cat#RR420A |
| Mouse IFN-β ELISA Kit | ABclonal | Cat#RK00420 |
| MouseCXCL10/IP10 ELISA Kit | ABclonal | Cat#RK00056 |
| Mouse IL-6 ELISA Kit | ABclonal | Cat#RK00008 |
| Mouse TNF-α ELISA Kit | ABclonal | Cat#RK00027 |
| Human IFN-β ELISA Kit | ABclonal | Cat#RK00030 |
| Human CXCL10/IP10 ELISA Kit | ABclonal | Cat#RK00054 |
| Human IL-6 ELISA Kit | ABclonal | Cat#RK00004 |
| 2 × Phanta Flash Master Mix | Vazyme Biotech Co., Ltd | Cat#P510 |
| T7 endonuclease I | Vazyme Biotech Co., Ltd | Cat#EN303 |
| Experimental Models: Cell Lines | | |
| A549 | ATCC | CRM-CCL-185 |
| HEK293T | ATCC | CRL-11268 |
| THP-1 | Cell Bank of Chinese Academy of Sciences | SCSP-567 |
| *API5*^-/-^ A549 | This paper | N/A |
| *API5*^-/-^ HEK293T | This paper | N/A |
| shCON THP-1 | This paper | N/A |
| sh*API5* THP-1 | This paper | N/A |
| *p62*^-/-^ A549 | This paper | N/A |
| *p62*^-/-^ HEK293T | Deng et al., 2022 | N/A |
| *p62*^-/-^ shCON A549 | This paper | N/A |
| *p62^-/-^* sh*API5* A549 | This paper | N/A |
| *p62*^-/-^ *API5*^-/-^ HEK293T | This paper | N/A |
| *SRPK1*^-/-^ HEK293T | This paper | N/A |
| Wild type (WT) A549 expressing GFP-LC3 | This paper | N/A |
| *API5*^-/-^ A549 expressing GFP-LC3 | This paper | N/A |
| *ATG5*^-/-^ HEK293T | Deng et al., 2022 | N/A |
| *Atg5*^-/-^ MEFs | Wei Liu (Zhejiang University) | N/A |
| *ATG5*^-/-^*API5*^-/-^ HEK293T | This paper | N/A |
| Experimental Models: Organisms/Strains | | |
| C57BL/6N-*Api5*^-/-^ Mice | Cyagen Biosciences Inc. | N/A |
| Wild-type C57BL/6 Mice | SLAC Laboratory Animal | N/A |
| Oligonucleotides | | |
| gRNA for *API5*:  ACCATCCAATATCACTTGAT | This paper | N/A |
| shRNA for *API5*:  CCCAGGCTGTTCATAATGTTA | This paper | N/A |
| gRNA for *SRPK1*:  TCCTGCAGTGCTTGCGCTCC | This paper | N/A |
| gRNA for *p62*:  AGGGCTTCTCGCACAGCCGC | Deng et al., 2022 | N/A |
| gRNA for *ATG5*:  AAATGTACTGTGATGTTCCA | Deng et al., 2022 | N/A |
| Primers for qRT-PCR, see Table S2 | This paper | N/A |
| Recombinant DNA | | |
| See Table S1 for the List of Recombinant DNA | This paper | N/A |
| Software and Algorithms | | |
| GraphPad Prism9 | GraphPad | https://www.graphpad.com/  scientific-software/prism/ |
| ImageJ | ImageJ | https://imagej.nih.gov/ij/ |
